# Effects of adipose-derived stromal cells and endothelial progenitor cells on adipose transplant survival and angiogenesis

**DOI:** 10.1371/journal.pone.0261498

**Published:** 2022-01-13

**Authors:** Fengshan Gan, Liu Liu, Qingzhu Zhou, Wenli Huang, Xinwei Huang, Xian Zhao

**Affiliations:** 1 Department of Plastic Surgery, Kunming Medical University, Kunming, China; 2 Key Laboratory of The Second Affiliated Hospital of Kuming Medical College, Kunming, China; Università degli Studi della Campania, ITALY

## Abstract

**Background:**

A paracrine mechanism is thought to mediate the proangiogenic capacity of adipose-derived stromal/stem cells (ASCs). However, the precise mechanism by which ASCs promote the formation of blood vessels by endothelial progenitor cells (EPCs) is unclear.

**Methods:**

The EPCs-ASCs cocultures prepared in different ratios were subjected to tube formations assay to verify whether ASCs could directly participate in the tube genesis. The supernatant from cultured ASCs was used to stimulate EPCs to evaluate the effects on the angiogenic property of EPCs, as well as capacity for migration and invasion. A coculture model with transwell chamber were used to explore the regulation of angiogenesis markers expression in EPCs by ASCs. We then mixed ASCs with EPCs and transplanted them with adipose tissue into nude mice to evaluate the effects on angiogenesis in adipose tissue grafts.

**Results:**

In the EPCs-ASCs cocultures, the tube formation was significantly decreased as the relative abundance of ASCs increased, while the ASCs was found to migrate and integrated into the agglomerates formed by EPCs. The supernatant from ASCs cultures promoted the migration and invasion of EPCs and the ability to form capillary-like structures. The expression of multiple angiogenesis markers in EPCs were significantly increased when cocultured with ASCs. *In vivo*, ASCs combined with EPC promoted vascularization in the fat transplant. Immunofluorescence straining of Edu and CD31 indicated that the Edu labeled EPC did not directly participate in the vascularization inside the fat tissue.

**Conclusions:**

ADSC can participate in the tube formation of EPC although it cannot form canonical capillary structures. Meanwhile, Soluble factors secreted by ASCs promotes the angiogenic potential of EPCs. ASCs paracrine signaling appears to promote angiogenesis by increasing the migration and invasion of EPCs and simultaneously upregulating the expression of angiogenesis markers in EPCs. The results of *in vivo* experiments showed that ASCs combined with EPCs significantly promote the formation of blood vessels in the fat implant. Remarkably, EPCs may promote angiogenesis by paracrine regulation of endogenous endothelial cells (ECs) rather than direct participation in the formation of blood vessels.

## Background

Autologous fat transplantation technology was first introduced more than 100 years ago [1]. Due to its unique advantages and remarkable clinical effects, it has attracted increasing attention from experts in the field of plastic surgery. Autologous fat transplantation has numerous advantages such as the availability of source material, decreased risk for rejection, a natural feel, minimal trauma, reproducible results, and easy removal [2]. The major obstacle currently faced by autologous fat transplantation is autologous absorption. Most cases of autologous absorption occur in the first 3 months after transplantation, with the rate of absorption reaching 50%. The main reason for autologous absorption is the inability to expediently establish an effective blood supply to the fat graft. It is believed that distances > 2 mm between fat granules and the arterial blood vessels will result in insufficient nutrient supply to the transplanted fat [3]. Portions of the fat will become necrotic, liquefy, and become absorbed [4, 5]. Therefore, it is very important to establish a new blood supply that can provide sufficient nutrients for transplanted fat soon after transplantation.

In recent years, numerous studies have focused on the stem cells derived from fat. It has been shown that adipose-derived stem cells (ASCs) can promote the survival of autologous fat transplantation [6–8]. Yuan *et al* [9] demonstrated that the addition of exogenous ASCs during transplantation significantly increased the retention rate and the ratio of vascularized adipose tissue throughout the graft. Meanwhile, expression levels of vascular endothelial growth factor (VEGF) and hepatocyte growth factor (HGF) were higher in adipose grafts cotransplanted with ASCs.

The main mechanism of vascular repair in ischemic tissue requires the recruitment of endothelial progenitor cells (EPCs) in circulating blood [10]. When the body undergoes pathological changes such as trauma and ischemia, EPCs migrate and colonize the ischemic site, secrete pro-angiogenic factors, and promote the growth of collateral vessels to ischemic tissue. EPCs are also known to participate in the organization of angiogenesis and to promote tissue repair [11, 12].

We therefore cocultured ASCs and EPCs in order to observe the effects on EPC tube formation, migration, and invasion. This study aimed to investigate whether ASCs improve the survival of fat grafts by promoting EPC-mediated angiogenesis.

## Materials and methods

### Isolation and characterization of peripheral blood EPCs (PB-EPCs)

For the isolation of EPCs, peripheral blood (10 ml/kg) was obtained from New Zealand white rabbits that had been anesthetized with pentobarbital (0.01 ml/g body weight at 5 mg/ml in 5% ethanol/PBS, intraperitoneally) by puncturing the ear vein. Peripheral blood mononuclear cells (PBMC) were isolated by density gradient centrifugation with Ficoll-Plaque Plus (GE Healthcare, America). Mononuclear cells were then washed and plated on six-well plates that were coated with human fibronectin (Sigma, America) at a concentration of 3 μg/cm^2^ or rat tail collagen I (BD, America) at a concentration of 10 μg/cm^2^. Cells were supplemented with EGM-2 MV BulletKit medium (Lonza Corp, Switzerland). Mononuclear cells were incubated at 37°C, 5% CO2, and fed daily with EGM-2. At 3 to 7 days post-plating, adherent EPC were detached with 0.025% trypsinase containing 0.02% EDTA. The same procedure was performed for the subsequent 3 passages (approximately 1 week of culture). All experiments were performed with cells that were cyropreserved at passage 3.

EPCs isolated from rabbits were characterized based on their ability to take up FITC-acetylated low-density lipoprotein (FITC-Ac-LDL,) and bind to Dylight 594-*Ulex europaeus* agglutinin-1 (Dylight 594-UEA-1), as previously described. Briefly, P3 cultures of EPCs were seeded on 6-well plates and incubated with 20 μg/ml FITC-labeled Ac-LDL (Molecular Probes) for 4 h and 15 μg/ml Dylight 594-labeled UEA-1 (Vector) for 1 hour. After incubation, cells were washed with serum-free medium and detected under fluorescence microscopy. Immunostaining of endothelial cell marker CD31 (Novus, clone JC/70A) was performed as previously described. Briefly, EPC cultures were washed with phosphate-buffered saline (PBS) and fixed with 4% paraformaldehyde for 30 minutes, then blocked with 5% bovine serum albumin (BSA) at 37°C for 1 hour. Cells were then incubated with anti-CD31 monoclonal antibody (1:1000) at 4°C overnight. Cells were washed 4 times in PBS and stained for 1 h in the dark at room temperature with Alexa Fluor-594 goat anti-mouse antibody (1:1000; Molecular Probe). After 4 washes with PBS, the slides were mounted in medium containing DAPI (Vectashield).

### Isolation and culturing of adipose stromal cells

Adipose tissue was obtained from the thighs and groins of New Zealand white rabbits and cut into 2-mm pieces. Tissues were then washed twice with PBS containing 2% penicillin/streptomycin and once with DMEM/F12 medium. The cleaned fat blocks were transferred into a 15-ml centrifuge tube and agitated in 1 mg/ml Liberase TL solution (Roche) prepared in DMEM/F12 medium (Invitrogen) supplemented with 5% FBS, 100 units/ml penicillin, and 100 μg/ml streptomycin, for 1 h at 37°C. Single cells were obtained by filtering the solution through a 70-μm cell strainer. Samples were then centrifuged at 1000 rpm for 5 min to separate the stromal cell fraction from the adipocytes. The pellet was treated with red blood cell (RBC) lysis buffer to eliminate erythrocytes, then resuspended in EGM-2MV media. The ASC monolayers were split upon achieving 80% confluence and used at passage 3.

### Molecular phenotyping of ASCs and EPCs cultured in vitro

Quantitative real-time PCR (qRT-PCR) was performed to measure gene expression in EPCs and ASCs cultured *in vitro*. Total RNA was extracted from cultured cells and fresh isolated PBMC with Trizol reagent (Invitrogen), then converted into cDNA with a First Strand cDNA Synthesis kit (Thermo Scientific). qPCR was conducted using SYBR Green I Master mix (Applied Biosystems) with an ABI 7500 instrument. The primers for CD31, CD34, CD45, vWF, VEGFA, VEGFR1, VEGFR2, and ANGPT1(Angiopoietin-1) are summarized in Table 1. GAPDH was used as an internal control. All experiments were performed in triplicate. The results are expressed as mean ± SEM.

**Table 1:** Primer pairs for quantitative RT-PCR analysis of target rabbit genes.

Target gene	Forward (F) and reverse (R) primer sequence	Product length	NCBI accession
**CD31**	F: GCATTGGCAAGGTGGTGAAG	130	XM_008271715.2
			XM_008271716.2
	R: TGGCAGCTCACTTCTATGGC		
**CD34**	F: TCCCGGAAGACCTTGATTGC	128	XM_008268471.2
	R: TAAGGGTCTTCGCCCAGCC		
**CD45**	F: AAGACAACGGTGCAGGAAGG	134	XM_008268693.2
	R: CCCGGCAACAAACACTTCTG		
**vWF**	F: TGAAGCTCCAGCGCATTGAA	146	NM_001329088.1
	R: ATCTGGCAAGGTCCACACAT		
**VEGFA**	F: AGTTCGAGGAAAGGGCAAGG	157	XM_017345155.1
	R: ACGCGAGTCTGTGTTTTTGC		
**VEGFR1**	F: GTGCACGGTCAACAAGTTCC	80	XM_008275216.2
	R: TGTGGCGCATTGTTCTGTTG		
**VEGFR2**	F: GGTGCTTCTCCGTATCCTGG	222	NM_001195670.1
	R: GTAGTCTTTGCCACCCTGCT		
**ANGPT1**	F: TGTGCCCTCATGCTTACAGG	236	XM_008255827.2
	R: CTCGGTCTCCAGATCGCTTC		
**ANGPT2**	F: TGGCTGGGAAACGAGTTTGT	198	XM_002720896.3
	R: TGGTTGGCTGATGCTGCTTA		
**Tie2**	F: CAAGAACAACCCGGATCCCA	117	XM_017340937.1
	R: TCCTTCTTGATGCGTGCCTT		
**CDH5**	F: CAGGTACGAGATCGTGGTGG	120	XM_008257382.2
	R: GTCTGCGTGAAGATGGGGAA		
**CD90**	F: CCTGAGCCTCTCAAGACAGC	160	XM_002722718.3
	R: TGTGATGGCACTACACACGG		
**CD105**	F: AGCATGATCAGCAACGAGGT	182	XM_008251029.2
	R: GAGCTCACCTGCACAAAACC		
**CD73**	F: GCAGTTGAGGGTCGGATCAA	274	XM_002714557.3
	R: CTGAGTTGCTGATCCGAGTGA		
**CD44**	F: TCAAACAGCCATGCAGCAAC	180	XM_008269684.2
	R: CCACCATGGAAGCCAATCCT		
**CD29**	F: GAATGCCAAATGGGACACGC	111	XM_008274741.2
	R: AGGATTTTCACCGGCACTCA		
**GAPDH [31]**	F(PS182): TGACGACATCAAGAAGGTGGTG	120	NM_001082253
	R(PS183): GAAGGTGGAGGAGTGGGTGTC		

### Two-dimensional assessment of matrigel angiogenesis

To test the ability of EPCs-ADSs cocultures to form tubes in vitro, EPCs (Labeled with Calcein-AM) and ASCs (Labeled with DiD) were resuspended in EGM-2 basal medium (10% heat-inactivated FBS, without growth factors), then seeded at cell number ratios of 100:0, 80:20, 67:33, 50:50, 33:67, 20:80, and 0:100 onto 24-well plates precoated with thick Matrix gel (300 μl/well, Matrigel matrix growth factors reduced, BD). The final density of cells was 10^5^ cells/ml/well. The plates were incubated for 4h at 37°C and 5% CO_2_. Tube formation was observed with inverted fluorescence microscope. For each sample, a total of 6 pictures were randomly captured from three wells in parallel. Tube length and number of branches were measured with Image-Pro Plus software. The results are expressed as mean ± SEM.

To analyze the pro-angiogenic activity of ASCs, ASCs supernatant was collected after 48 h of cultivation and subjected to a two-dimensional angiogenesis assay. EPC supernatant was used as the internal control. EGM-2 basal medium (10% heat-inactivated FBS, without growth factors) and EGM-2 MV complete medium were used as culture controls. As described previously, 10^5^ EPCs were resuspended in 1 ml of ASC supernatant, EPC supernatant, EGM-2 basal medium, or EGM-2MV complete medium, then seeded into 24-well plates precoated with thick Matrix gel. Tube formation was analyzed with the methods described above.

### Coculture of ASCs and EPCs

The expression of angiogenesis markers in EPCs cocultured with ASCs was examined with qRT-PCR. EPCs were resuspended in EGM-2 basal medium, then seeded in in a total volume of 2.7 ml containing 5.4×10^5^ cells in 6-well plates. A total of 3×10^5^ ASCs that had been resuspended in 1.5 ml EGM-2 basal medium were added to each transwell insert (pore size 2 μm, Corning). The same volume of EGM-2 basal medium (10% heat-inactivated FBS, without growth factors) or EGM-2 MV complete medium was added to a different transwell insert as a control. After coculture for 24 h, the EPCs in the lower chamber were harvested for total RNA extraction with Trizol reagent (Invitrogen). RNA (2 μg) was reverse-transcribed using the First Strand cDNA Synthesis kit (Thermo scientific). QRT-PCR was conducted using SYBR Green I MasterMix (Applied Biosystems) with an ABI 7500 instrument. The primers for VEGFA, angiopoietin-1/2, VEGFR1, Tie-2, and GAPDH are shown in Table 1. Experiments were performed in triplicate for each gene. The results are expressed as mean±SEM.

### EPCs transmigration and invasion

The transwell migration assay was used to investigate the chemotactic ability of ASCs cocultured with EPCs. Briefly, 500 μl EPCs (5×10^4^ cells) diluted with EGM-2 basal medium were seeded into the cell culture inserts (pore size 8 μm, Corning) in the 24-well plate. Then, 750 μl of suspended ASCs (7.5×10^4^ cells) or the same volume of control medium (EGM-2 basal medium or EGM-2 MV complete medium) was added to the outer compartment of each well of the 24-well plate. After 8 h of coculture, the culture inserts were carefully removed and fixed in 4% paraformaldehyde. Non-migrated cells on the upper side of the membrane were removed with a cotton swab. The cells that had migrated were stained with crystal violet.

Invasion assays were used to analyze the proteolytic activity and capacity for migration of EPCs cocultured with ASCs. As described above, the EPCs were seeded into BioCoat matrigel-pretreated chambers (pore size 8 μm, Corning). ASCs were cocultured in the outer compartment. Twenty-four hours later, cells that had migrated to the bottom side of the membrane were fixed and stained with crystal violet. All the experiments were performed in triplicate. Cell counts for transmigration and invasion assays were obtained with Image-Pro Plus software and expressed as mean ± SEM.

### Seeding of an adipose tissue implant with ASCs and EPCs

To evaluate the capacity of ASCs to promote vascularization among EPCs in the setting of fat implantation, implanted adipose tissue was seeded with ASCs and EPCs. Donor fat was obtained from New Zealand white rabbits that had been anesthetized with pentobarbital (0.01 ml/g body weight at 5 mg/ml in 5% ethanol/PBS, intraperitoneally). New Zealand white rabbits were euthanized by intravenous injection of a lethal dose of phenobarbital (150 mg/kg) after surgery. The fat tissue obtained was cut into small cubes (approximately 5 mm×5 mm×3 mm) and kept in EGM-2 basal medium in a 24-well plate. A total of 4×10^5^ EPCs or EPCs+ASCs (ratio 1:1) were resuspended in 200 μl matrigel, then added to the fat tissue. The tissue and cells that had been embedded in gel were incubated for 30 minutes at 37°C to allow for solidification. Incisions of 4 mm in length were made on the backs of nude mice inhalation-anesthetized with isoflurane, and the gel-entrapped tissue was implanted subcutaneously. Thirty days after implantation, mice were sacrificed via gas inhalation (CO_2_). Fat tissue was harvested, weighed and subjected to immunohistochemistry to evaluate vascular network formation. To track EPCs *in vivo*, the EPCs were labeled with Edu with Click-iT Plus Edu kit (Thermo) according to the manufacturer’s instructions before coimplanted with adipose grafts. Then the fat tissue was harvested for Edu and CD31 immunostaining. All experiments were performed at Kunming Medical University in compliance with the university’s guidelines for the ethical treatment of experimental animals.

### Statistical analysis

All data are presented as means ± SEM. SPSS11 software was used for statistical analysis. The independent-sample t-test was used for comparisons between groups. Results with p≤0.05 were considered to be statistically significant.

## Results

### Characterization of EPCs and ASCs isolated from rabbit

**Fig 1:**
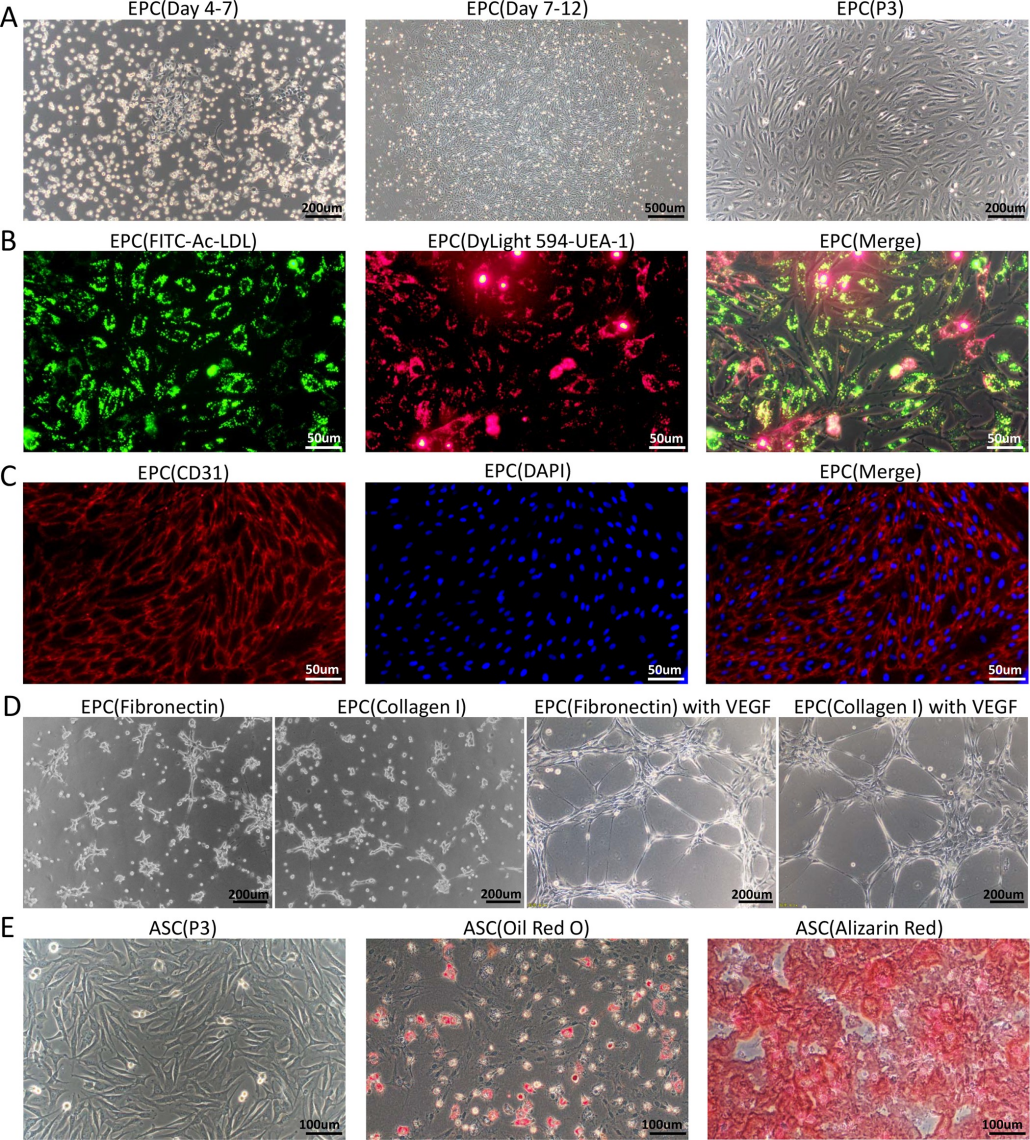
Isolation and characterization of rabbit EPCs and ASCs. (A)Rabbit peripheral blood mononuclear cells were seeded on plates pretreated with fibronectin and supplemented with EGM-2 complete medium. At 4 to 7 days, adherent cells with typical cobblestone-like morphology appeared. At 9–12 days, the EPCs expanded into colonies. Third-passage EPCs cultured in complete EGM-2 medium exhibited spindle-like morphology under inverted phase-contrast microscopy. (B) The functional characteristics of EPCs were assessed by testing their ability to take up FITC-Ac-LDL and bind to DyLight 594-UEA-1. The results were visualized with fluorescence microscopy. (C) Immunofluorescent staining revealed the expression of endothelial cell marker CD31 in EPCs. Cell nuclei are stained with DAPI. (D) Representative mages of the tube formations of EPCs isolated by fibronectin or collagen I coating methods, the EPCs were resuspended with EGM2 basal medium containing 50ng/ml VEGF or not, respectively. (F) Third-passage ASCs cultured in complete EGM-2 medium displayed fibroblast-like morphology. The multilineage differentiation potential of ASCs was assessed *in vitro*. Osteogenesis was assessed by staining with Alizarin Red to detect the formation of calcium-rich deposits. Adipogenesis was assessed by staining with Oil red O to detect lipid vacuoles in the cytoplasm.

Generally, 4–7 days after cultivation, sparse adherent cells began to appear in the primary EPC cultures. These adherent cells displayed a typical cobblestone-like morphology on fibronectin or collagen I-precoated plates. After 9–12 days, the rapidly proliferating EPCs formed several monolayer colonies with cobblestone appearance. These colonies were designated as passage 1 (P1). The P3 EPCs showed relatively homogeneous spindle-like morphology under inverted phase-contrast microscopy (Fig 1A). The P3 EPCs had functional characteristics of classical EPC cultures, such as the ability to take up Ac-LDL and bind to UEA-1 (Fig 1B). The P3 EPCs also stained positively for CD31, an endothelial cell marker (Fig 1C). Standard tube formation assay showed that the EPCs isolated by fibronectin or collagen I coated dish both expressed limited ability to form capillary structures on matrigel, while addition of VEGF promoted the tube formation by EPCs (Fig 1D).

ASCs were isolated from rabbit adipose tissue through collagenase digestion. Twenty-four hours after seeding of the stromal vascular fraction (VSF) pellet, most cells were found adhering to the surface of the culture plates. These cells were referred to as ASCs. As shown in Fig 1D, the P3 ASCs cultured in complete EGM-2 medium displayed a fibroblast-like morphology. The results of *in vitro* differentiation assays showed that ASCs exhibited adipogenic and osteogenic potential (Fig 1E).

### Molecular phenotypes of EPCs and ASCs

**Fig 2:**
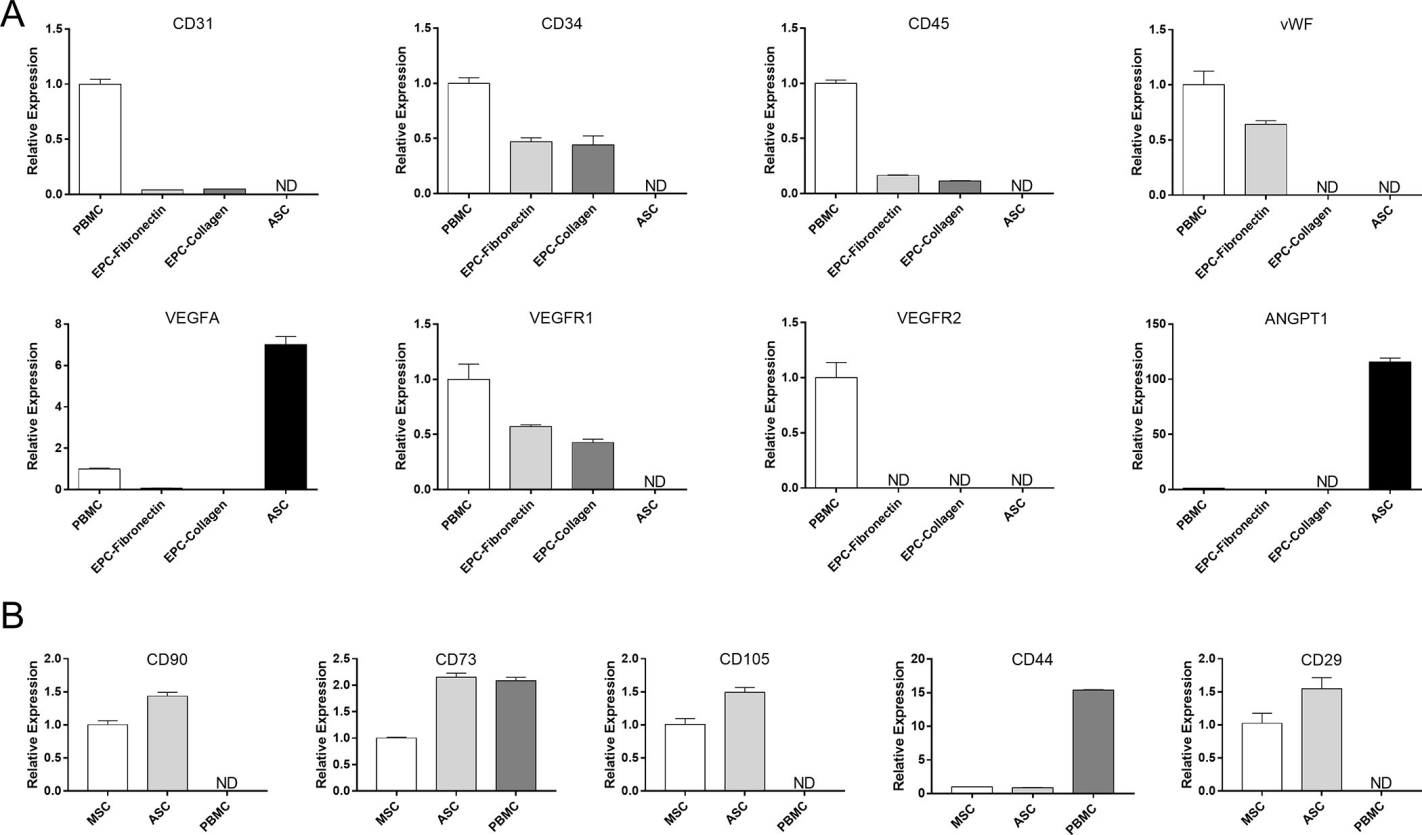
Molecular phenotyping of EPCs and ASCs isolated from rabbits. **(**A) The relative expression of surface markers and angiogenic factors in PBMCs, EPCs, and ASCs was evaluated with qPCR. (B) The transcription of key stemness markers in ASCs were evaluated with qPCR. The rabbit bone marrow mesenchymal stem cells (MSC) and rabbit PBMC were used as controls. Levels below the detection limit are depicted as ND. Data are expressed as mean ± standard errors of the mean (SEM).

As previously reported, both fibronectin and collagen I may be used as attachment factors in the isolation of EPCs [13, 14]. However, the molecular phenotypes of EPCs isolated with different approaches have not yet been compared. As shown in Fig 2A, the two types of EPCs shared a common pattern of marker gene expression. Both expressed similar levels of common endothelial markers (CD31 and VEGFR1) and hematopoietic stem cell marker (CD34), but not vWF. In contrast, ASCs expressed higher levels of angiogenic factors (VEGFA and angiopoietin 1) but did not express CD31, vWF, CD34, CD45, VEGFR1, or VEGFR2. The gene expression pattern of ASCs indicated angiogenic potential. In addition, the ASCs isolated in this study expressed all the common stemness markers of ASCs (CD90, CD105, CD73, CD34, CD44 and CD29) at a level comparable or higher to MSCs. In contrast, the CD90, CD105 and CD29 were rarely expressed in the PBMCs (Fig 2B). These results indicated that this rabbit ASCs were similar to the traditional MSCs or ASCs because of their multilineage differentiation potential (Fig 1E) and expression of stemness markers (Fig 2B).

### Tube formation in EPC and ASC cocultures in vitro

**Fig 3:**
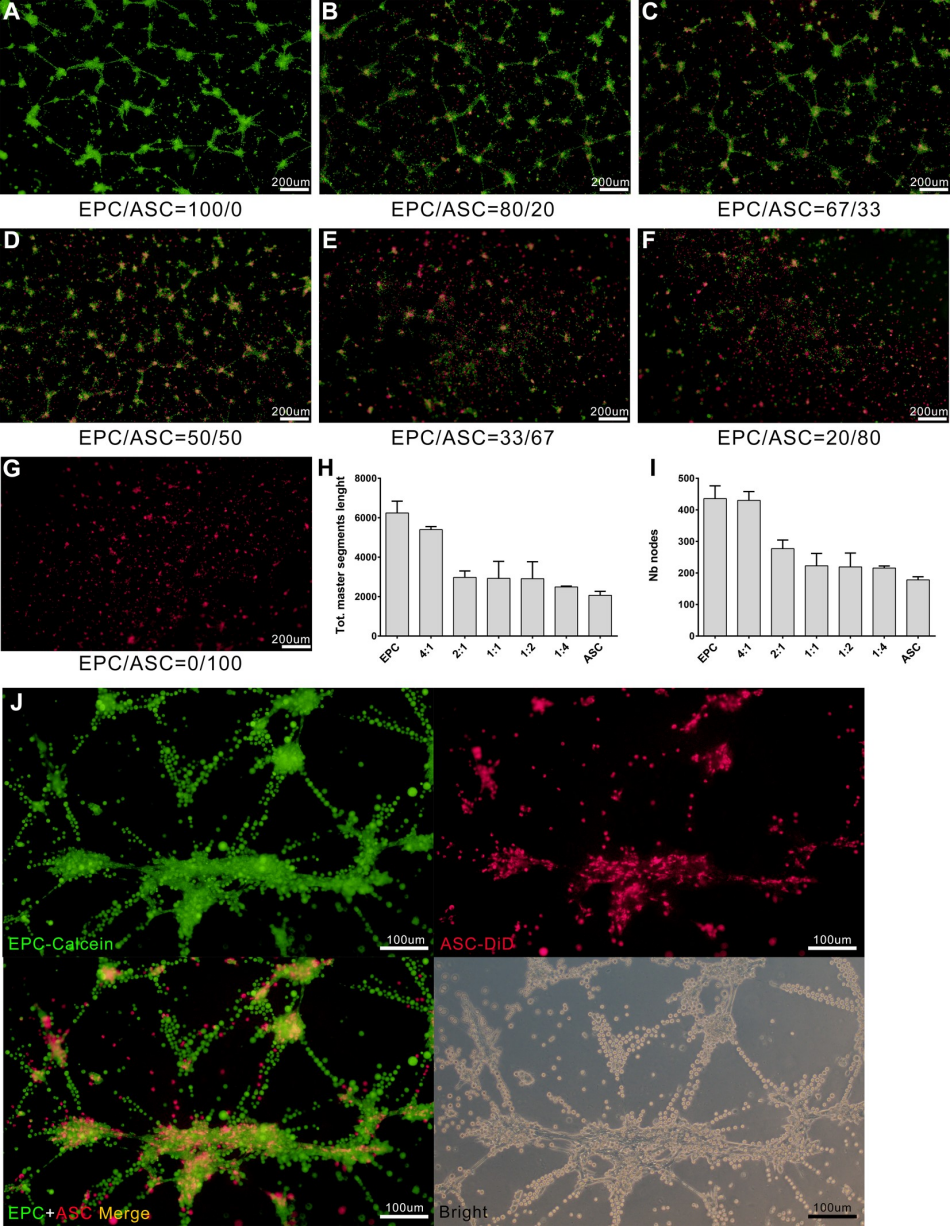
Tube formation in various ratios of cocultured EPCs and ASCs. (A-G) Cocultures prepared with various ratios of EPCs (labeled with Calcein-AM, Green green fluorescence) and ASCs (labeled with DiD, red fluorescence) were incubated for 4 h on matrigel-coated plates. Tube formation was analyzed using Image J Version 1.80. Each micrograph presents the results of 3 separate experiments. (H-I) Quantitative comparison of total master segment length (H) and number of formed branch nodes (I) in EPC-ASC cocultures. Data are presented as mean ± SEM. *, *p* < 0.05. (J) Representative images of tube formation of EPC and ASC cocultures (at ratio of 1:1).

ASCs have previously been described as mutipotent cells with pericytic properties that stabilize endothelial network formation *in vitro* and *in vivo*. Here we tested whether ASCs could promote the formation of vessel-like structures through direct cooperation with EPCs to coassemble vessels in a 2D *in vitr*o model. As shown in Fig 3, EPC-ASC cocultures of varying ratios on matrigel did not generate well-defined capillary-like structures. Primitive networks composed of interrupted rings and agglomerates were found in cocultures containing a high proportion of EPCs (Fig 3A–3G). Quantitative analysis was performed to determine potential tube length and to determine the number of branch nodes. The results suggested that ASCs did not promote in tube formation by EPCs (Fig 3H–3I). However, by labeling cells with different fluorochrome, ASCs was found to migrate and integrated into the agglomerates formed by EPCs. Meanwhile, most of the capillary-like structures were formed only by EPCs (Fig 3J). These results indicated that ASCs can participate in the tube formation of EPCs although it cannot form canonical capillary structures like vascular endothelial cells do. Therefore, the tube formation of cocultures significantly decreased as the ratio of ASCs in cocultures increased.

### ASCs promote the angiogenesis of EPC through paracrine action

**Fig 4:**
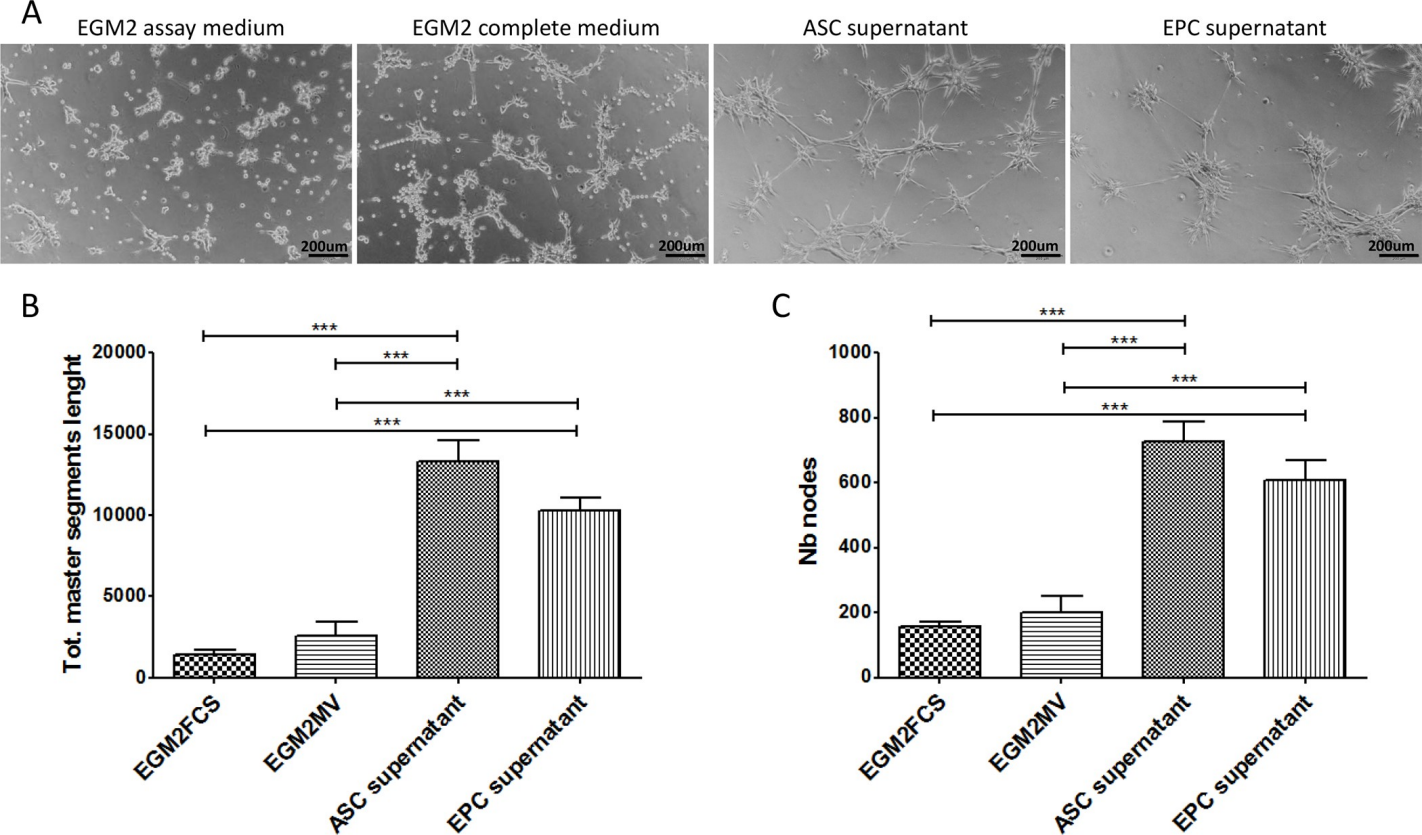
Supernatant from ASCs cultures promotes EPCs tube formation *in vitro*. (A) Representative images of EPCs tube formation on matrigel in the presence of supernatant from ASCs or EPCs cultured for 48 hours. Fresh EGM2 assay medium was used as a negative control, and complete EGM2 medium was used as a positive control. Higher-magnifications views are presented (B-C). Total master segment length (B) and number of branch nodes (C) were calculated from 6 random fields in images of three wells in series. Data are presented as mean ± SEM. ***, *p* < 0.001.

**Fig 5:**
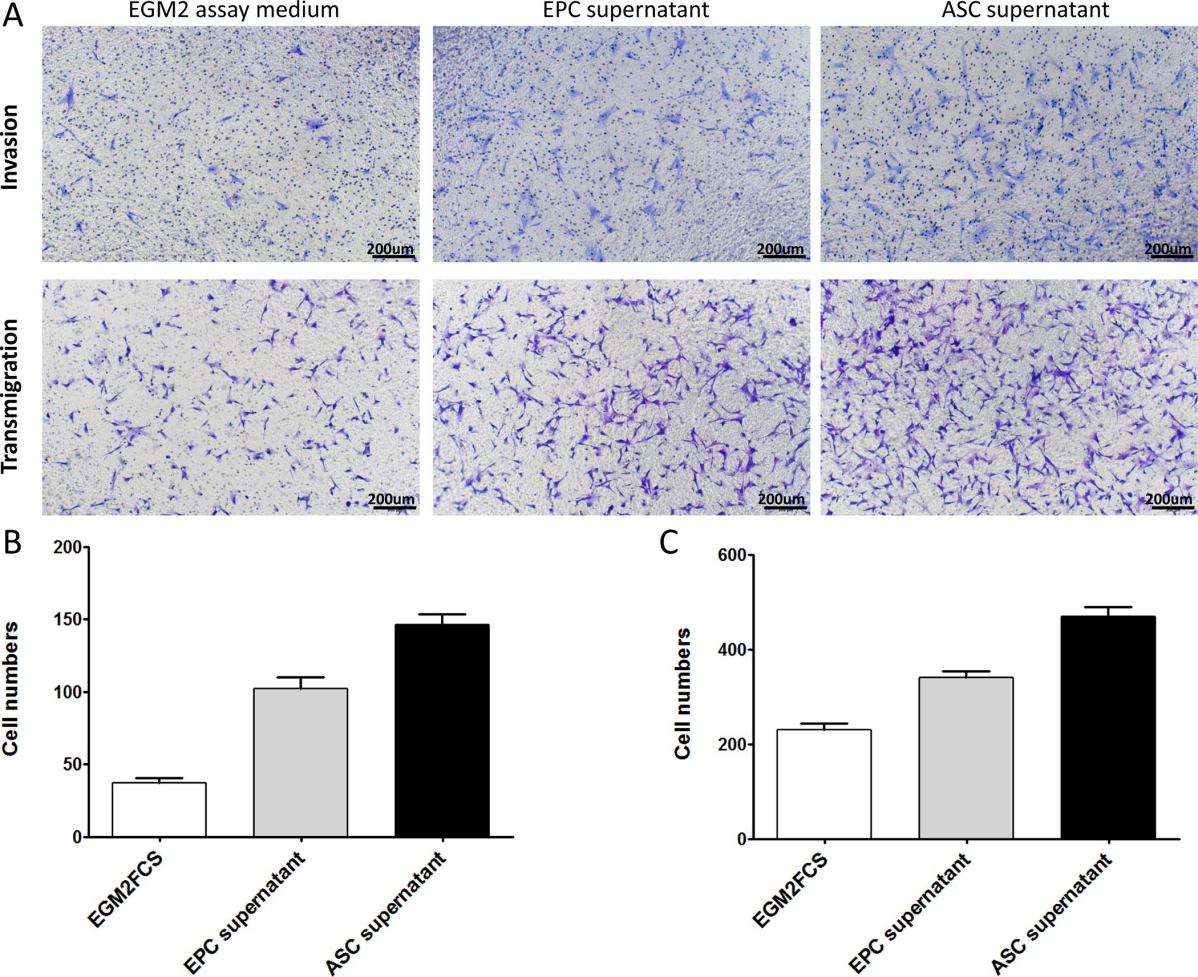
Supernatant from ASCs cultures promotes EPCs invasion and migration *in vitro*. (A)Representative images of EPCs invasion and migration in the presence of EPCs supernatant, ASCs supernatant, or fresh EGM2 assay medium, respectively. Quantitative analysis of EPC invasion (B) and migration (C). Data are expressed as mean ± SEM. *, *P* < 0.01.

As noted above, ASCs persistently expressed high levels of VEGFA and ANGPT1 during culture with EGM2 medium. ASCs may therefore promote the angiogenesis of EPCs through paracrine signaling. To test this, EPCs were seeded on matrigel in the presence of fresh control medium or supernatant from ASC or EPC cells cultured for 48 hours. Tube formation was analyzed after incubation for 4 hours. As shown in Fig 4A, EPCs suspended in fresh EGM2 basal medium or EGM2 complete medium (containing growth factors optimized for endothelial cells) showed limited tube formation. This finding supports the results presented in Fig 3. EPCs suspended in ASC or EPC culture medium generated typical vessel-like network. Quantitative analysis also confirmed significantly more proangiogenic activity in cultures with ASC and EPC medium, compared with controls (Fig 4B). Further analysis indicated that, after culture for 48 hours, supernatant from ASC and EPC culture promoted the migration and invasion of EPCs seeded in transwell inserts (Fig 5A). These results indicate that ASCs secrete a variety of growth factors that promote the angiogenesis of EPCs and/or the differentiation of EPCs into endothelial cells. EPCs may also have the ability to promote the angiogenesis via an autocrine mechanism.

**Fig 6:**
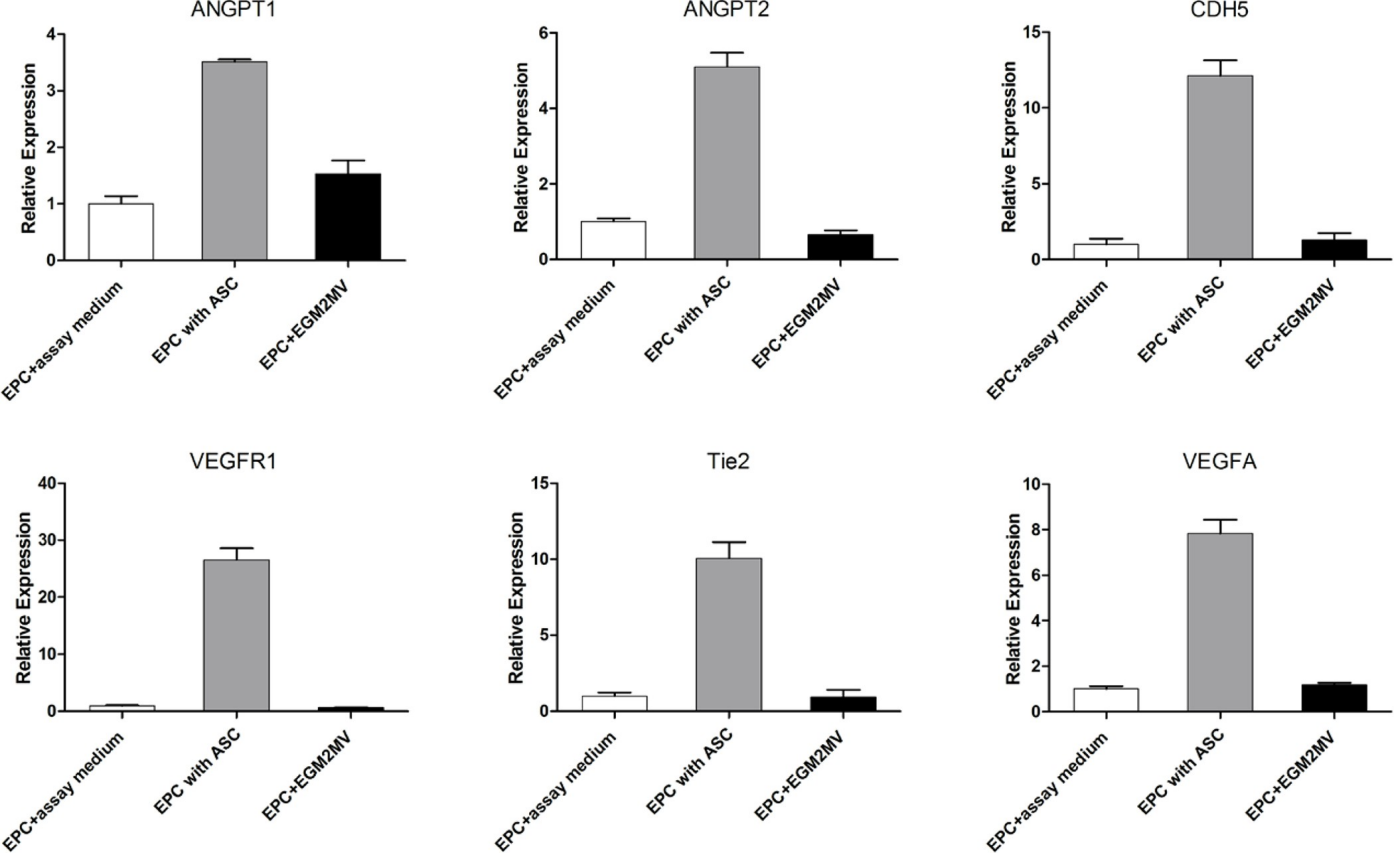
Coculture with ASCs promote the expression of angiogenesis markers in EPCs. EPCs were cocultured with ASCs or an equivalent volume of empty EGM2 assay medium or complete EGM2 medium as controls. After coculture for 24 hours, EPCs were harvested for qPCR detection of the expression of angiogenesis markers including VEGFA, ANGPT1, ANGPT2, CDH5, VEGFR1, and Tie2. Levels below the threshold for detection are depicted as ND. Data are expressed as mean ± SEM. *, *P* < 0.01.

To verify the ability of ASCs to regulate angiogenesis-relate gene expression in EPCs, an *in vitro* coculture system with transwell chambers was established. The EPCs were cocultured with ASCs or empty control medium for 24 hours, then subjected to qPCR to measure the transcription of a panel of six potential angiogenesis markers. As shown in Fig 6, coculture with ASCs significantly increased the transcription of angiogenic cytokines (VEGFA, angiopoietin-1, and angiopoietin-2), endothelial cell receptor tyrosine kinases (VEGFR1, TIE2) and endothelial cell adhesion molecules (CDH5) in EPCs, compared with controls. Notably, levels of VEGFR2, the main mediator of VEGF-induced endothelial proliferation and tubular morphogenesis, remained below the threshold required for detection. These findings suggest that binding to the Tie2 receptor by angiopoietins (ANGPT1 and ANGPT2) may play a more prominent role than VEGF in EPC angiogenesis. Moreover, the upregulation of endothelial cell adhesion molecules in EPCs suggests differentiation into a mature endothelial form.

### Co-transplantation of ASCs and EPCs promotes angiogenesis of adipose tissue grafts *in vivo*

**Fig 7:**
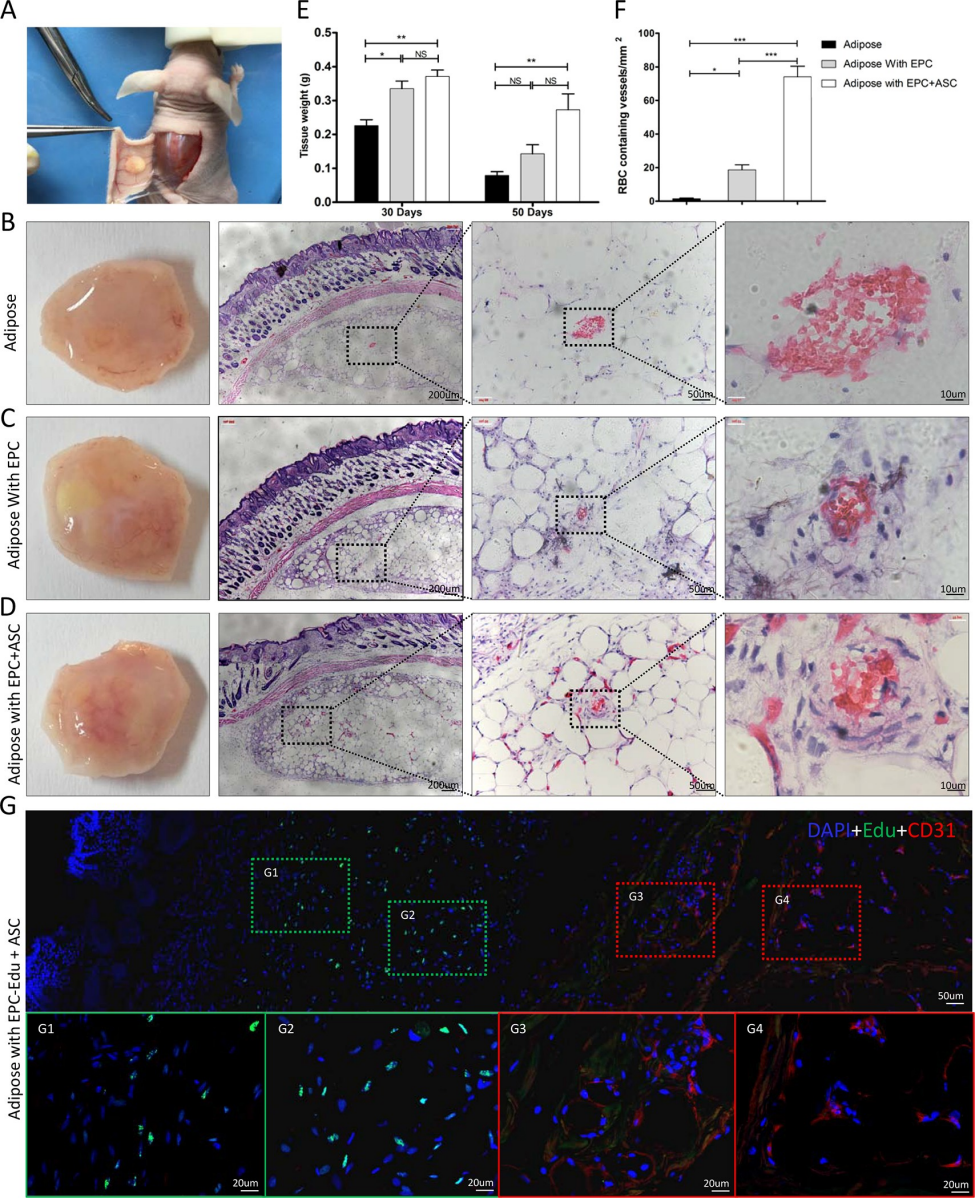
Coimplantation of ASCs with EPCs increases vascular density in adipose grafts. (A) Photograph illustrating the subcutaneous transplantation of adipose tissue in nude mice. In each group, the fat implants were harvested at 30 days postimplatation and stained with HE. (B-D) Representative photographs and images of HE-stained sections of implanted adipose tissue (B), implanted adipose tissue containing EPCs (C), and implants containing a combination of ASCs and EPCs (D). Higher-magnification images of the boxed areas are shown on the right. (E) In another experiment, the fat tissue in all three groups were dissected and weighted. Data are expressed as the mean ± SEM. *, *P* < 0.05; **, *P* < 0.01; NS = not significant. (F) The density of RBC-filled vessels in the implants of each group was analyzed. Data are expressed as mean ± SEM. *, *P* < 0.05; **, *P* < 0.01. (G) Represent images of immunofluorescence staining of Edu labeled EPC and CD31 positive vessels in the adipose implants. The higher magnifications of the boxed areas are shown on the lower panel. CD31 expression was stained in red, Edu labeled EPCs was stained in green, nuclei were stained in blue with DAPI.

ASC and EPC are reported to promote the survival of adipose tissue grafts by increasing vascularization. In considering the proangiogenic effects of ASCs on EPCs *in vitro*, we speculated that the combination of ASCs and EPCs might be more effective in promoting the vascularization of adipose grafts than use of either cell type alone. To test this, intact adipose blocks were transplanted subcutaneously into nude mice. Tissue blocks were embedded in matrigel seeded with EPCs, a combination of ASCs and EPCs (1:1 ratio), or no cells. As shown in Fig 7B–7D, the appearance of adipose grafts dissected at 1-month post-implantation differed among groups. Only the grafts seeded with a combination of EPCs and ASCs were found to have adequate blood supply, with thick blood vessels extended into the grafts. In contrast, grafts containing no cells or EPCs alone were consistently more whitish in color, with thin vascular structures only on the graft surface. The retention rate of adipose graft was evaluated by weighing method. As shown in Fig 7E, the weight of the fat tissues in all three groups significantly declined at 50 days post-implantation. However, the retention rate of adipose graft in EPCs and ASCs coimplantation group was significantly higher than other groups. To evaluate the vascular density in explanted adipose grafts, histological sections of each group were stained with hematoxylin/eosin (H&E), which binds to vessels with large lumens containing blood elements. As shown in Fig 7B–7D, typical vascular structures with multilayered vascular walls and erythrocytes were commonly seen in grafts containing EPCs or the combination of ASCs and EPCs, but not in grafts that were not seeded with ASCs or EPCs. Quantitative analysis revealed that the vascular density in grafts containing ASCs and EPCs was significantly higher than that of grafts containing only EPCs (Fig 7F). To evaluate the endogenous vessels and EPC derived vessels in the fat graft, EPCs were labeled with Edu before coimplantation with ASCs. Immunofluorescence staining of Edu labeled EPC and CD31 positive vessels indicated that most EPCs stayed around the fat tissue block, and vessels in the fat transplant containing no Edu-positive cells. Generally speaking, these results indicate that ASCs may cooperate with EPCs to promote vascularization in transplanted adipose tissue. Meanwhile, EPCs may promote the angiogenesis by paracrine regulation of endogenous endothelial cells rather than directly participate in the formation of new blood vessels.

## Discussion

The low survival rate and absorption of transplanted fat have posed problems for clinicians. The uncertainty of the rate of absorption in patients who have undergone fat transplantation means that plastic surgeons may administer too many injections or inject excessive amounts of fat [15–17]. Low rates of fat survival and absorption are generally associated with poor vascularization after transplantation [18].

Studies have shown that ASCs promote angiogenesis in the context of ischemia. ASCs can differentiate into other types of cells, such as endothelial cells, vascular smooth muscle cells, and cardiomyocytes [19–21]. They also secrete a variety of functional growth factors and cytokines that promote angiogenesis (angiogenin, VEGF, HGF, bFGF, B-NGF) [22, 23]. The results of *in vitro* experiments have shown that these cytokines can promote the survival of endothelial cells under hypoxic conditions [24]. Numerous researchers have sought to improve the survival rate of transplanted fat by using ASCs to promote the angiogenesis of adipose tissue. The results of many clinical trials and basic science studies on angiogenesis, immunomodulation, and tissue regeneration have shown that ASC paracrine signaling facilitates the regeneration of adipose tissue [25, 26].

EPCs not only form blood vessels *in vitro* but also function as the building blocks of blood vessels *in vivo*. Many studies have shown that transplanted EPCs can migrate to the ischemic or injured site [27–29]. EPCs that have migrated secrete cytokines or differentiate into endothelial cells to participate in the construction of new blood vessels [27–29]. The new blood vessels formed can be integrated into the blood vessels of the host and then participate in nutrient transport. When vascular endothelial damage occurs in the body, EPCs located in the bone marrow can be mobilized to the injured site [30]. The mobilized EPCs secrete a variety of angiogenic factors and differentiate into vascular endothelial cells, which promote vascular repair and neovascularization [30]. An *in vitro* three-dimensional (3D) network model was established to study the role of EPCs as a source of angiogenic factors. The results confirmed that EPCs can secrete VEGF and participate in vascular repair and neovascularization.

In 2007, Yoshimura et al [31] reported that the use of stromal vascular fraction (SVF)-rich fatty stem cell-assisted fat transplantation for breast augmentation surgery achieved good results. In 2009, autologous cell-assisted lipotransfer (CAL) technology was proposed [32]. CAL technology mixes autologous ASCs with the adipocytes to be used for transplant [28]. Since the introduction of CAL technology, scholars have conducted numerous basic and clinical research studies on the mechanisms underlying the survival of transplanted fat. ASCs appear to play a major role. ASCs promote vascular regeneration in transplanted fat, including VEGF. ASCs promote fat regeneration and blood vessel formation by differentiating into adipocytes and vascular endothelial cells. However, further research is needed. Paracrine signaling among ASCs may also aid in the treatment of ischemic disease [33–37]. Previously published studies have demonstrated the therapeutic effect of ASCs. Two mechanisms are known to underlie this effect: multiple differentiation and paracrine signaling [38, 39]. Zhao et al. [40] showed that the role of ASCs in promoting angiogenesis is more closely connected to paracrine signaling, rather than differentiation. Our research results also confirm this view.

The purpose of this study was to explore the relationship between ASCs and EPCs during angiogenesis. Through tube formation assay of EPCs-ASCs cocultures, we found that the ASCs can participate in the tube formation of EPCs although the tube formation of cocultures dicreased with the increasing of ASCs (Fig 3). This result did not conflict with Strassburg’ study [41]. In the design of Strassburg’ experiment, the cell numbers of EPCs remained constant among different groups and the relatively long term culturing may contribute to amplify the angiogenic effects mediated by ASC through secretion of multiple angiogenic growth factors. Both studies proved that the soluble factors secreted by ASCs could promote the tube formation by EPC, except that Strassburg et al. suggested the cell–cell contact between ASCs and EPCs also promote capillary-like structure formation by EPCs. Similarly, Yuan et al. [42] found that, during the early stage of fat transplantation, ASCs did not differentiate into endothelial cells, pericytes, or smooth muscle cells to form new blood vessels. Perhaps numerous angiogenic growth factors were released to induce the formation of new blood vessels in the area surrounding the graft. To investigate whether ASCs could promote the angiogenesis of EPCs through a paracrine effect, we extracted the supernatant of ASCs cultured for 48 h. The results showed that ASCs supernatant was more effective than control or EPCs supernatant in promoting blood vessel formation by EPCs (Fig 4). These results indicate that ASCs promote EPC tube formation by secreting growth factors. We next sought to investigate how ASCs supernatant promoted angiogenesis among EPCs. To that end, we conducted EPCs invasion and migration experiments. The results of these experiments showed that the supernatant from EPCs culture as well as the supernatant from ASCs culture promoted the migration and invasion of EPCs. However, the effect of the ASCs supernatant on EPC migration and invasion was greater than that of the EPCs supernatant (Fig 5). These findings confirmed that ASCs also exert a chemotactic effect on EPCs by secreting growth factors. Under hypoxic conditions, ASCs may recruit more EPCs through chemotaxis, promoting the formation of blood vessels and decreasing tissue hypoxia. We measured gene expression in EPCs incubated with ASC-conditioned medium. The results showed increased levels of ANGPT1, ANGPT2, CDH5, FLt1, Tie2, and VEGF (Fig 6). The expression of these factors may allow EPCs to repair vascular damage by forming new blood vessels to decrease tissue ischemia. These findings support the hypothesis that ASCs recruit EPCs through chemotaxis. ASCs appear to communicate in a paracrine fashion through the secretion of cytokines that promote the repair of blood vessels and the generation of new blood vessels through the upregulation of angiogenic factors.

At last, we evaluate the effects of ASCs and EPCs on the survival of transplanted fat cells in nude mice. We performed tissue staining to visualize angiogenesis of the transplanted tissue. One month after implantation, the results of immunohistochemical analysis showed that blood vessels had grown into adipose tissue in the experimental group but not in the control group (Fig 7). Angiogenesis is an intricate multi-step event, and VEGF and ANGPT1 play important roles in this process. Angiogenesis requires endothelial cells to differentiate and proliferate. Remodeling leads to the formation of new buds, which mature into blood vessels [43–45]. These processes depend on the interaction between EPCs and pre-existing blood vessels. The early angiogenesis observed in adipose tissue grafts seeded with ASCs and EPCs is thought to be mediated by various angiogenic growth factors, which initiate new blood vessel formation and vascular remodeling. ASCs may communicate in a paracrine fashion by secreting multiple growth factors to promotes EPC-mediated blood vessel formation.

This study had some limitations. The molecular mechanism of the beneficial role of ASCs-EPCs in fat transplantation requires further elucidation. The results of this animal model should be verified in clinical trials. These limitations should be addressed in subsequent studies.

In summary, our results show that the transplantation of fat in combination with ASCs and EPCs can promote the survival of grafts. ASCs can regulate the expression of angiogenic factors in EPCs. These angiogenic factors act as paracrine signals to promote neonatal angiogenesis, suggesting that the use of ASCs and EPCs in combination with fat transplantation can promote the survival of grafts and the mechanism of neovascularization.

## Conclusions

This study find that early EPC have limited capacity to secrete angiogenic factors, which facilitates tube vessel formation. In contrast, autogenous ASC express high levels of VEGFα and angiopoietin-1. The results of *in vitro* studies showed that ASC-conditioned medium promotes the capacity of EPC for invasion, migration, and tube generation. The results obtained with our co-culture model further demonstrated that ASCs promote the expression of a series of angiogenesis marker genes in EPCs, indicating that ASCs may promote the maturation or angiogenic potency of EPCs by secreting VEGF-α and angiopoietin-1. The results obtained in experiments involving the *in vivo* transplantation of fat also demonstrated that co-implantation with ASCs and EPCs, compared with the implantation of EPCs alone, increased the vascularization of adipose grafts. The findings presented above not only demonstrate the superiority of co-implanted ASCs and EPCs in the context of fat transplantation but also suggest a potential mechanism.

## Supporting information

S1 Dataset (ZIP)

S2 Dataset (ZIP)

S3 Dataset (ZIP)

S4 Dataset (ZIP)

S5 Dataset (ZIP)

S6 Dataset (ZIP)

S7 Dataset (ZIP)

## Abbreviations

ASCs: adipose-derived stromal/stem cell
EPCs: endothelial progenitor cell
VEGF: vascular endothelial growth factor
HGF: hepatocyte growth factor
PB-EPC: Peripheral blood adipose-derived stromal/stem cell
PBMC: Peripheral blood mononuclear cell
Ac-LDL: acetylated low-density lipoprotein
UEA-1: Ulex europaeus agglutinin-1
VEGFR2: vascular endothelial growth factor receptor 2
PBS: phosphate-buffered saline
BSA: bovine serum albumin
RBC: red blood cell
qRT-PCR: Quantitative real-time PCR
ANGPT1: Angiopoietin-1
MSC: mesenchymal stem cell
GAPDH: glyceraldehyde-3-phosphate dehydrogenase
VSF: vascular fraction
H&E: hematoxylin/eosin
