# Supplementary figures and images for: Effects of adipose-derived stromal cells and endothelial progenitor cells on adipose transplant survival and angiogenesis
Source: PLoS One. 2022 Jan 13;17(1):e0261498. doi: 10.1371/journal.pone.0261498 (PMC8758088; doi:10.1371/journal.pone.0261498)

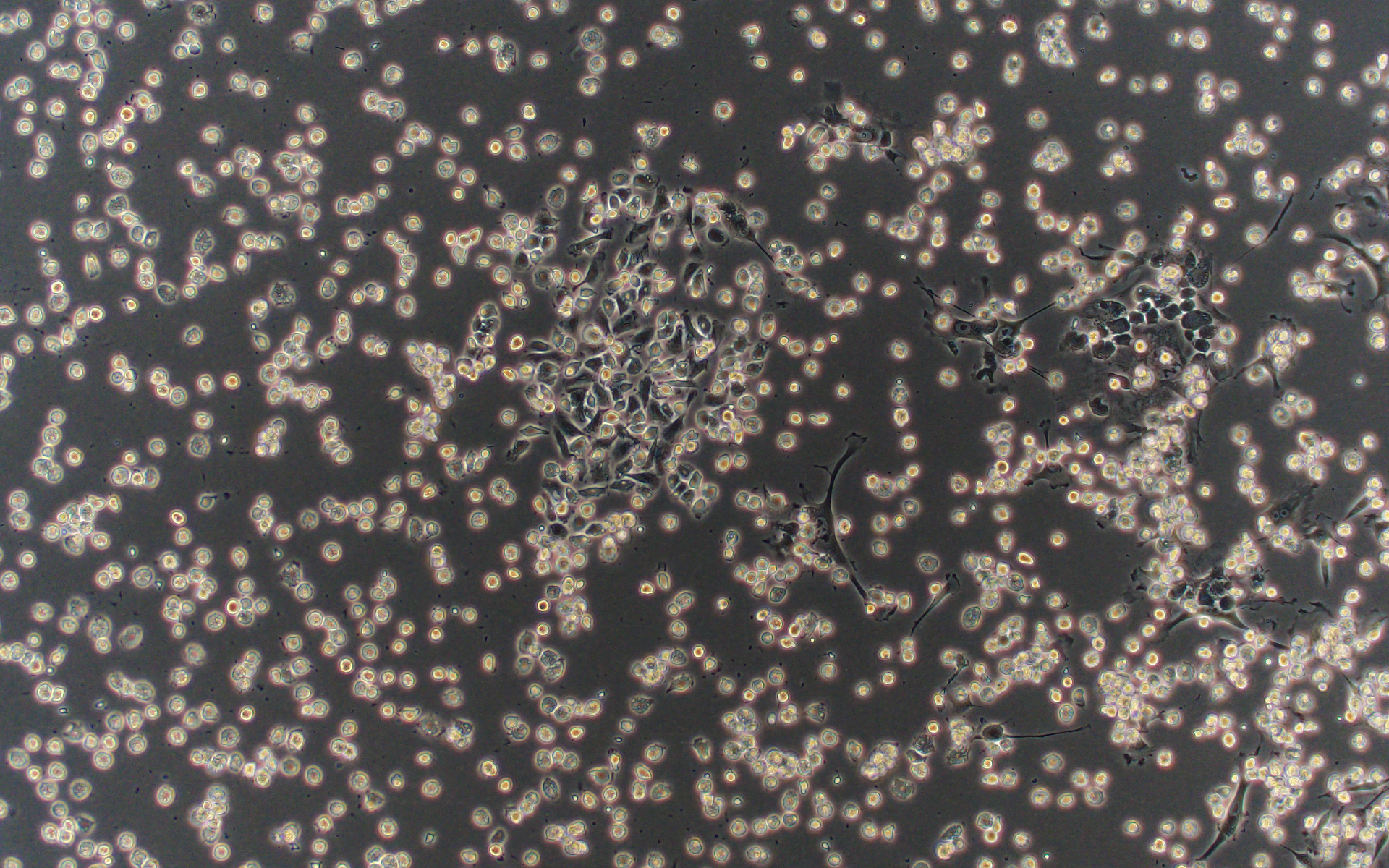

Supplement: S1 Dataset — (ZIP) [file pone.0261498.s001.zip › Fig 1/Fig 1 A1.tif]

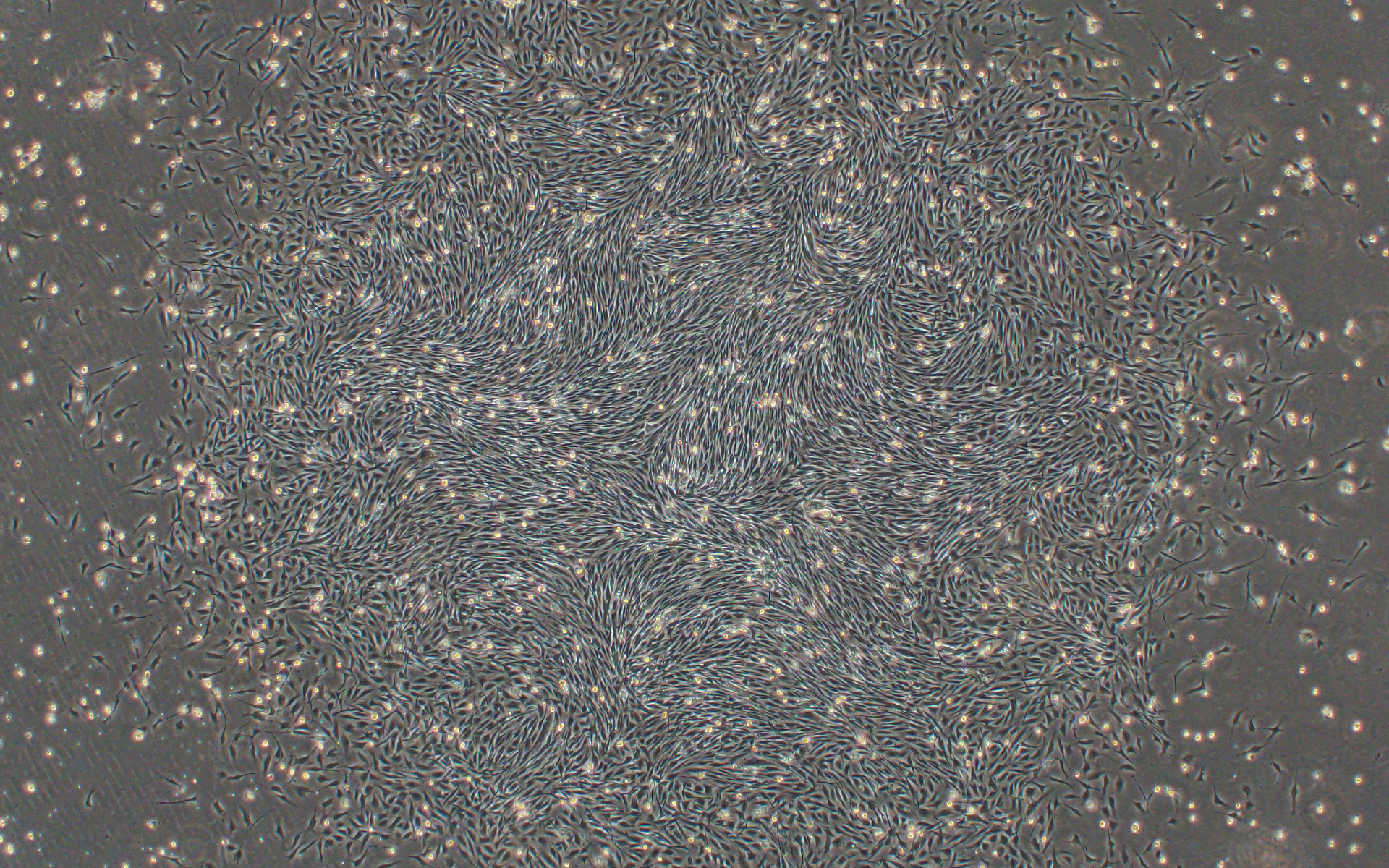

Supplement: S1 Dataset — (ZIP) [file pone.0261498.s001.zip › Fig 1/Fig 1 A2.tif]

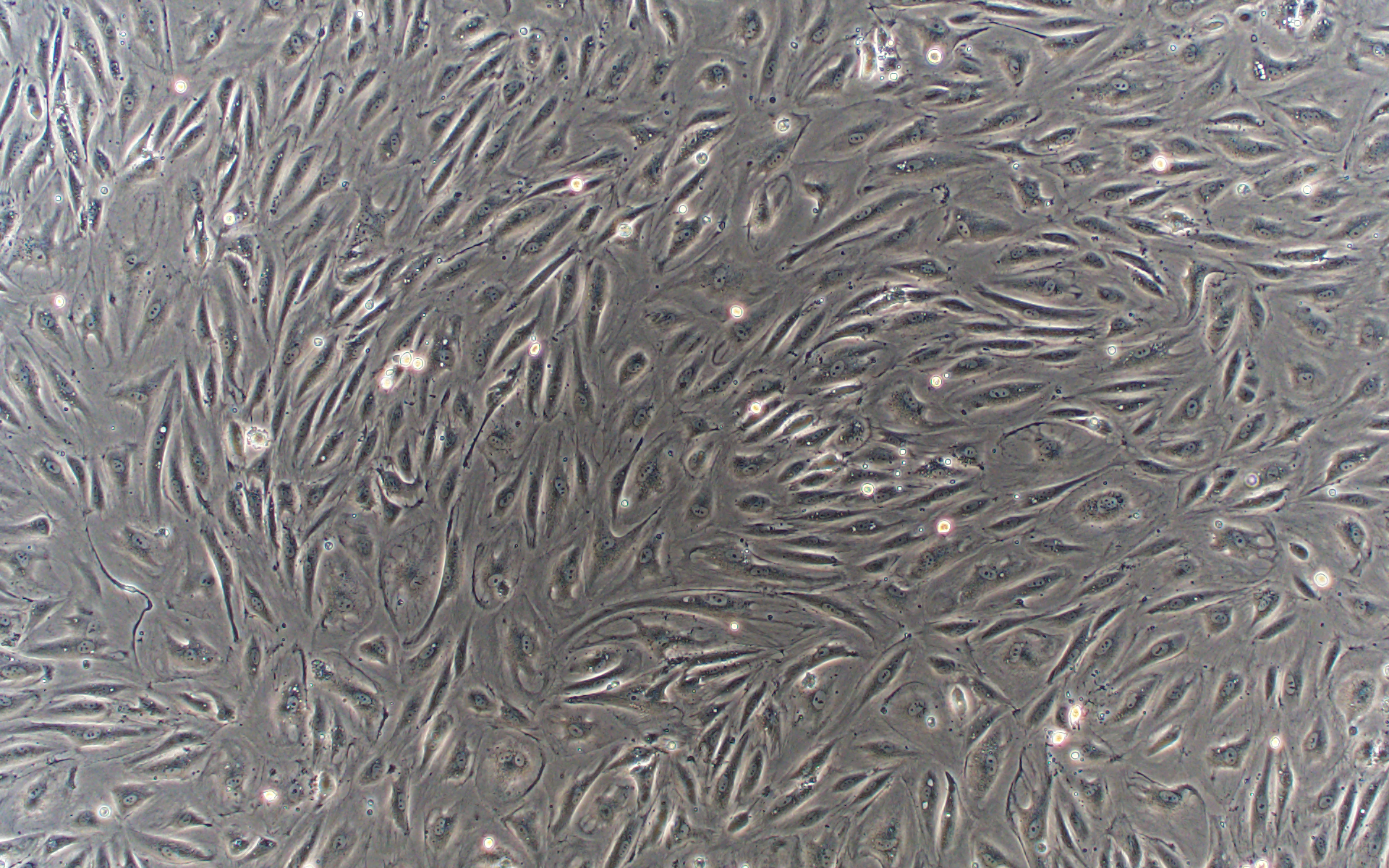

Supplement: S1 Dataset — (ZIP) [file pone.0261498.s001.zip › Fig 1/Fig 1 A3.tif]

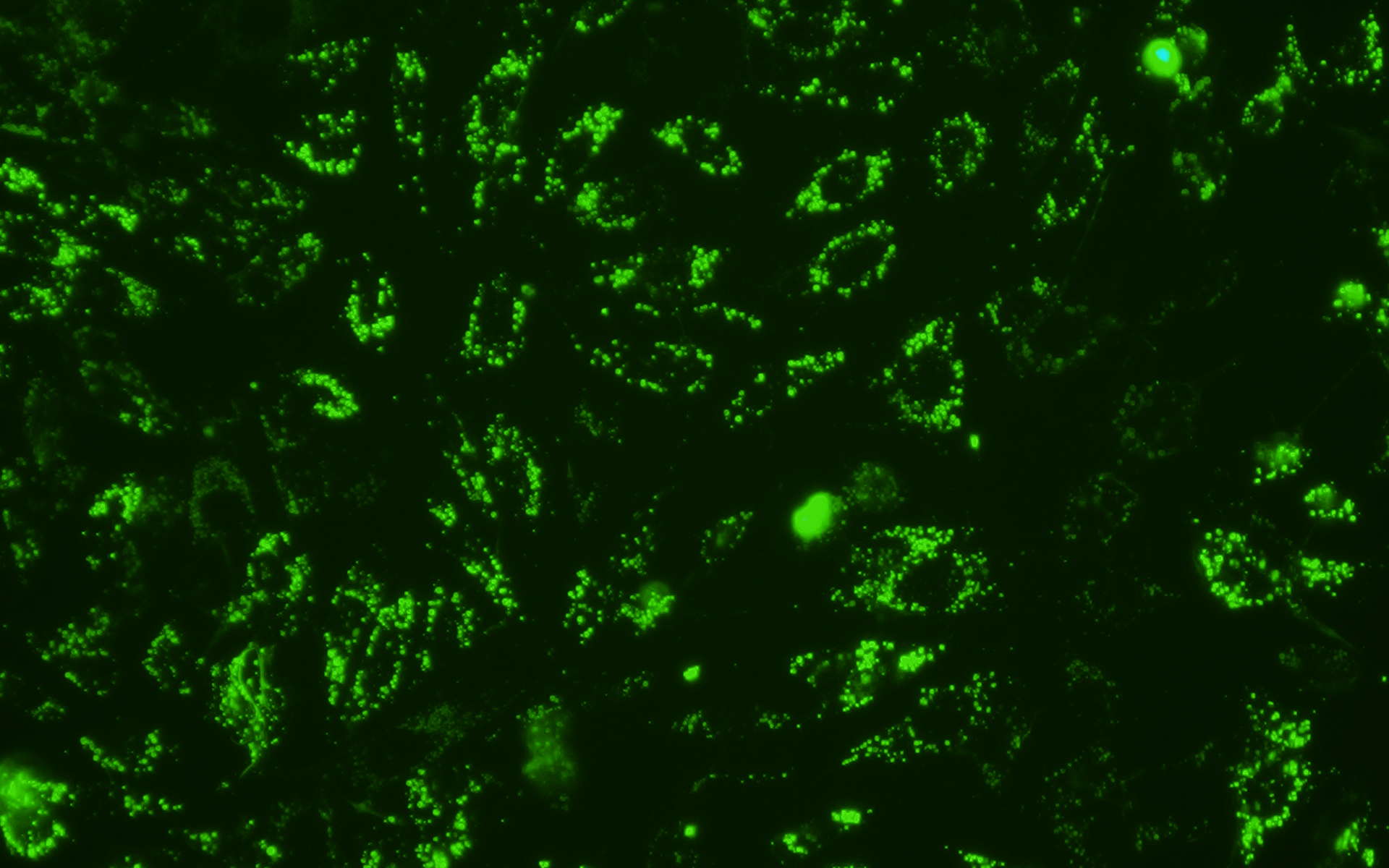

Supplement: S1 Dataset — (ZIP) [file pone.0261498.s001.zip › Fig 1/Fig 1 B1.tif]

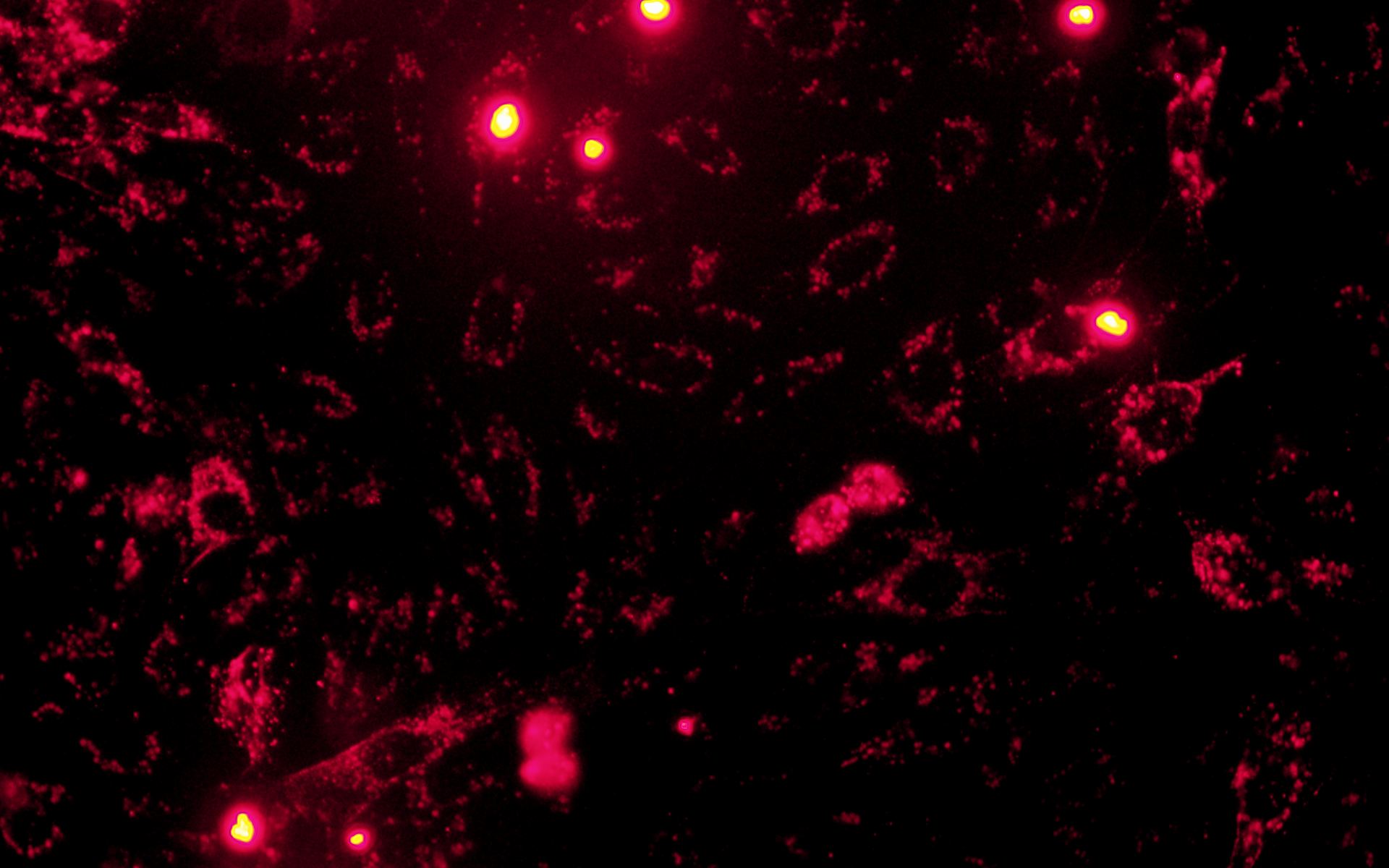

Supplement: S1 Dataset — (ZIP) [file pone.0261498.s001.zip › Fig 1/Fig 1 B2.tif]

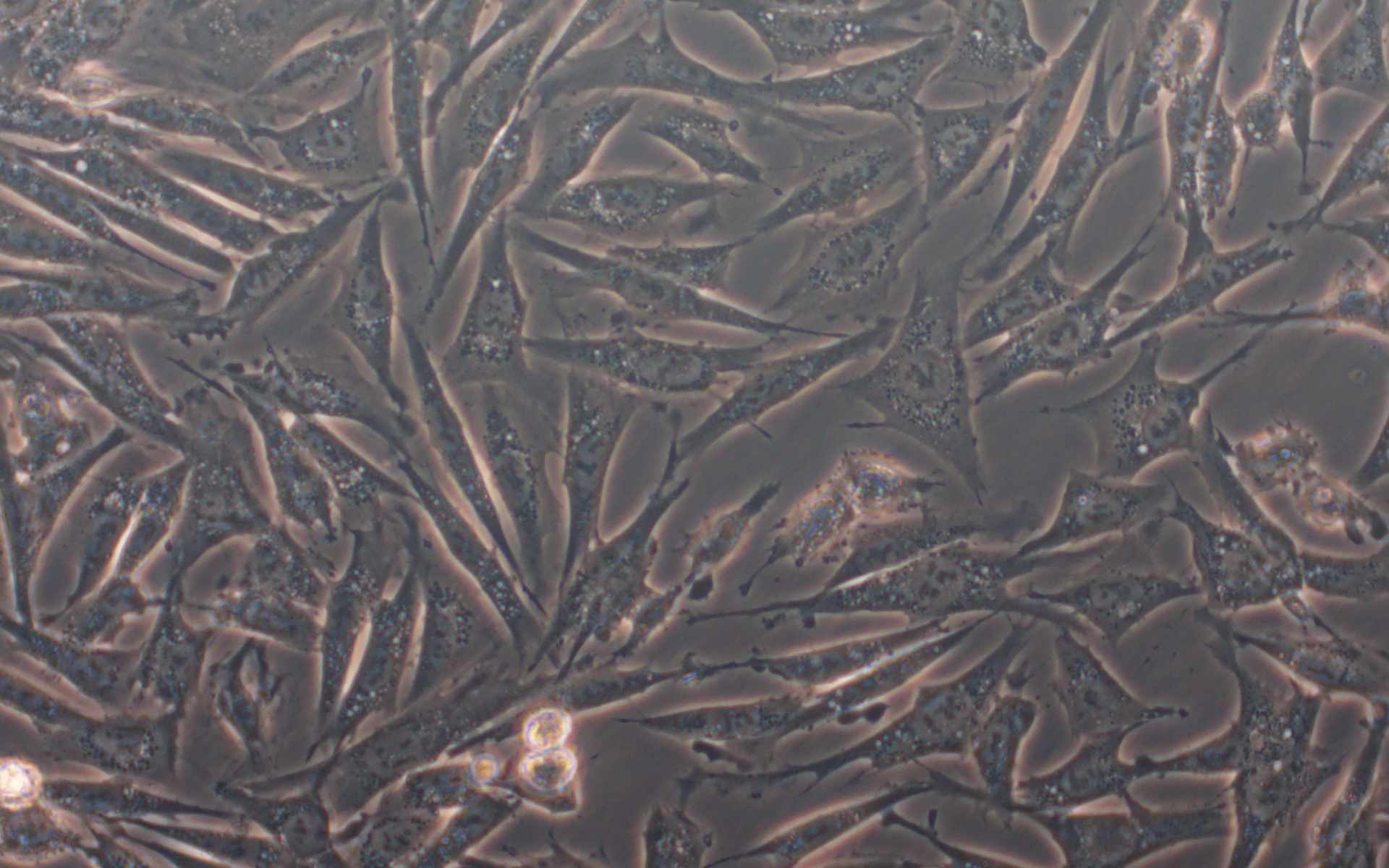

Supplement: S1 Dataset — (ZIP) [file pone.0261498.s001.zip › Fig 1/Fig 1 B3.tif]

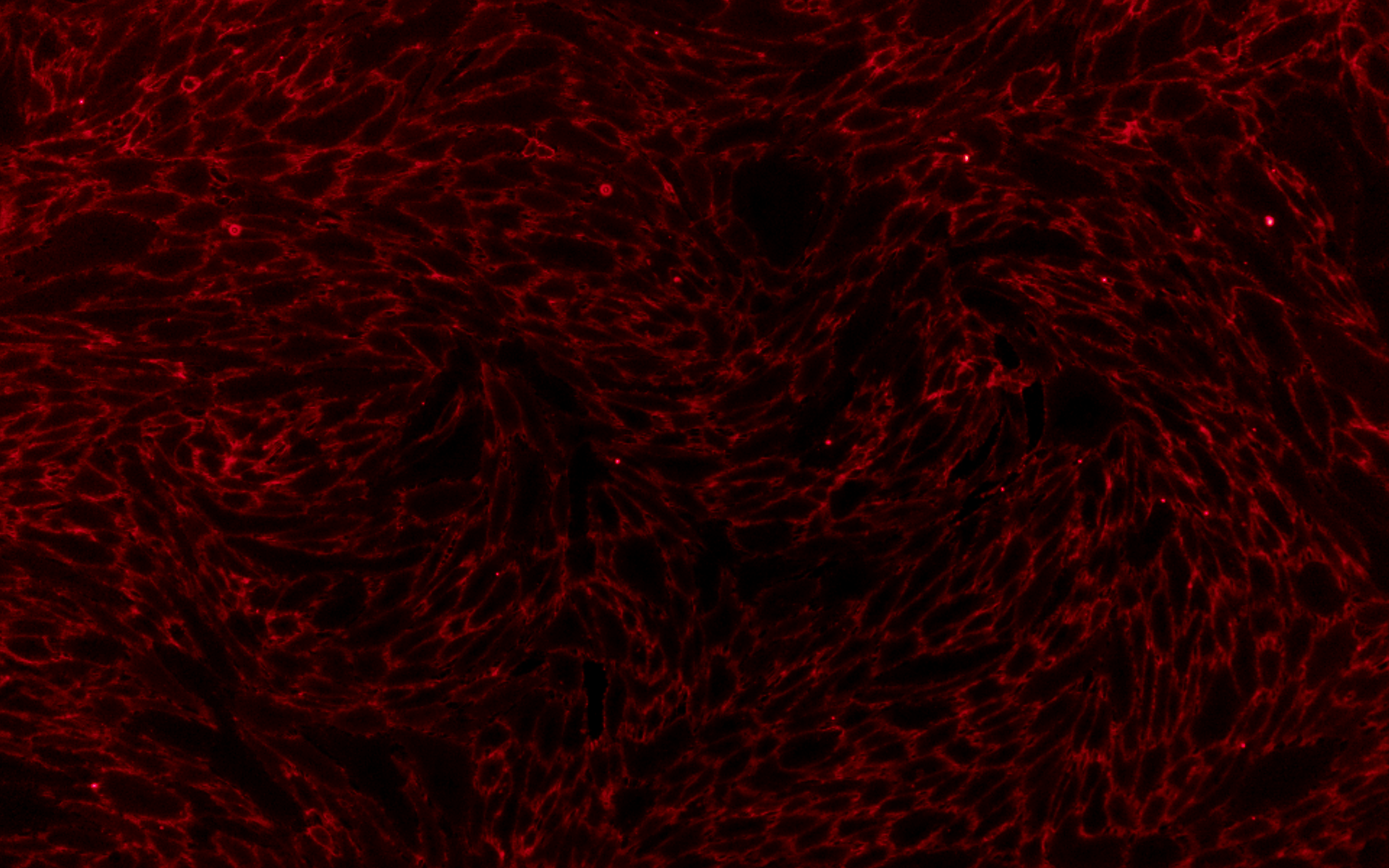

Supplement: S1 Dataset — (ZIP) [file pone.0261498.s001.zip › Fig 1/Fig 1 C1.tif]

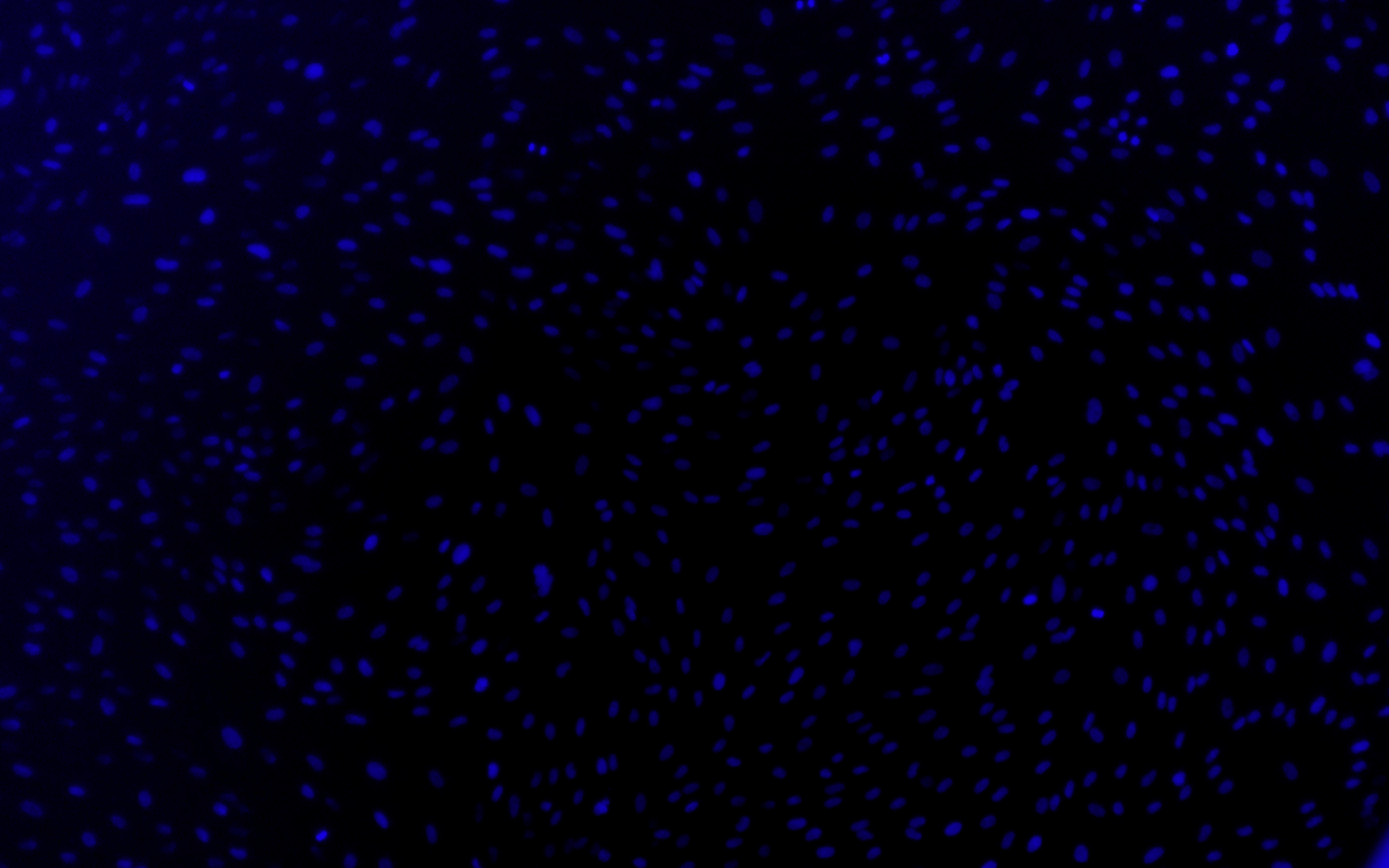

Supplement: S1 Dataset — (ZIP) [file pone.0261498.s001.zip › Fig 1/Fig 1 C2.tif]

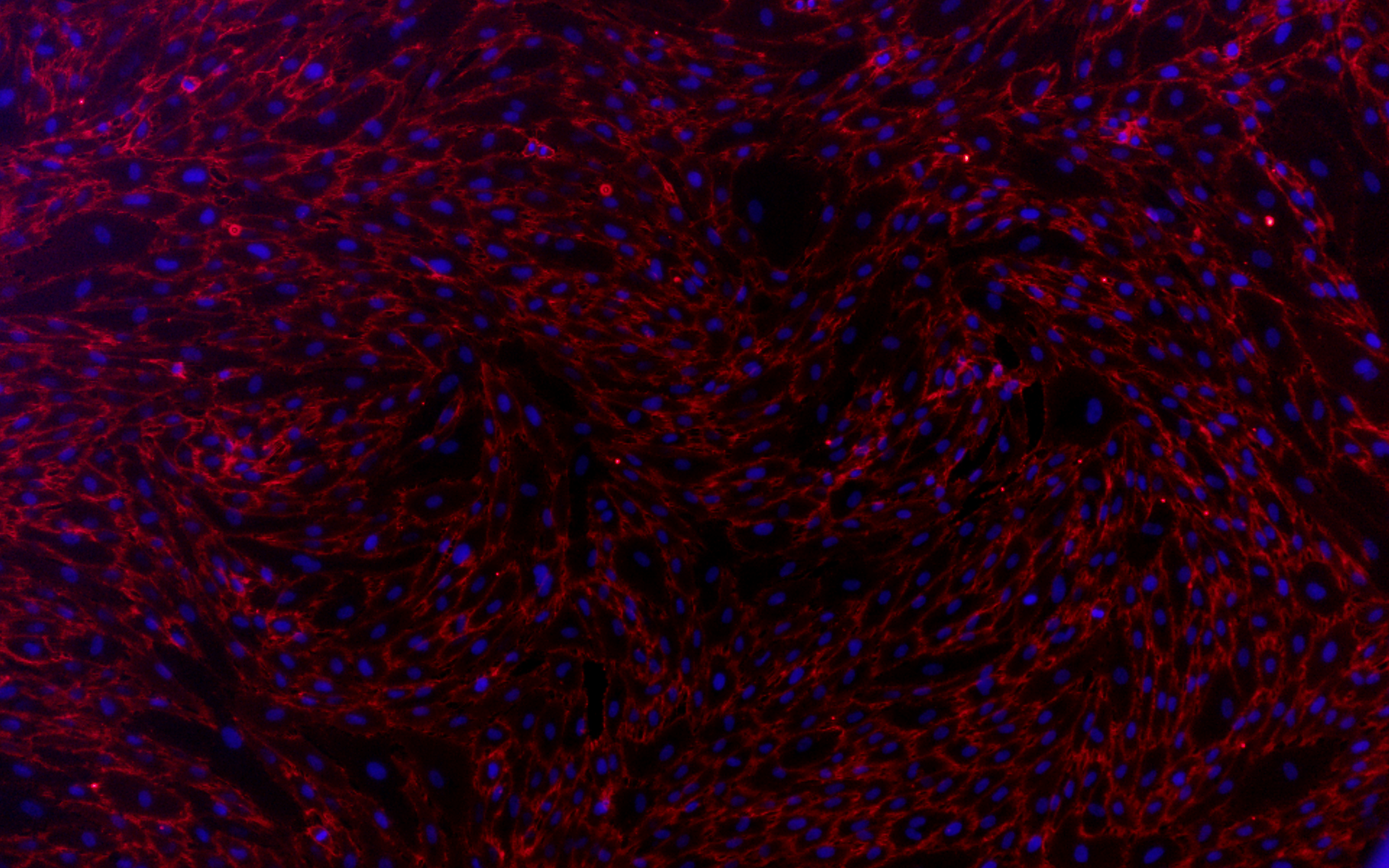

Supplement: S1 Dataset — (ZIP) [file pone.0261498.s001.zip › Fig 1/Fig 1 C3.tif]

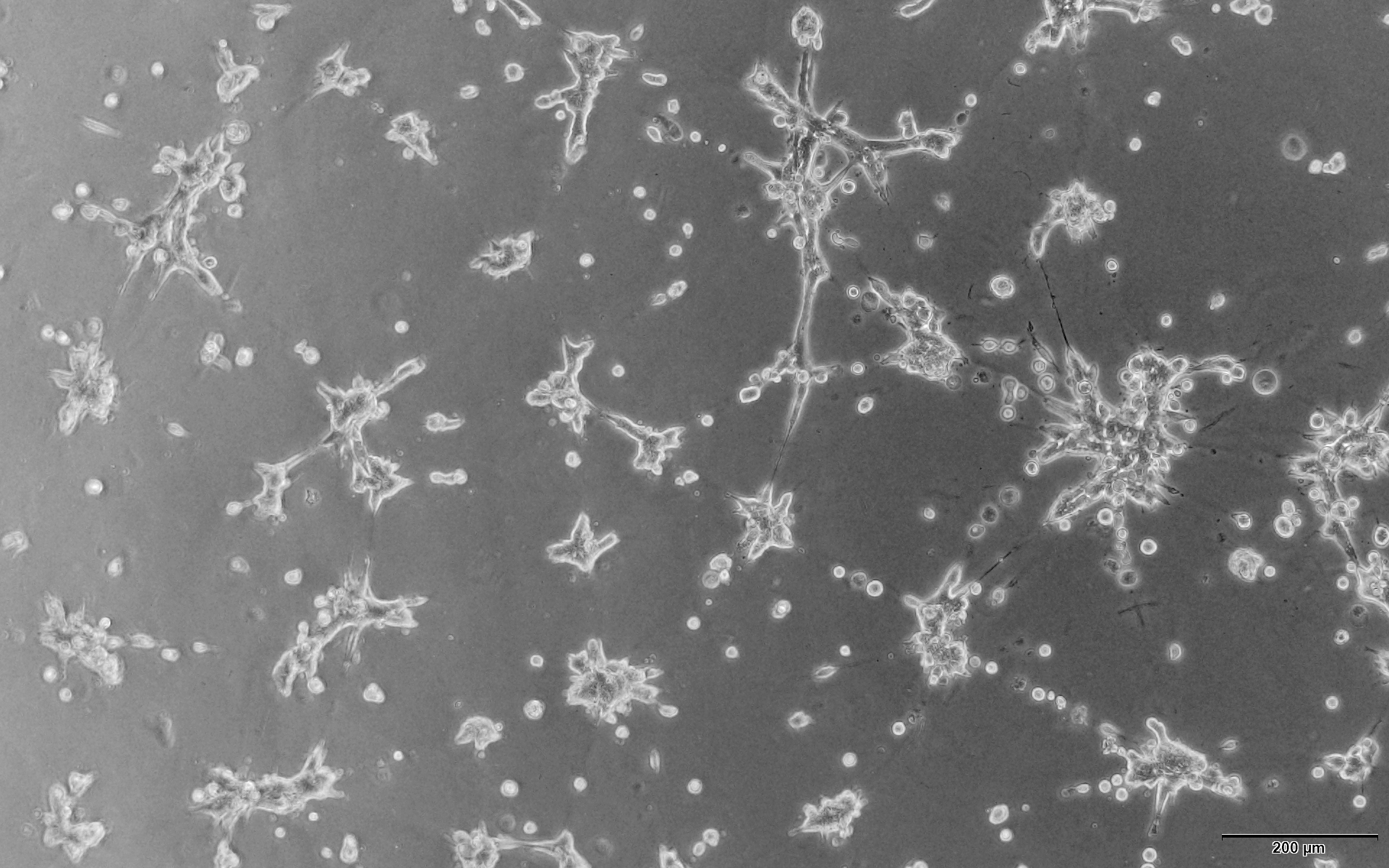

Supplement: S1 Dataset — (ZIP) [file pone.0261498.s001.zip › Fig 1/Fig 1 D1.tif]

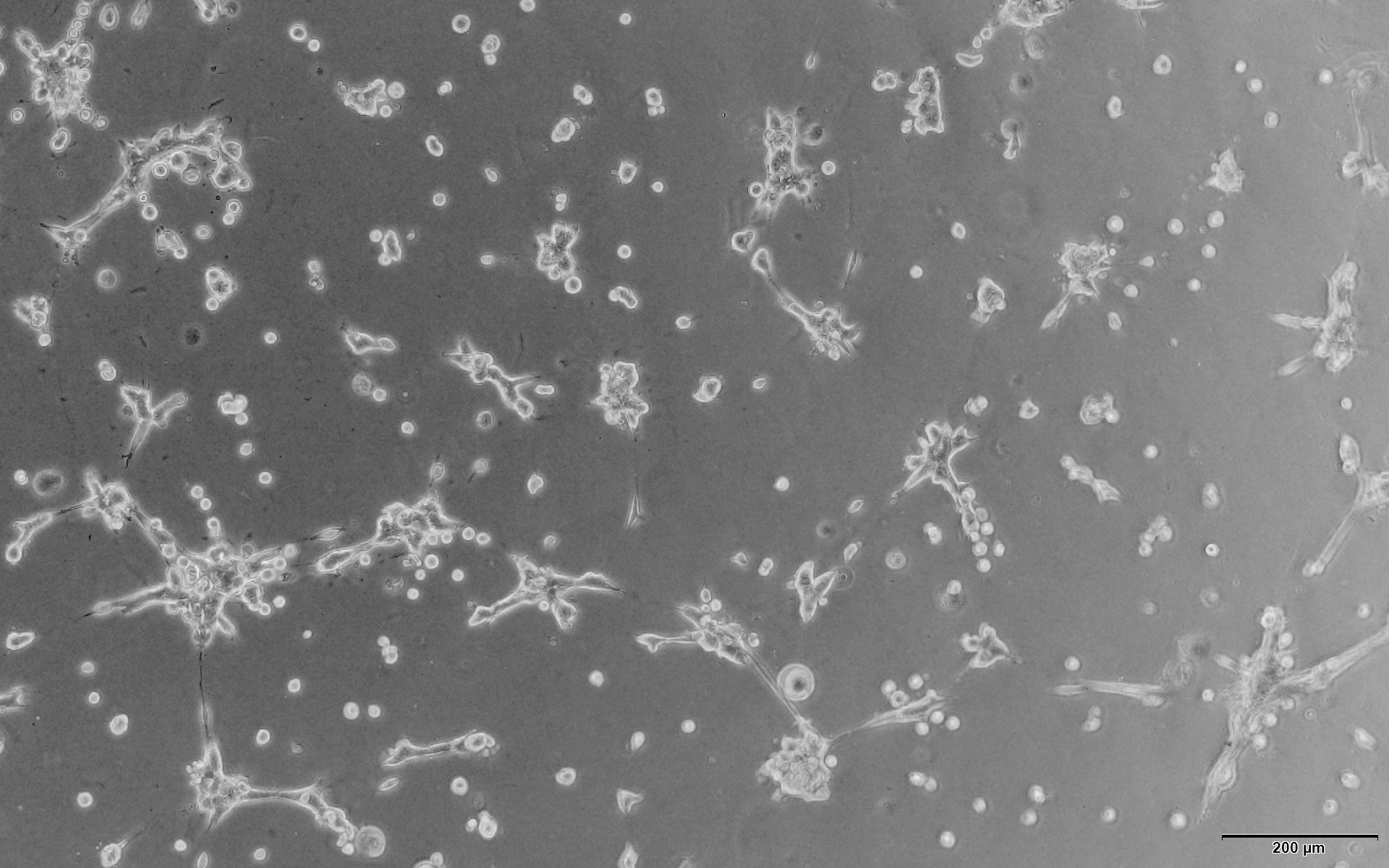

Supplement: S1 Dataset — (ZIP) [file pone.0261498.s001.zip › Fig 1/Fig 1 D2.tif]

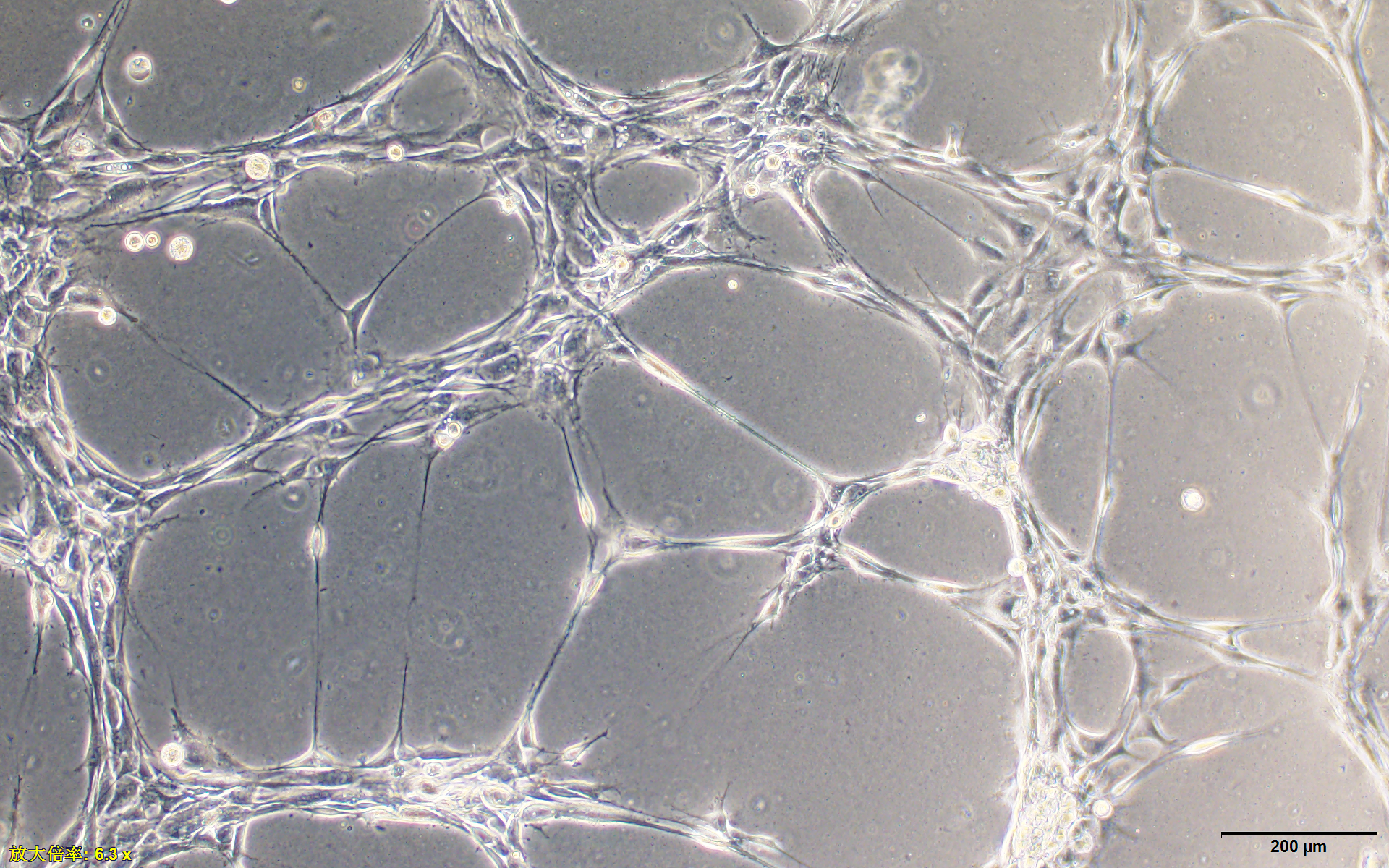

Supplement: S1 Dataset — (ZIP) [file pone.0261498.s001.zip › Fig 1/Fig 1 D3.tif]

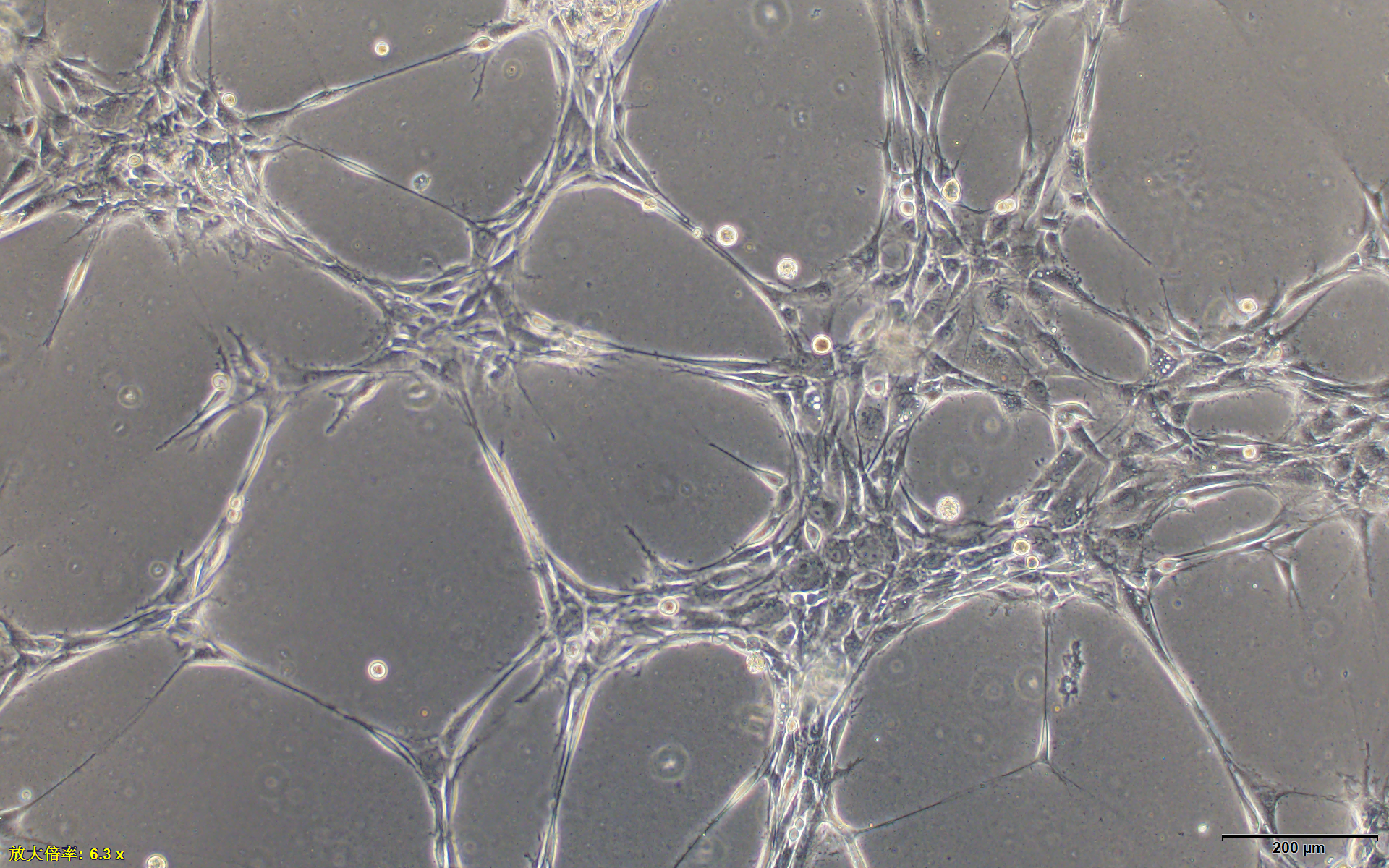

Supplement: S1 Dataset — (ZIP) [file pone.0261498.s001.zip › Fig 1/Fig 1 D4.tif]

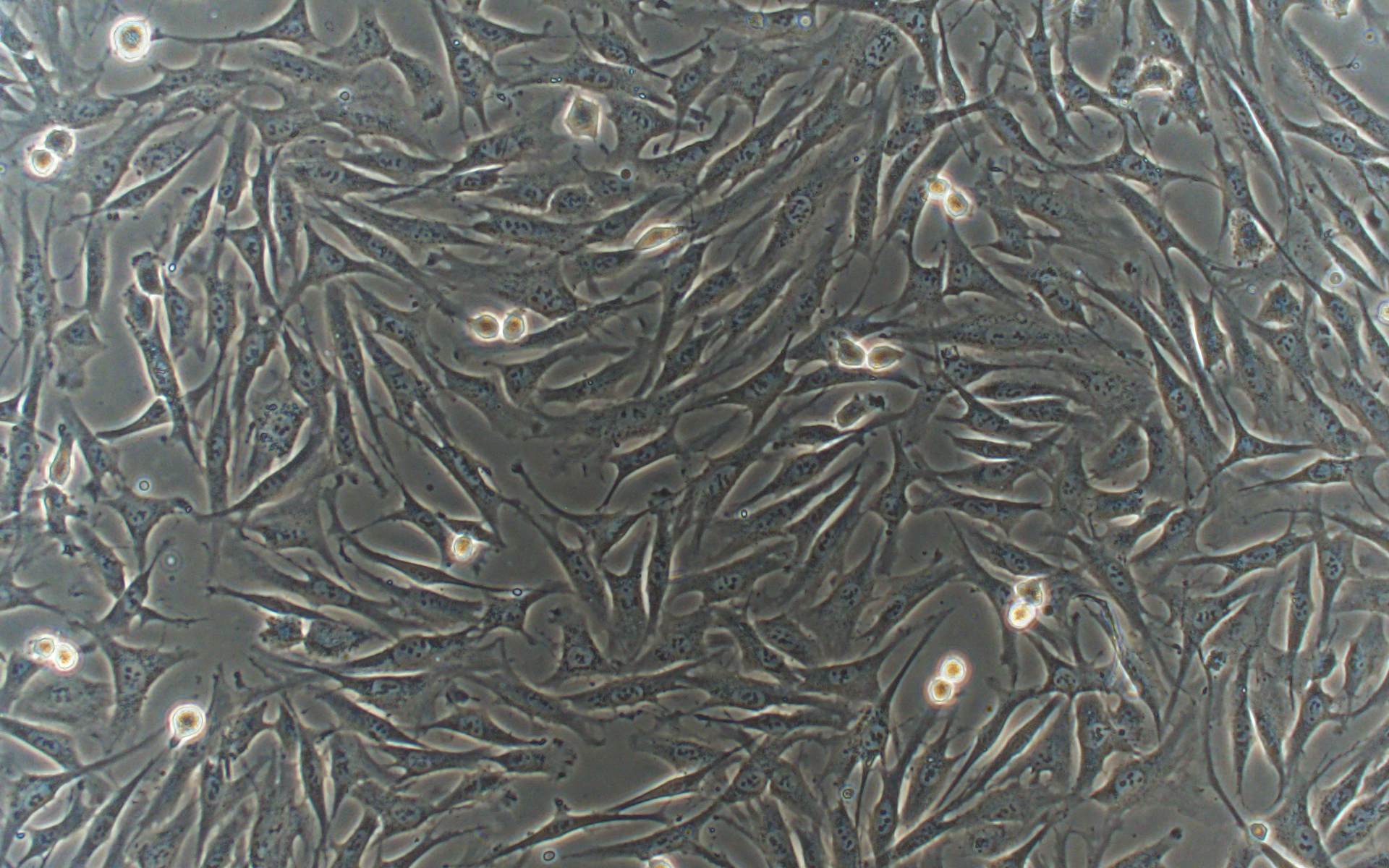

Supplement: S1 Dataset — (ZIP) [file pone.0261498.s001.zip › Fig 1/Fig 1 E1.tif]

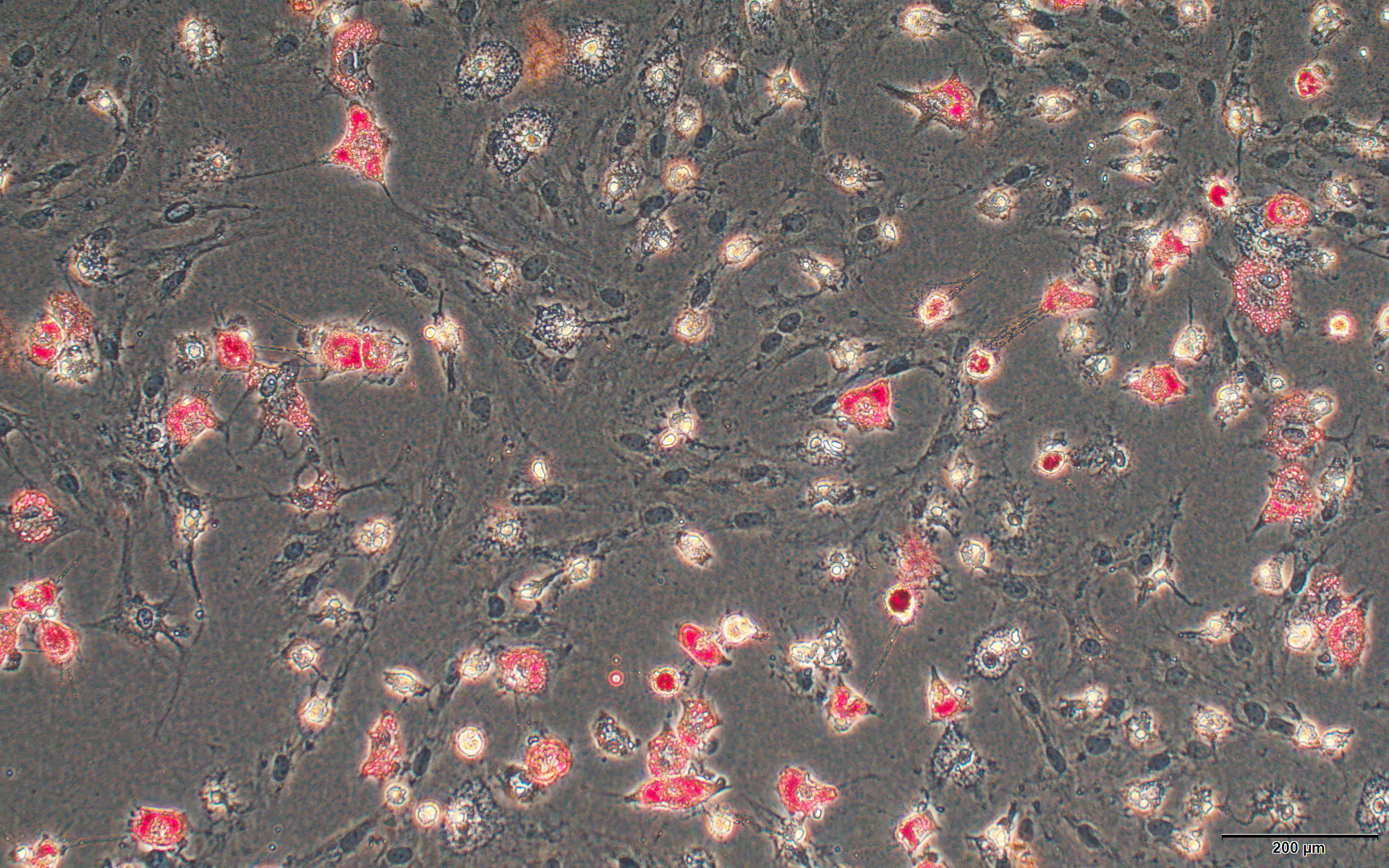

Supplement: S1 Dataset — (ZIP) [file pone.0261498.s001.zip › Fig 1/Fig 1 E2.tif]

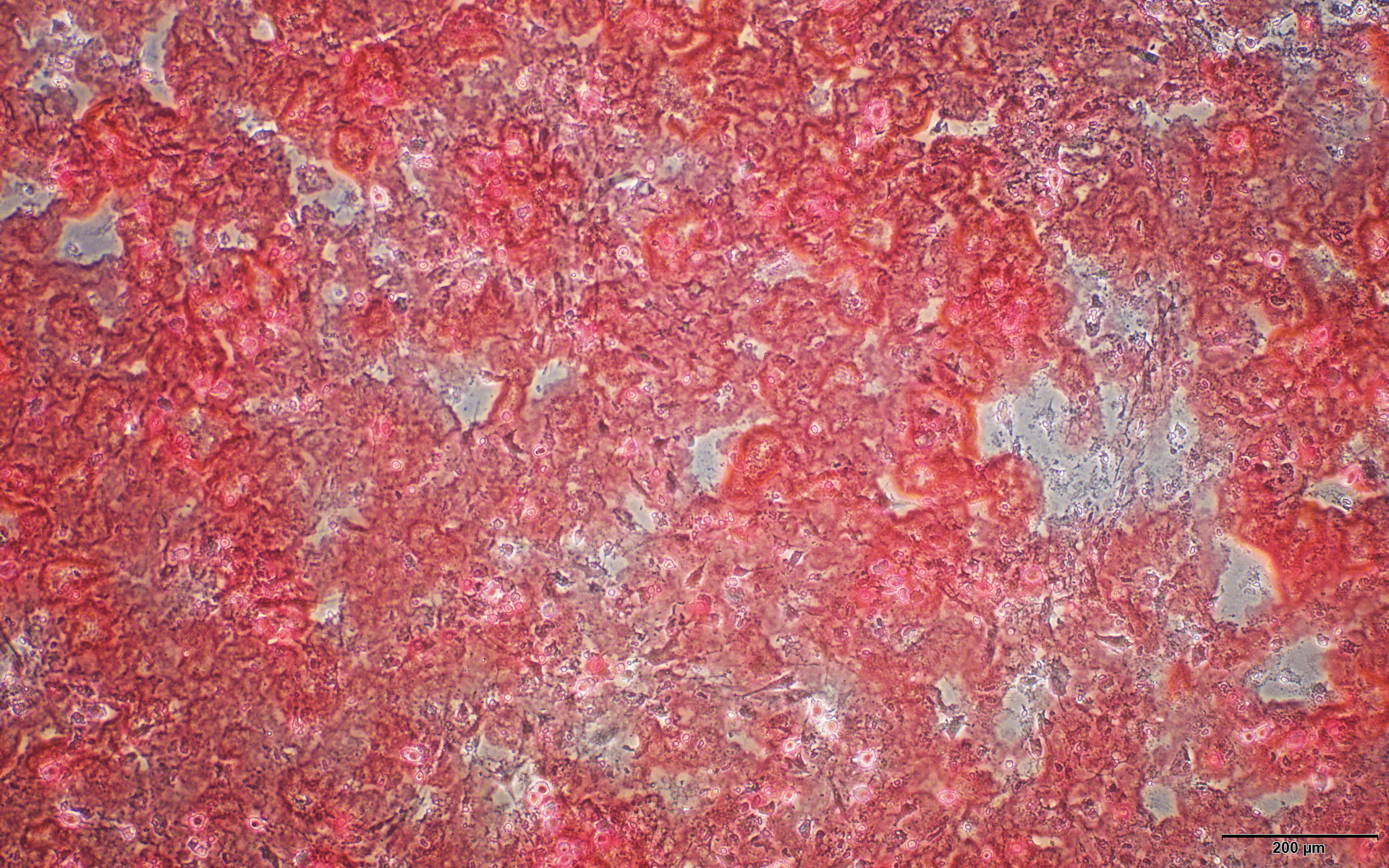

Supplement: S1 Dataset — (ZIP) [file pone.0261498.s001.zip › Fig 1/Fig 1 E3.tif]

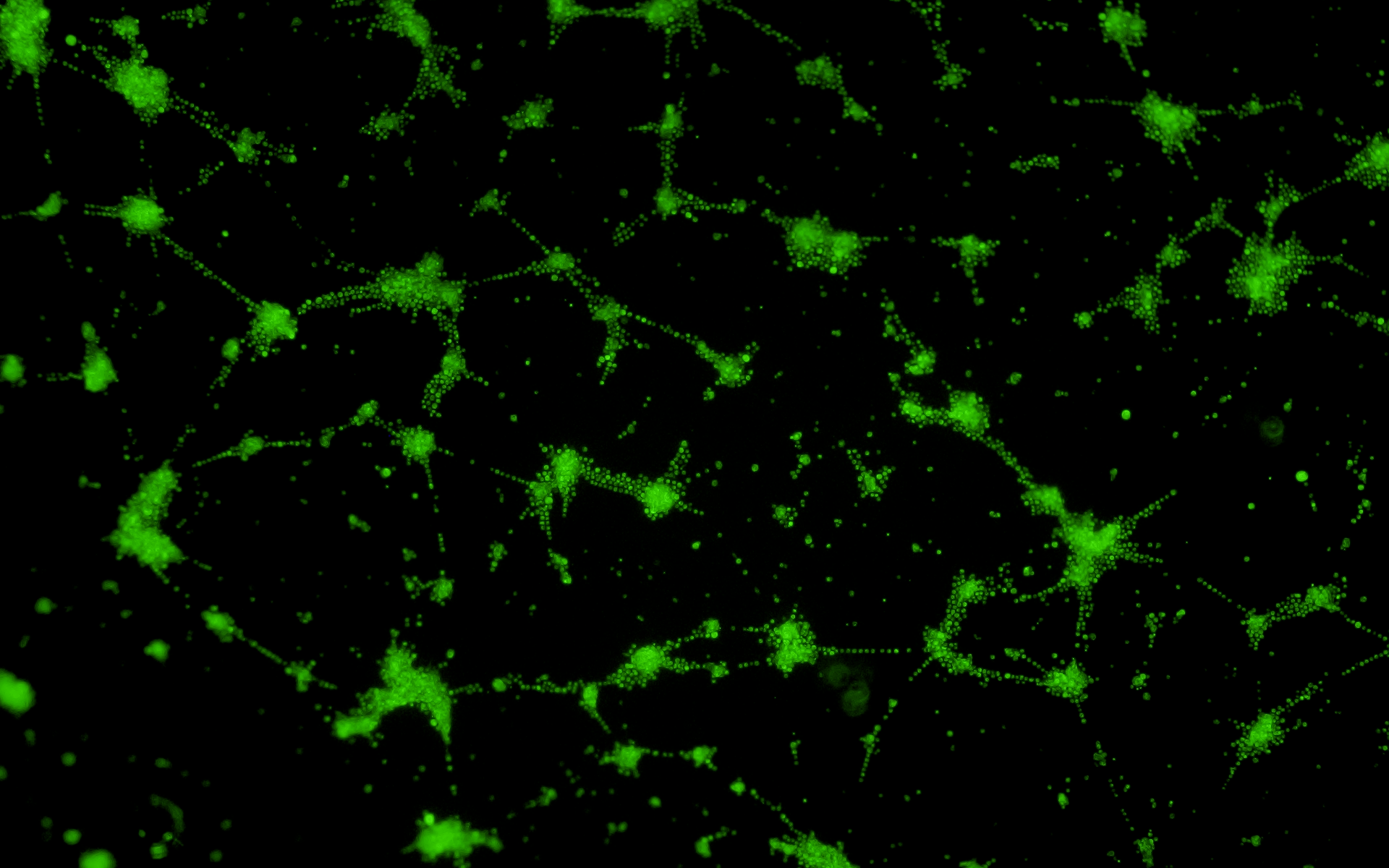

Supplement: S3 Dataset — (ZIP) [file pone.0261498.s003.zip › Fig 3/Fig 3 A.tif]

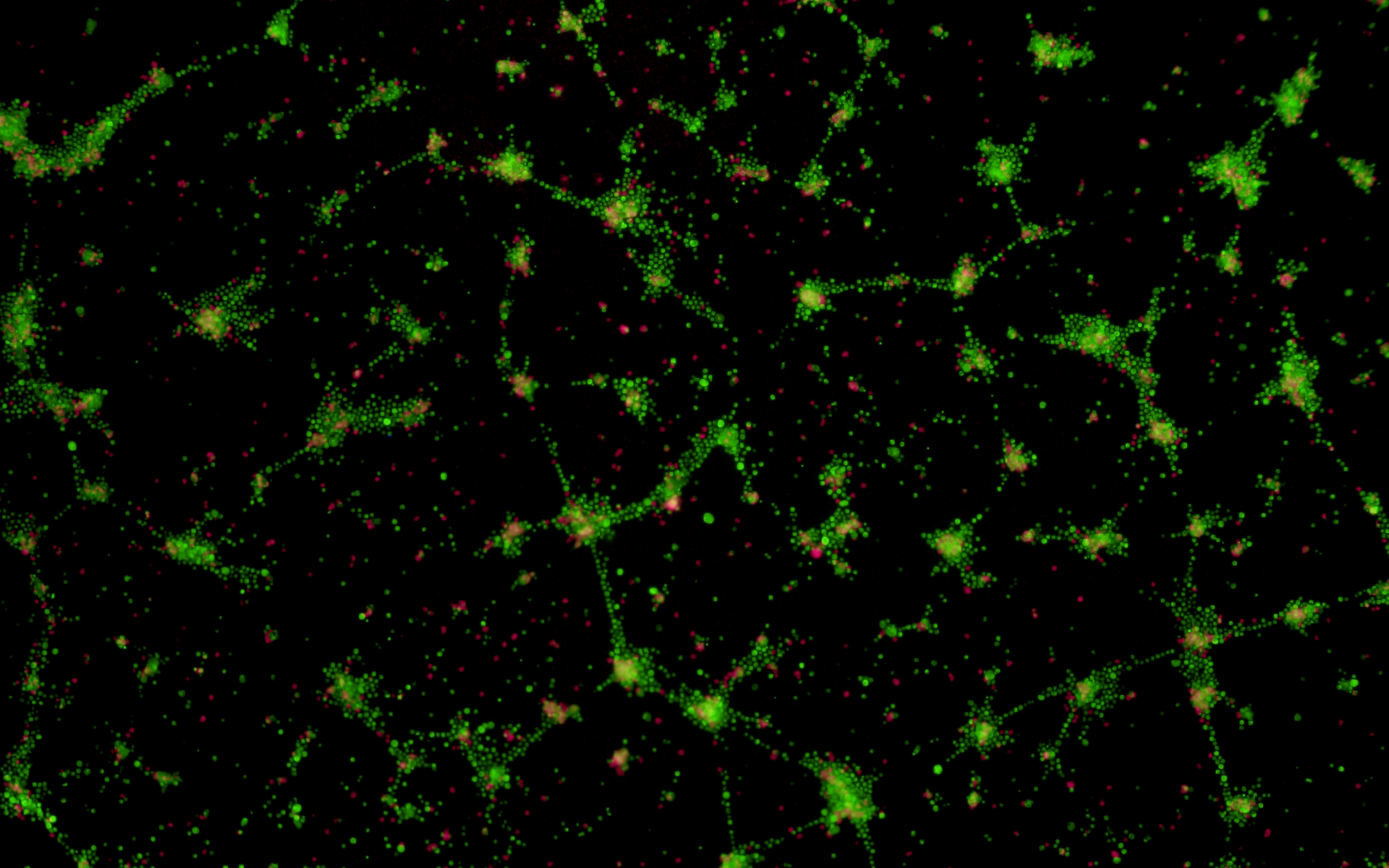

Supplement: S3 Dataset — (ZIP) [file pone.0261498.s003.zip › Fig 3/Fig 3 B.tif]

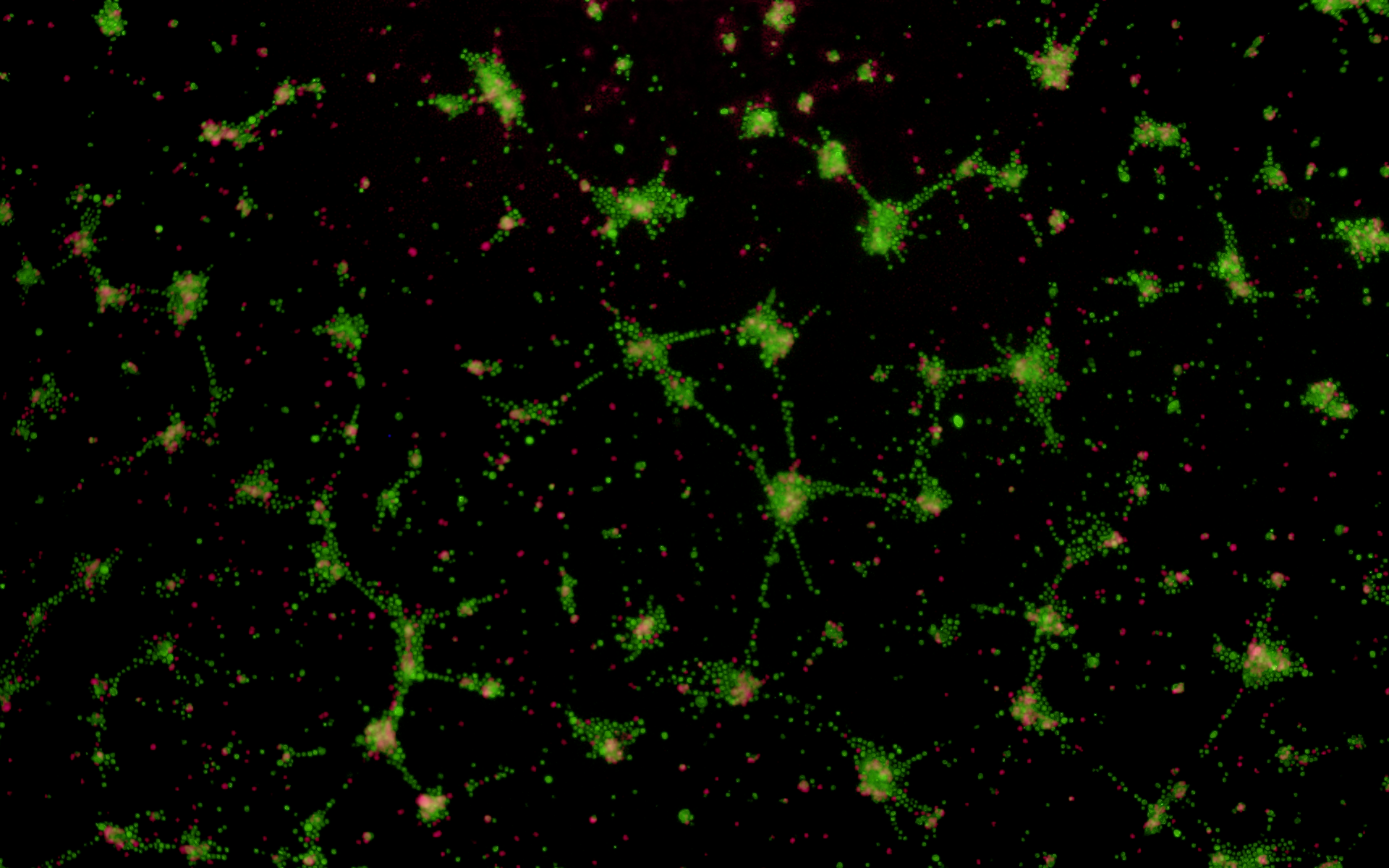

Supplement: S3 Dataset — (ZIP) [file pone.0261498.s003.zip › Fig 3/Fig 3 C.tif]

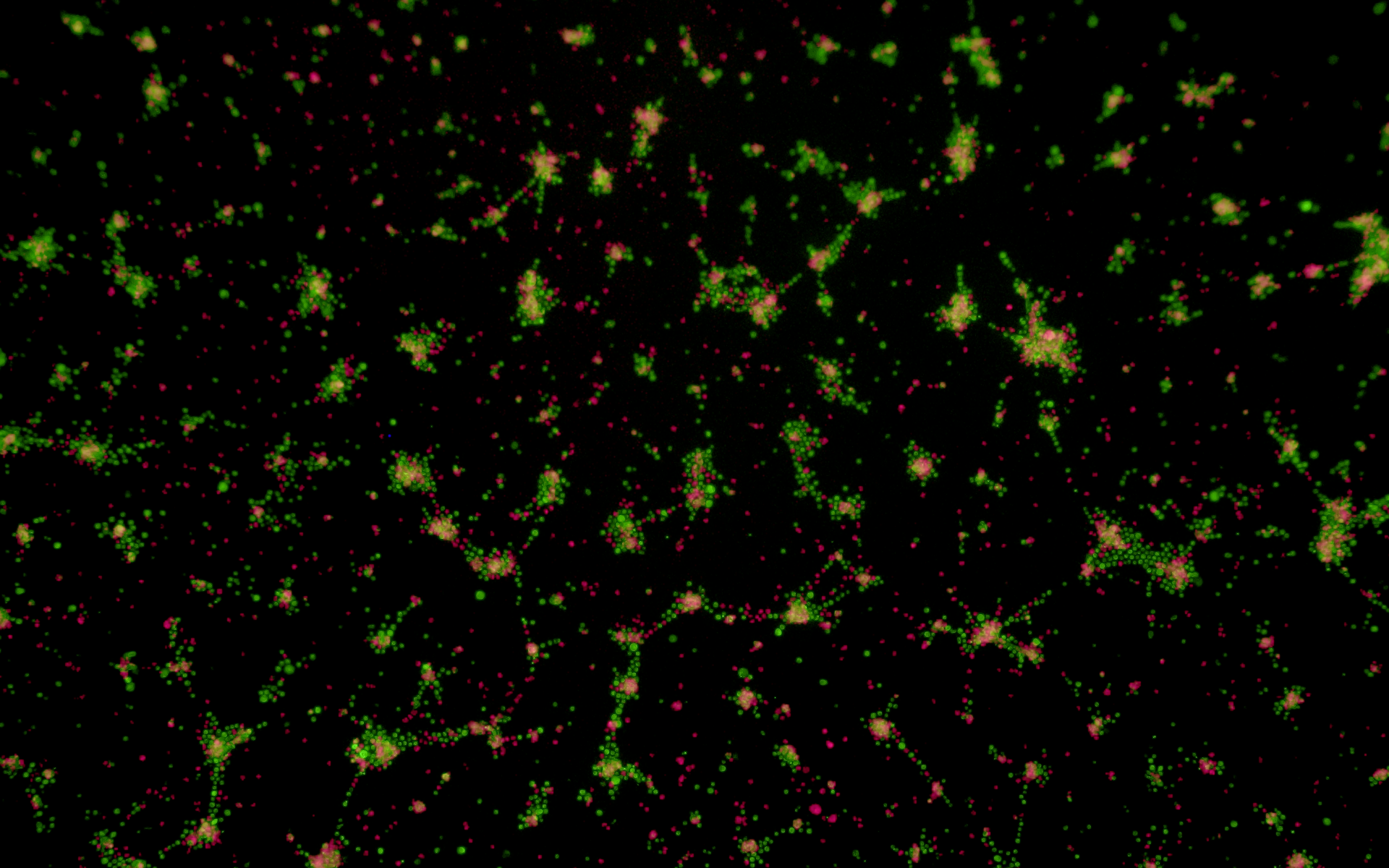

Supplement: S3 Dataset — (ZIP) [file pone.0261498.s003.zip › Fig 3/Fig 3 D.tif]

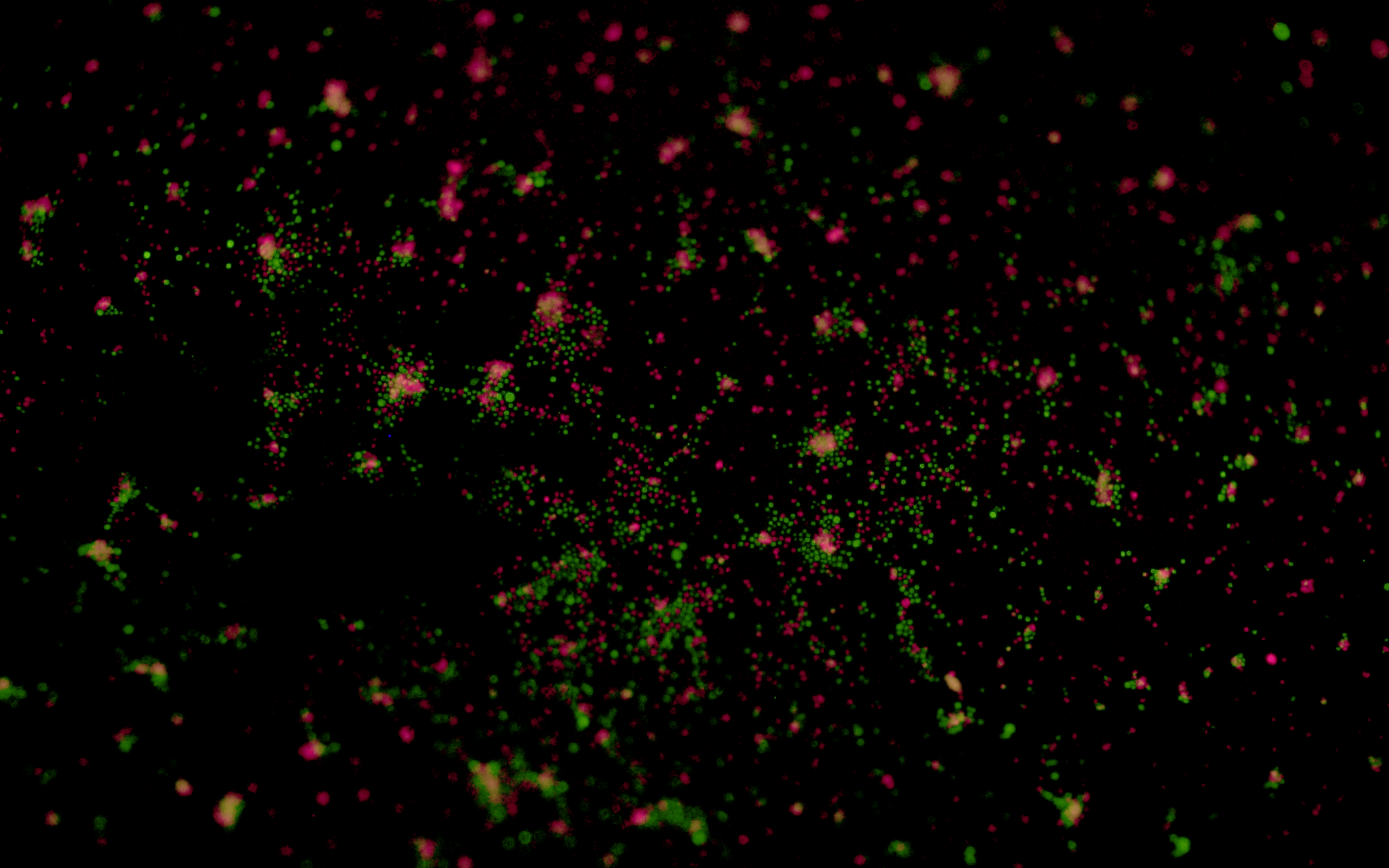

Supplement: S3 Dataset — (ZIP) [file pone.0261498.s003.zip › Fig 3/Fig 3 E.tif]

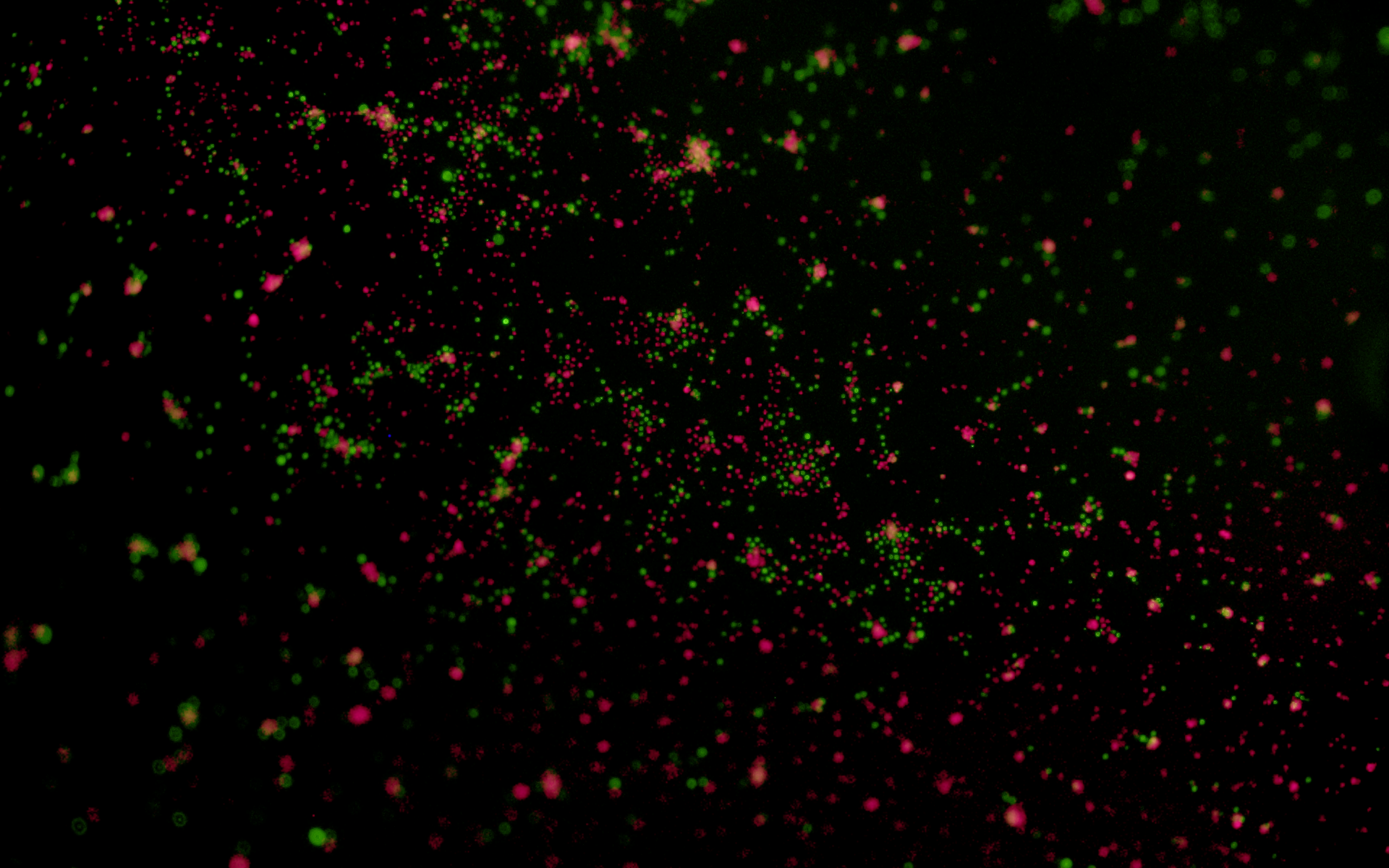

Supplement: S3 Dataset — (ZIP) [file pone.0261498.s003.zip › Fig 3/Fig 3 F.tif]

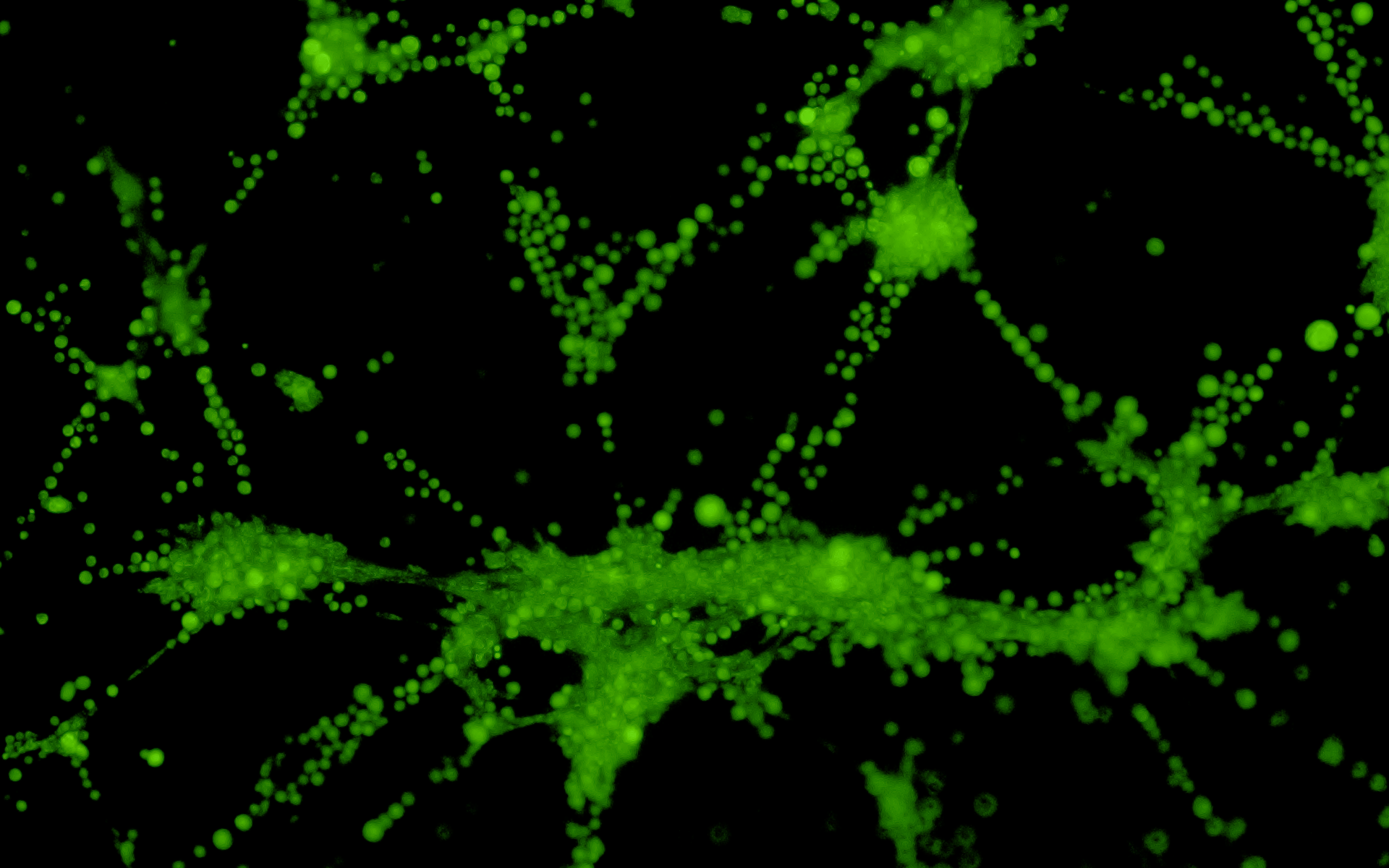

Supplement: S3 Dataset — (ZIP) [file pone.0261498.s003.zip › Fig 3/Fig 3 J1.tif]

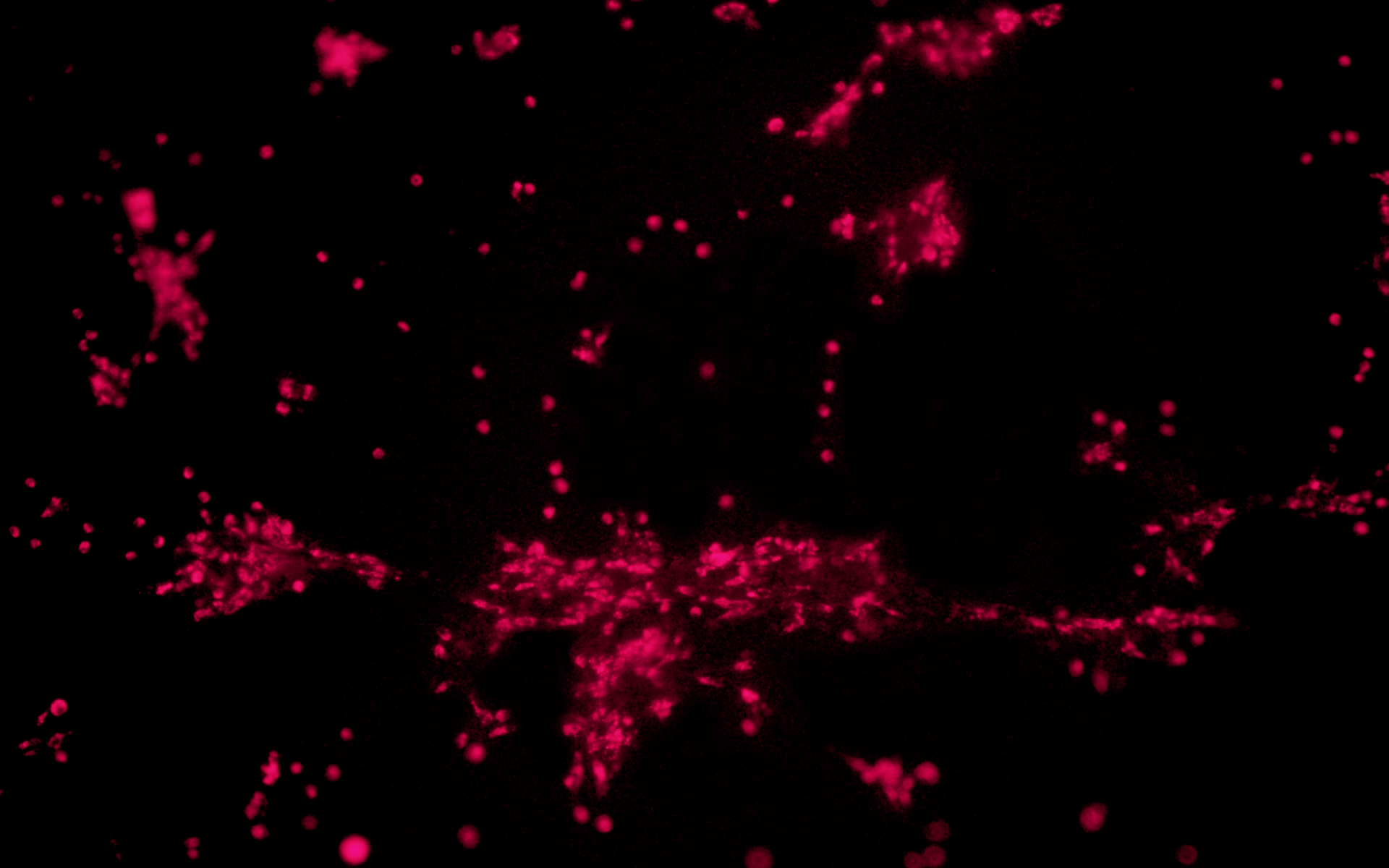

Supplement: S3 Dataset — (ZIP) [file pone.0261498.s003.zip › Fig 3/Fig 3 J2.tif]

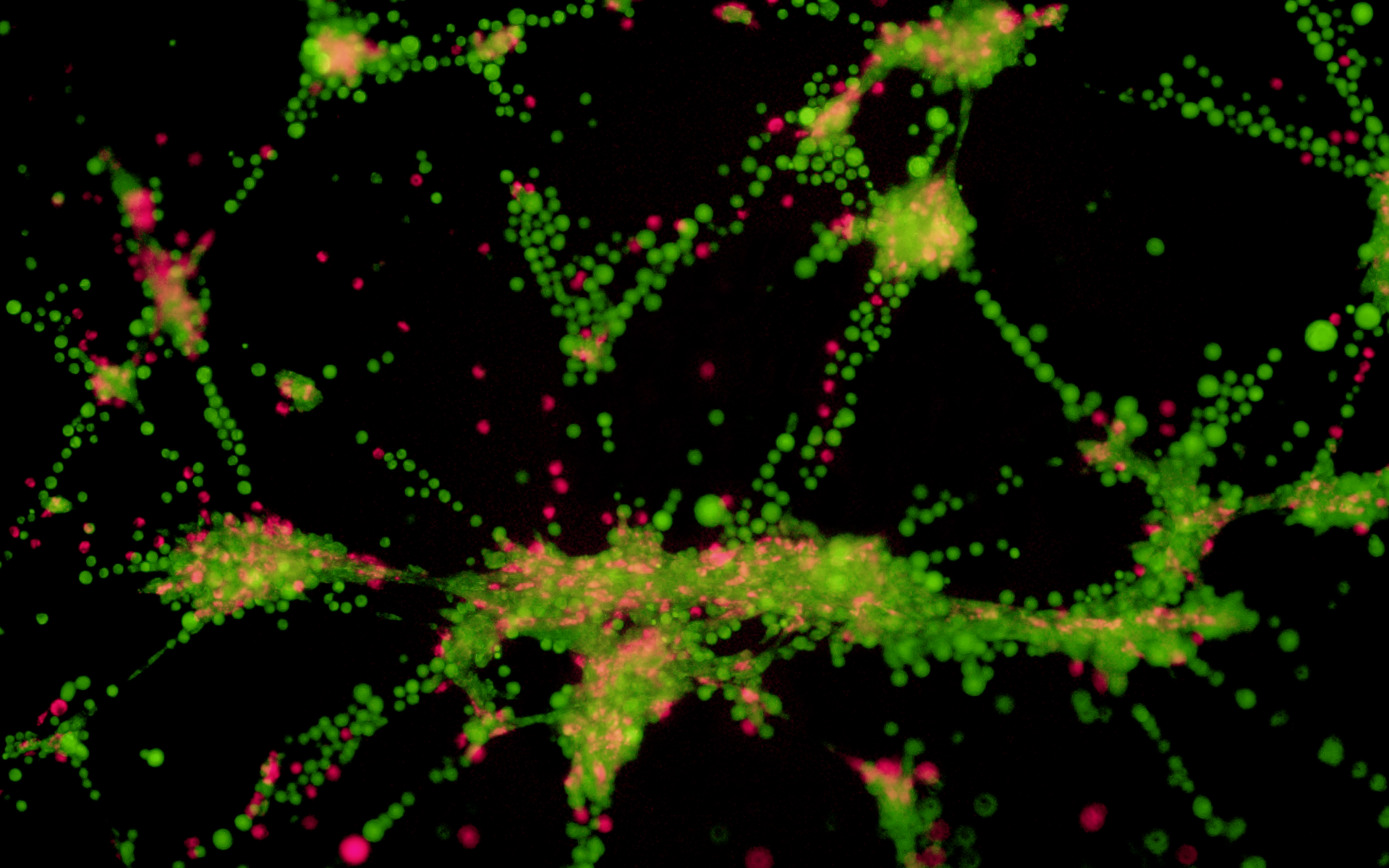

Supplement: S3 Dataset — (ZIP) [file pone.0261498.s003.zip › Fig 3/Fig 3 J3.tif]

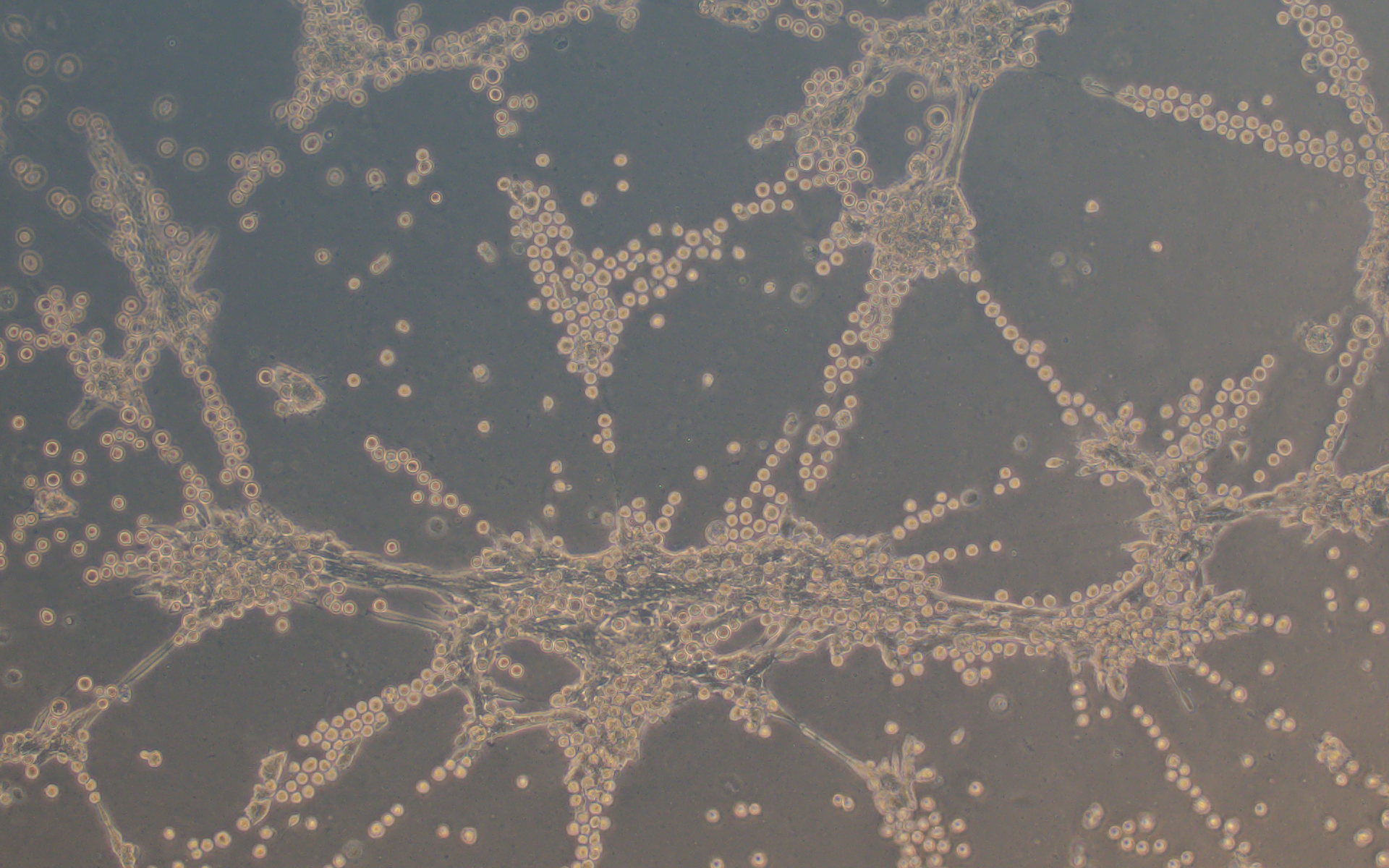

Supplement: S3 Dataset — (ZIP) [file pone.0261498.s003.zip › Fig 3/Fig 3 J4.tif]

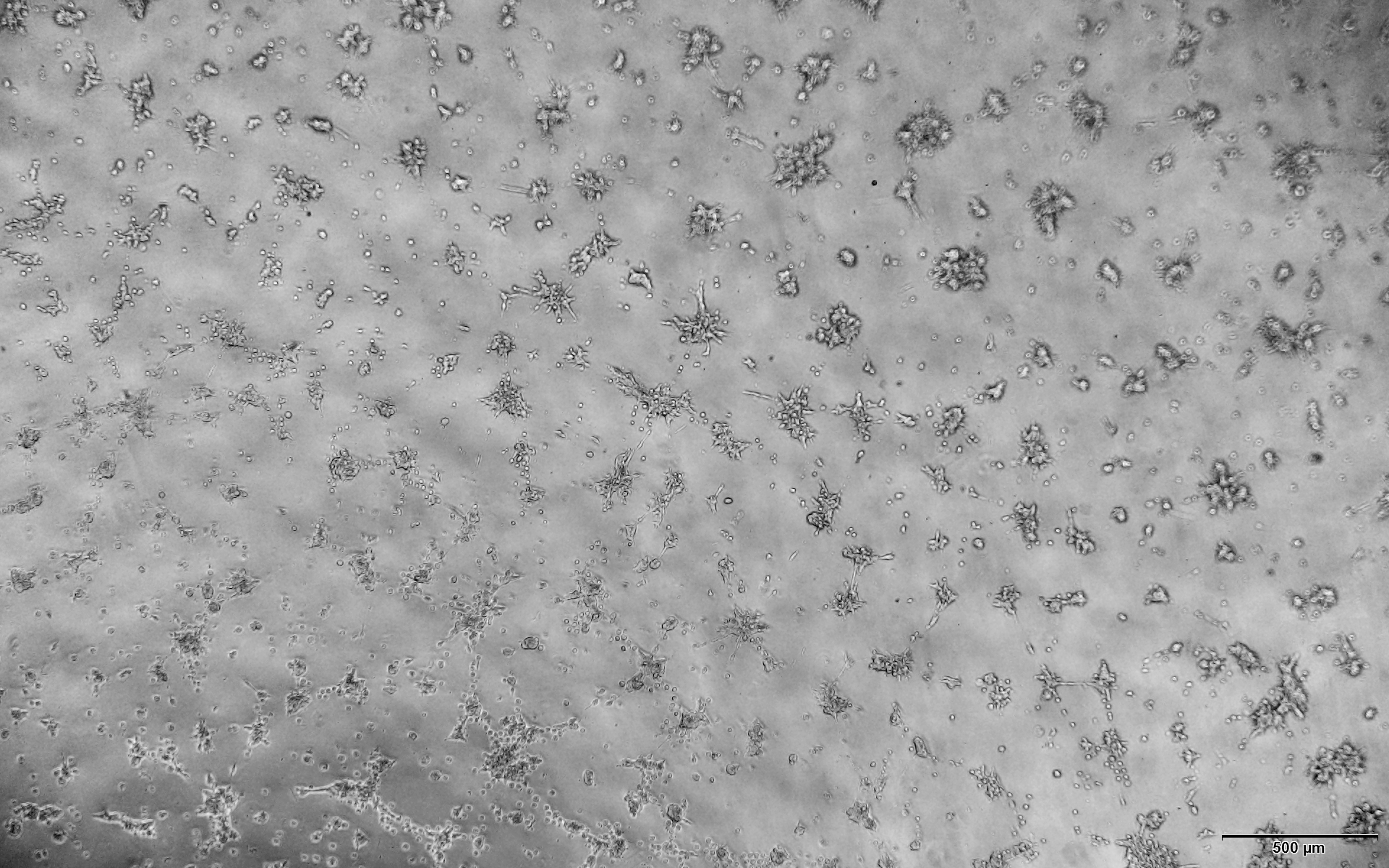

Supplement: S4 Dataset — (ZIP) [file pone.0261498.s004.zip › Fig 4/Fig 4 A1 (EGM2 assay medium 4X).tif]

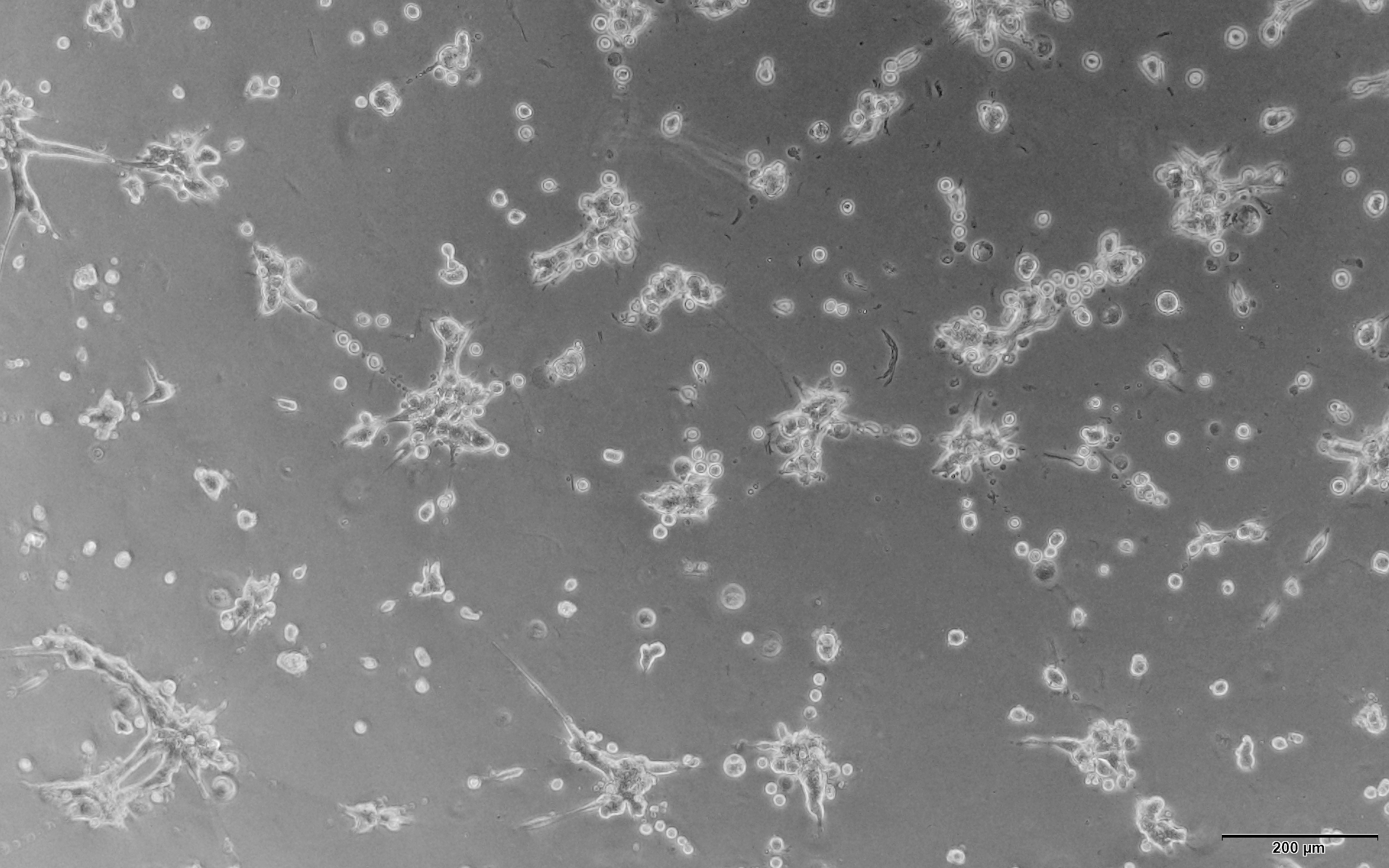

Supplement: S4 Dataset — (ZIP) [file pone.0261498.s004.zip › Fig 4/Fig 4 A2 (EGM2 assay medium 10X).tif]

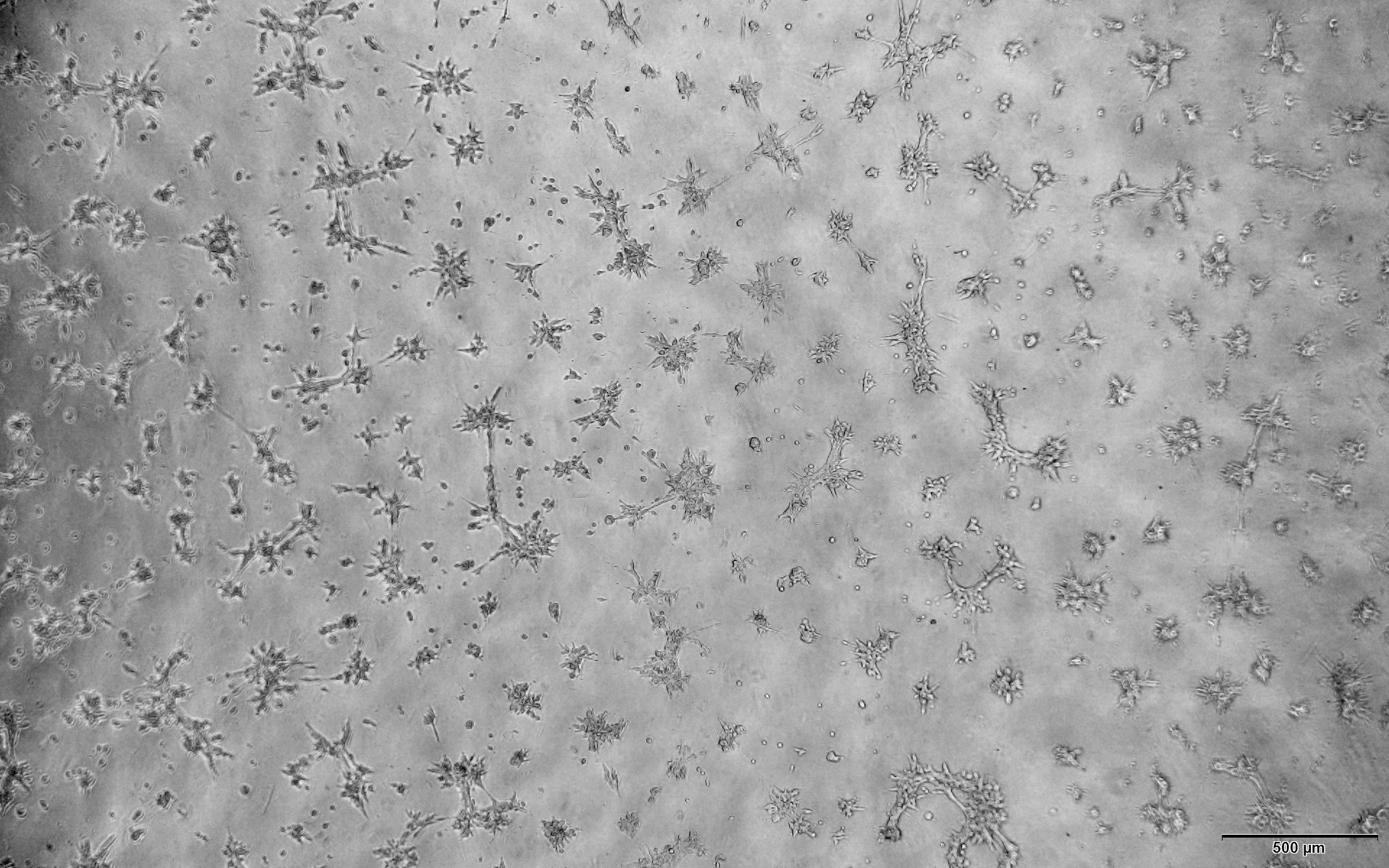

Supplement: S4 Dataset — (ZIP) [file pone.0261498.s004.zip › Fig 4/Fig 4 B1 (EGM2 complete medium 4X).tif]

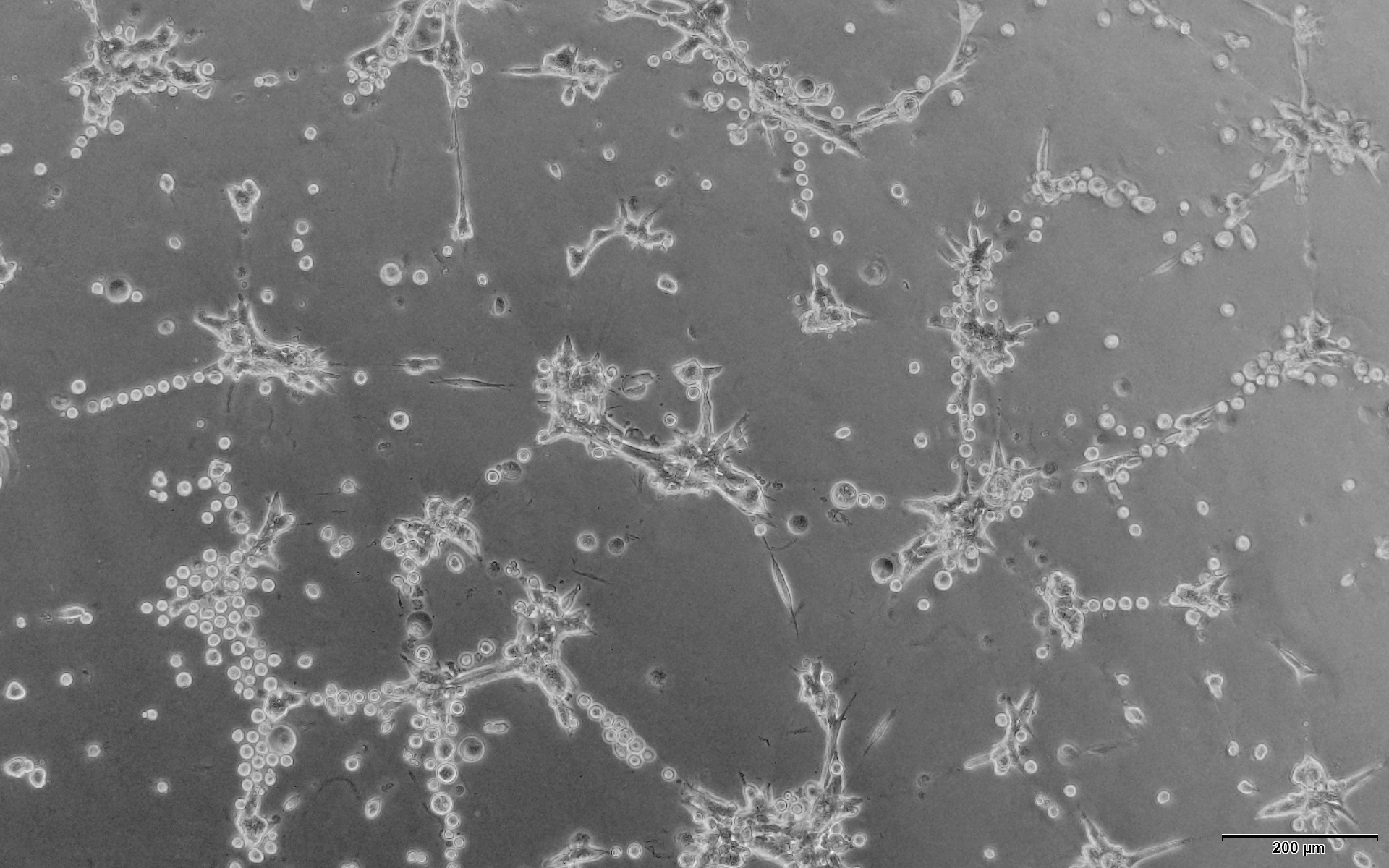

Supplement: S4 Dataset — (ZIP) [file pone.0261498.s004.zip › Fig 4/Fig 4 B2 (EGM2 complete medium 10X).tif]

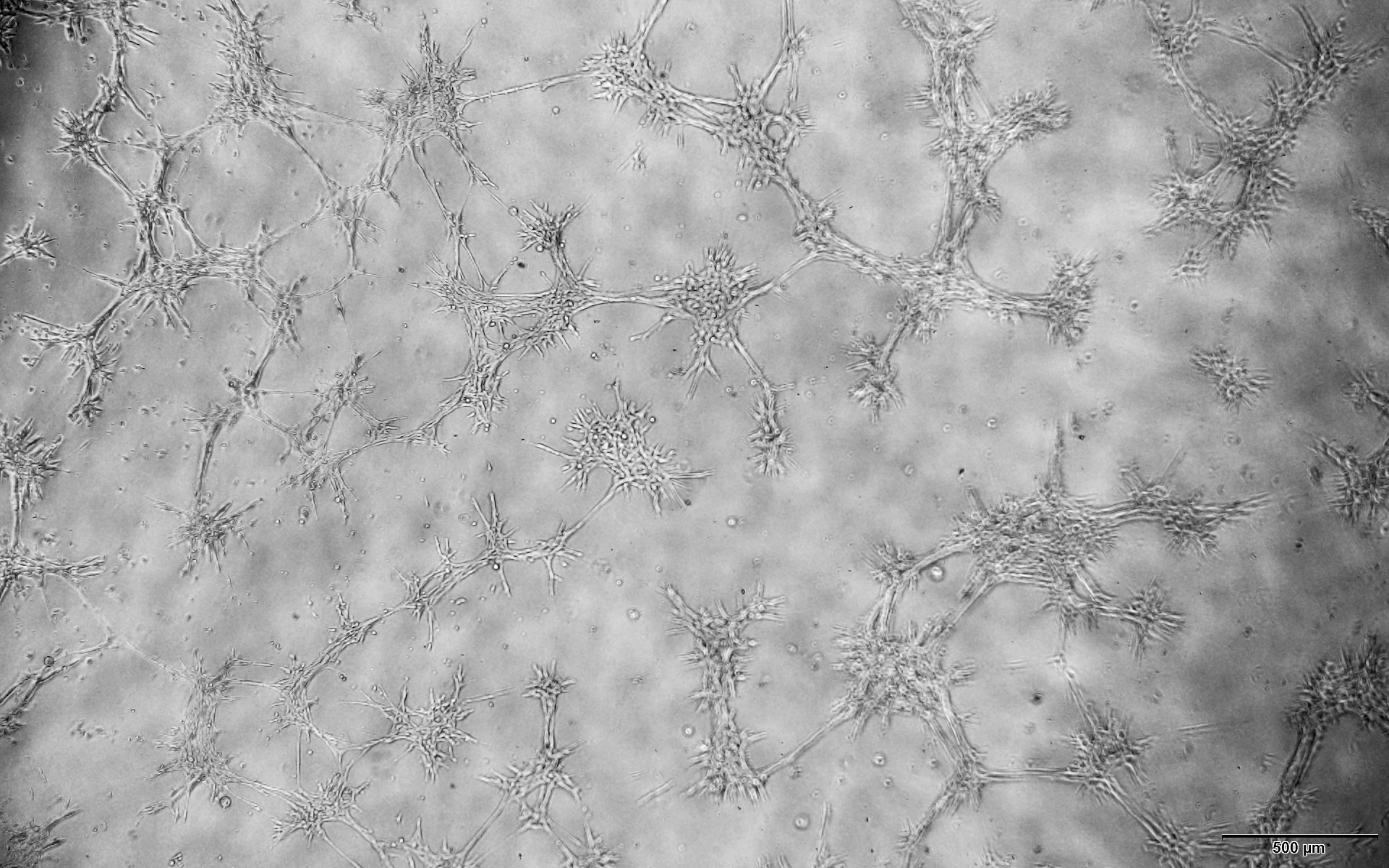

Supplement: S4 Dataset — (ZIP) [file pone.0261498.s004.zip › Fig 4/Fig 4 C1 (ASC supernatant 4X).tif]

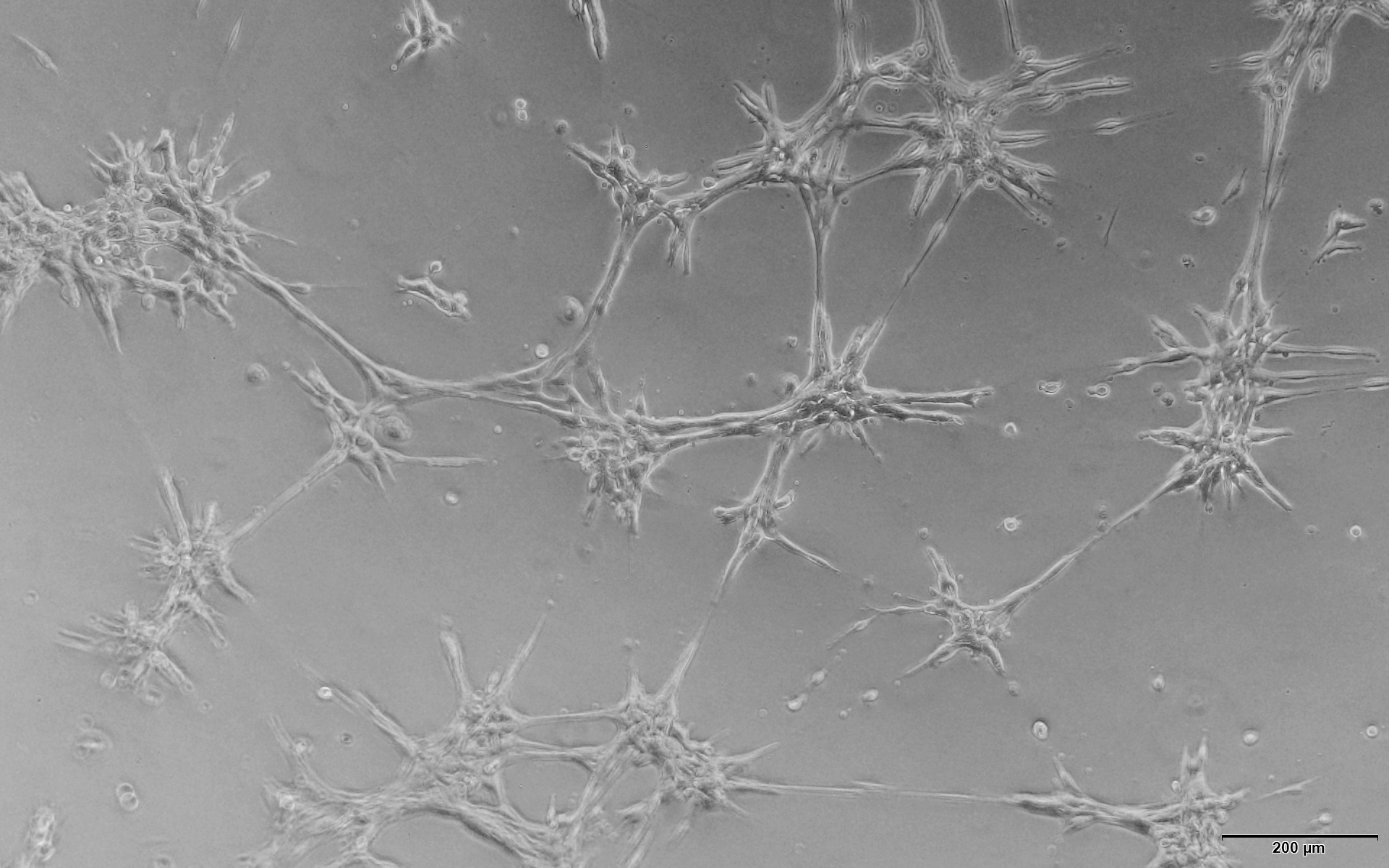

Supplement: S4 Dataset — (ZIP) [file pone.0261498.s004.zip › Fig 4/Fig 4 C2 (ASC supernatant 10X).tif]

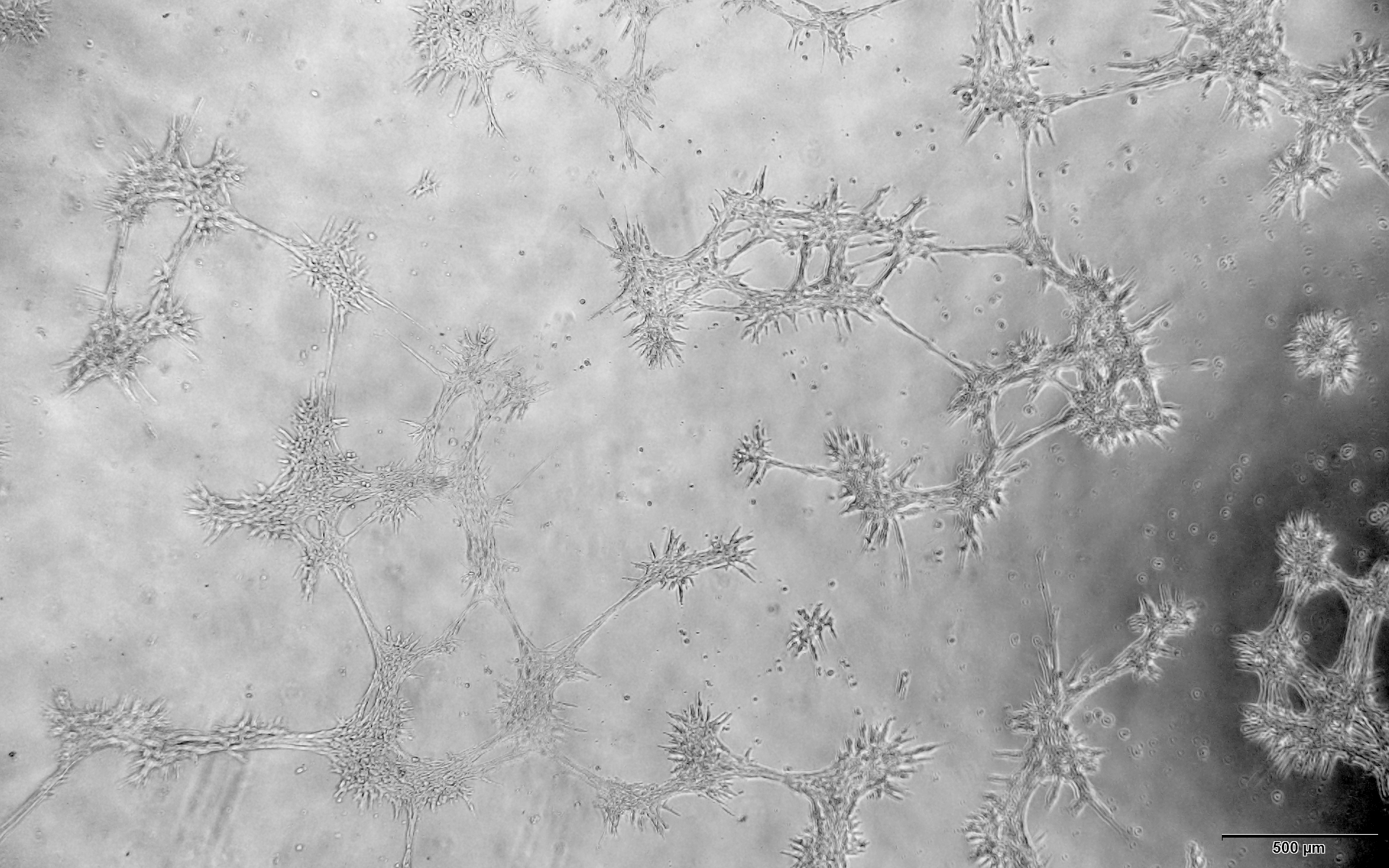

Supplement: S4 Dataset — (ZIP) [file pone.0261498.s004.zip › Fig 4/Fig 4 D1 (EPC supernatant 4X).tif]

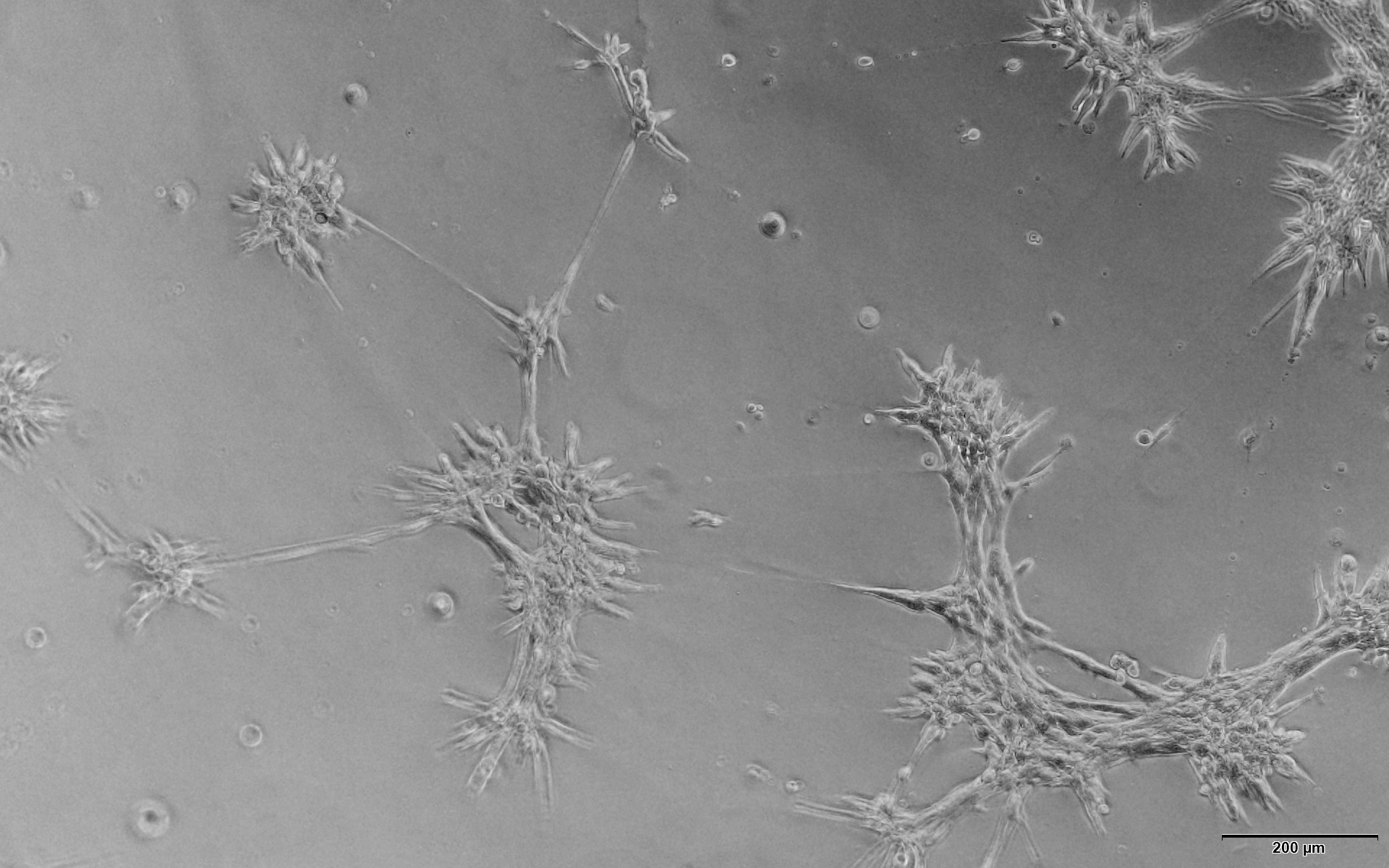

Supplement: S4 Dataset — (ZIP) [file pone.0261498.s004.zip › Fig 4/Fig 4 D2 (EPC supernatant 10X).tif]

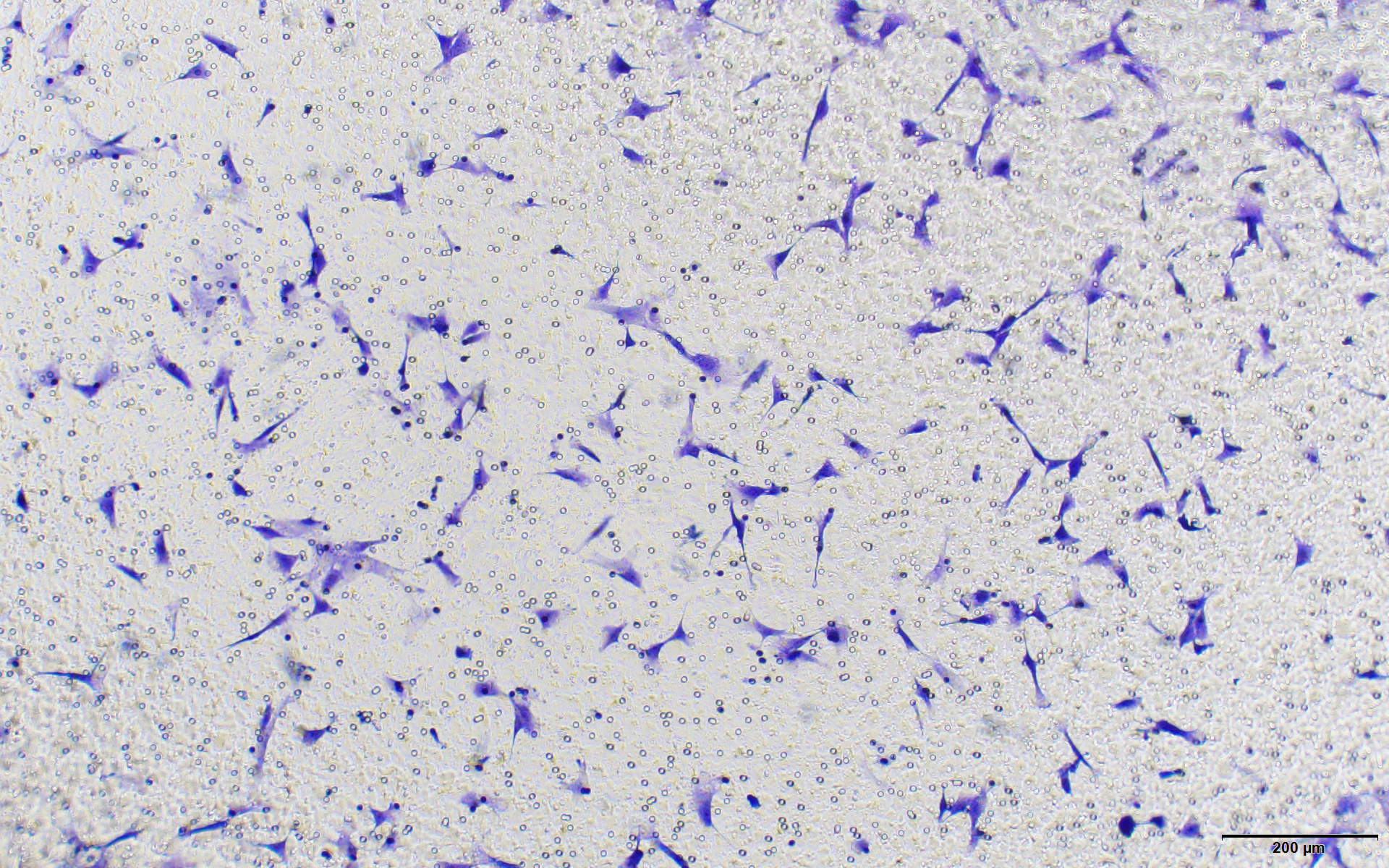

Supplement: S5 Dataset — (ZIP) [file pone.0261498.s005.zip › Fig 5/Fig 5 A1ú¿Transmigration-EGM2 assay medium).tif]

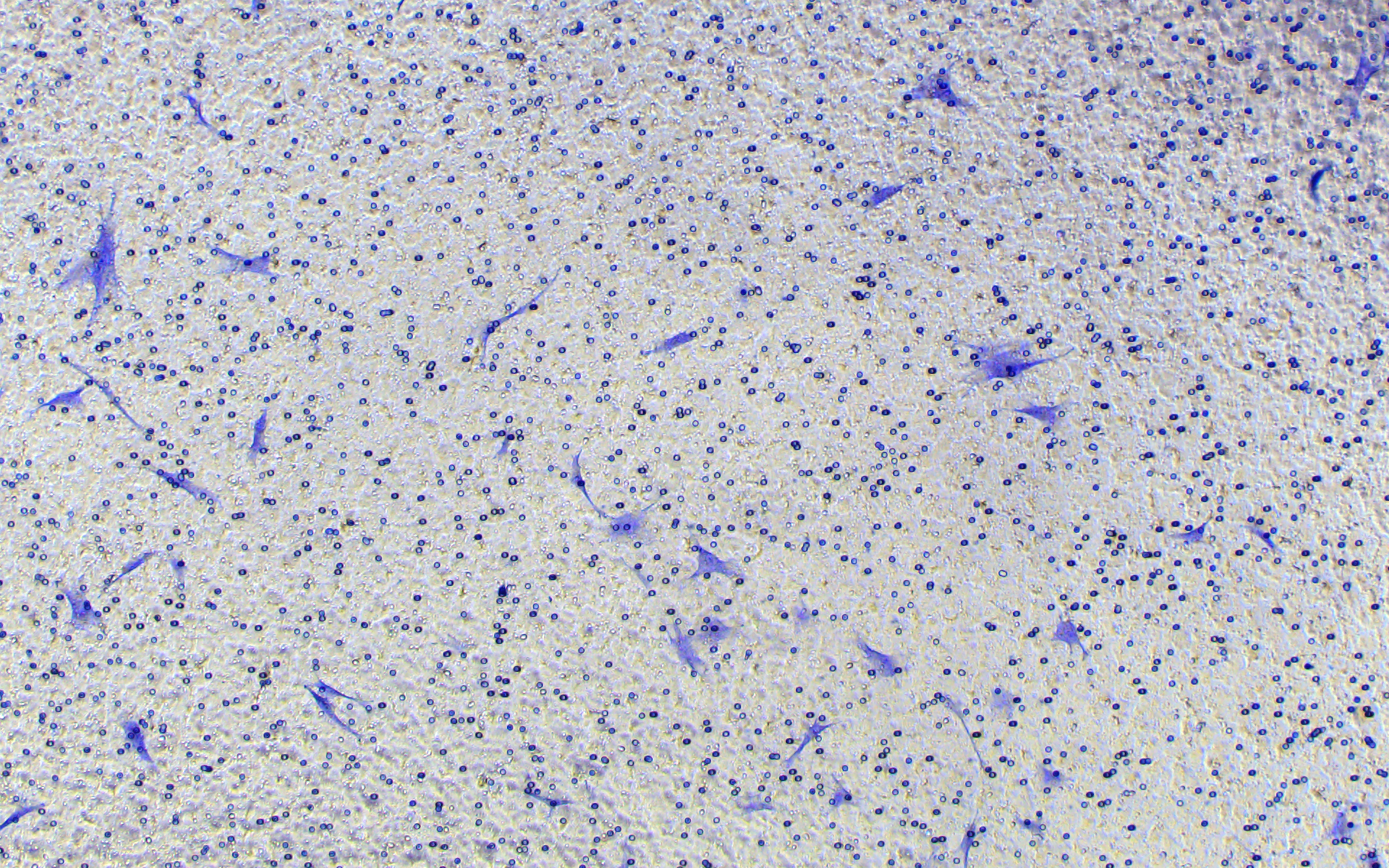

Supplement: S5 Dataset — (ZIP) [file pone.0261498.s005.zip › Fig 5/Fig 5 A1ú¿invasion-EGM2 assay medium).tif]

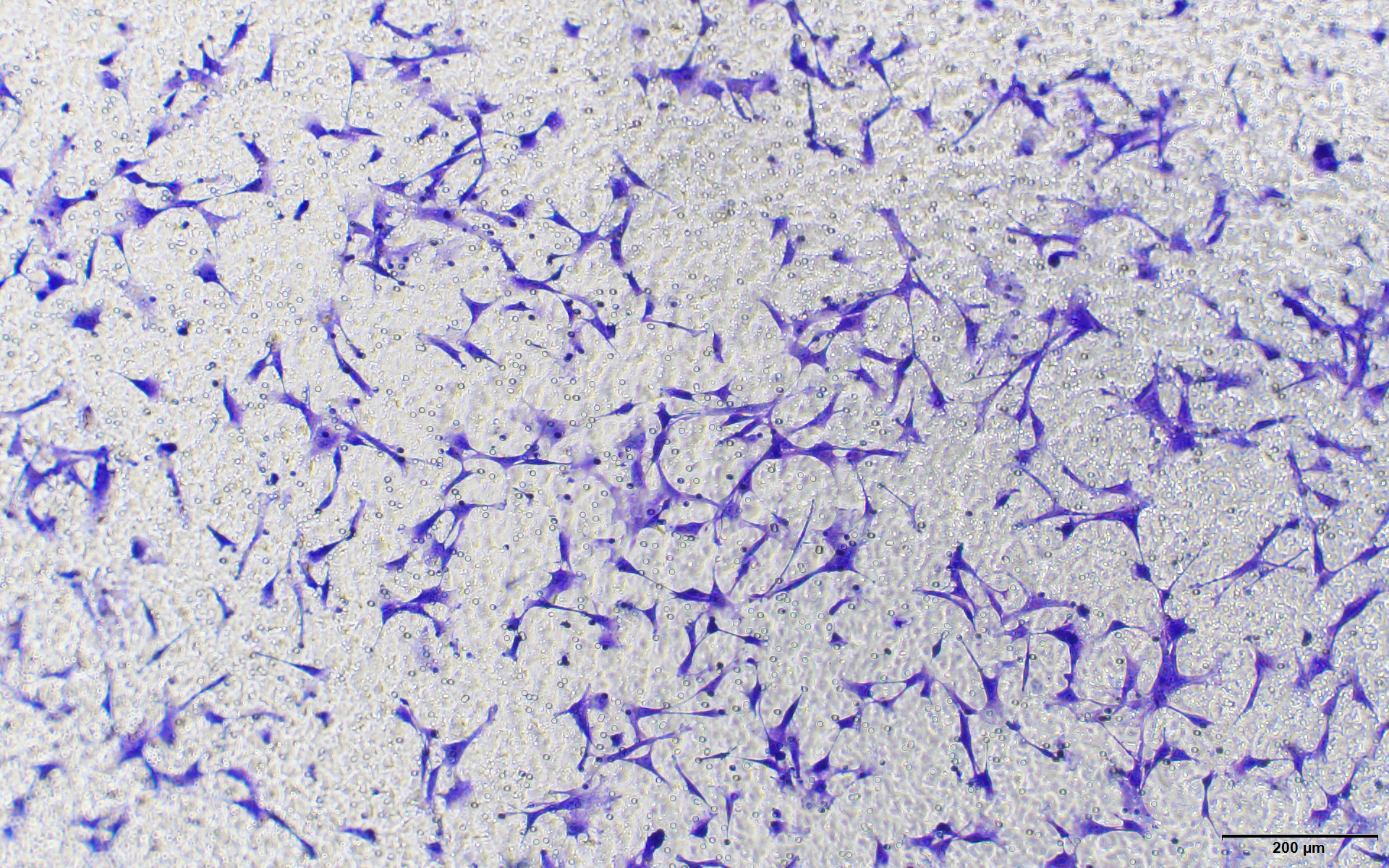

Supplement: S5 Dataset — (ZIP) [file pone.0261498.s005.zip › Fig 5/Fig 5 A2ú¿Transmigration-EPC supernatant).tif]

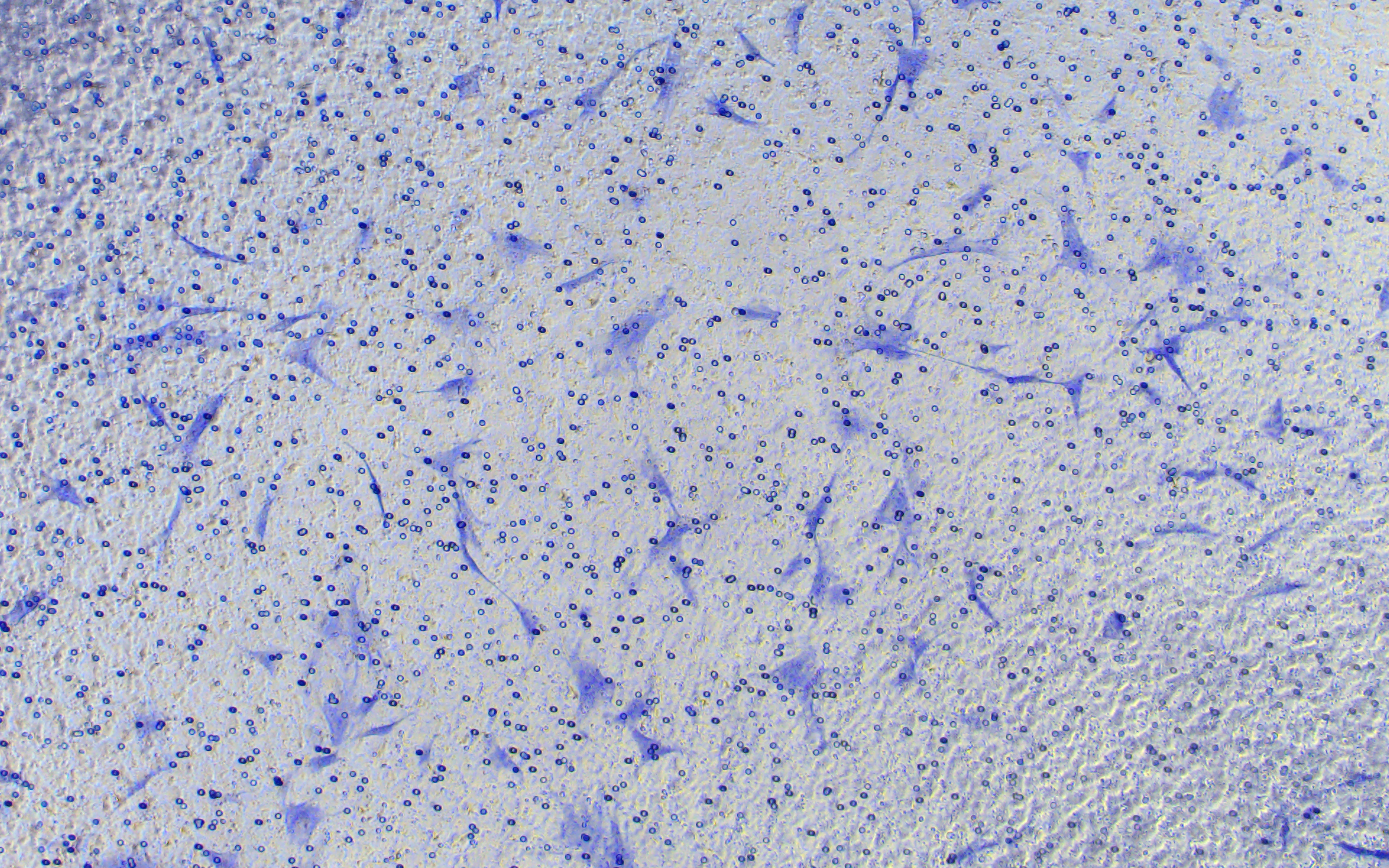

Supplement: S5 Dataset — (ZIP) [file pone.0261498.s005.zip › Fig 5/Fig 5 A2ú¿invasion-EPC supernatant).tif]

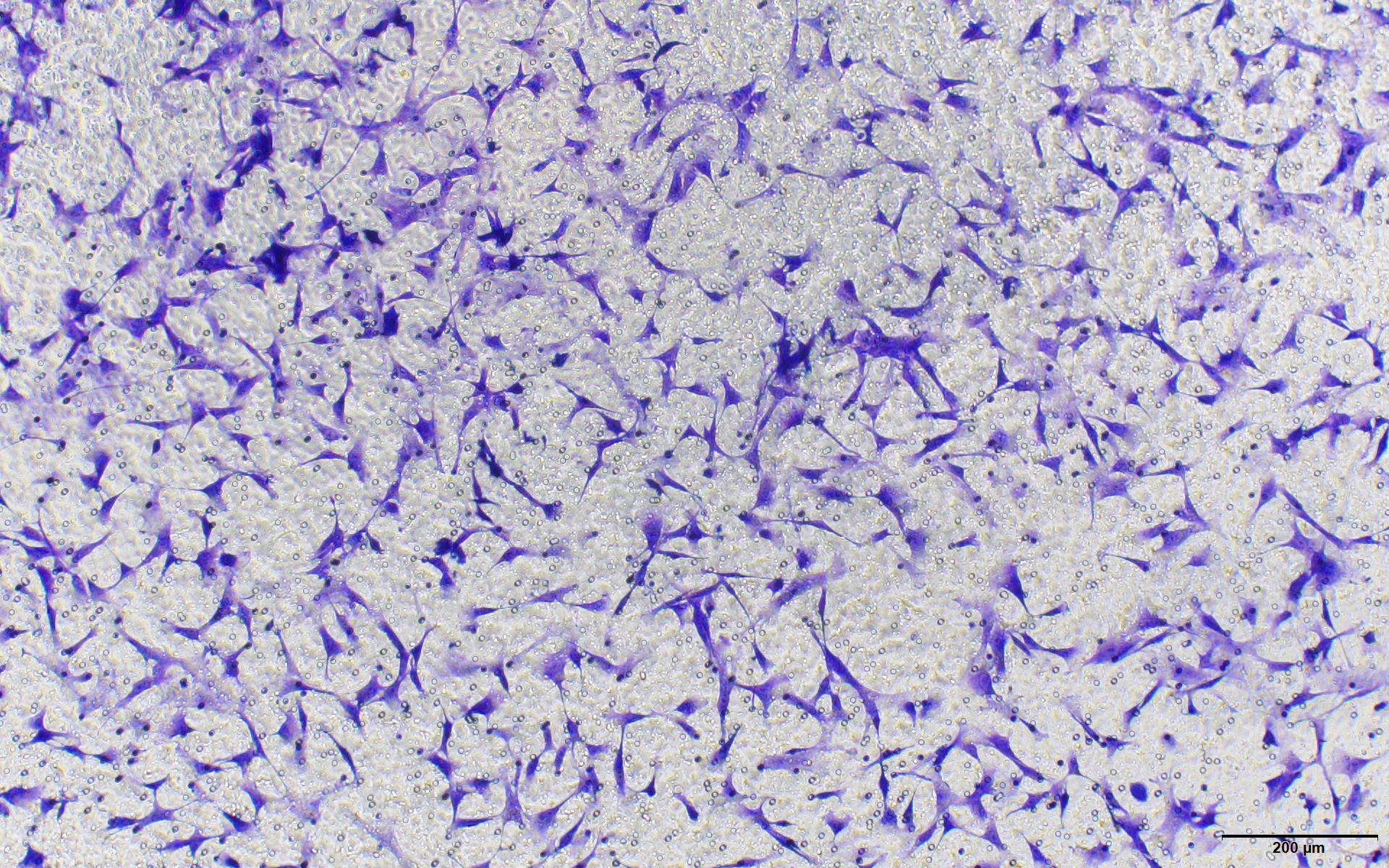

Supplement: S5 Dataset — (ZIP) [file pone.0261498.s005.zip › Fig 5/Fig 5 A3ú¿Transmigration-ASC supernatant).tif]

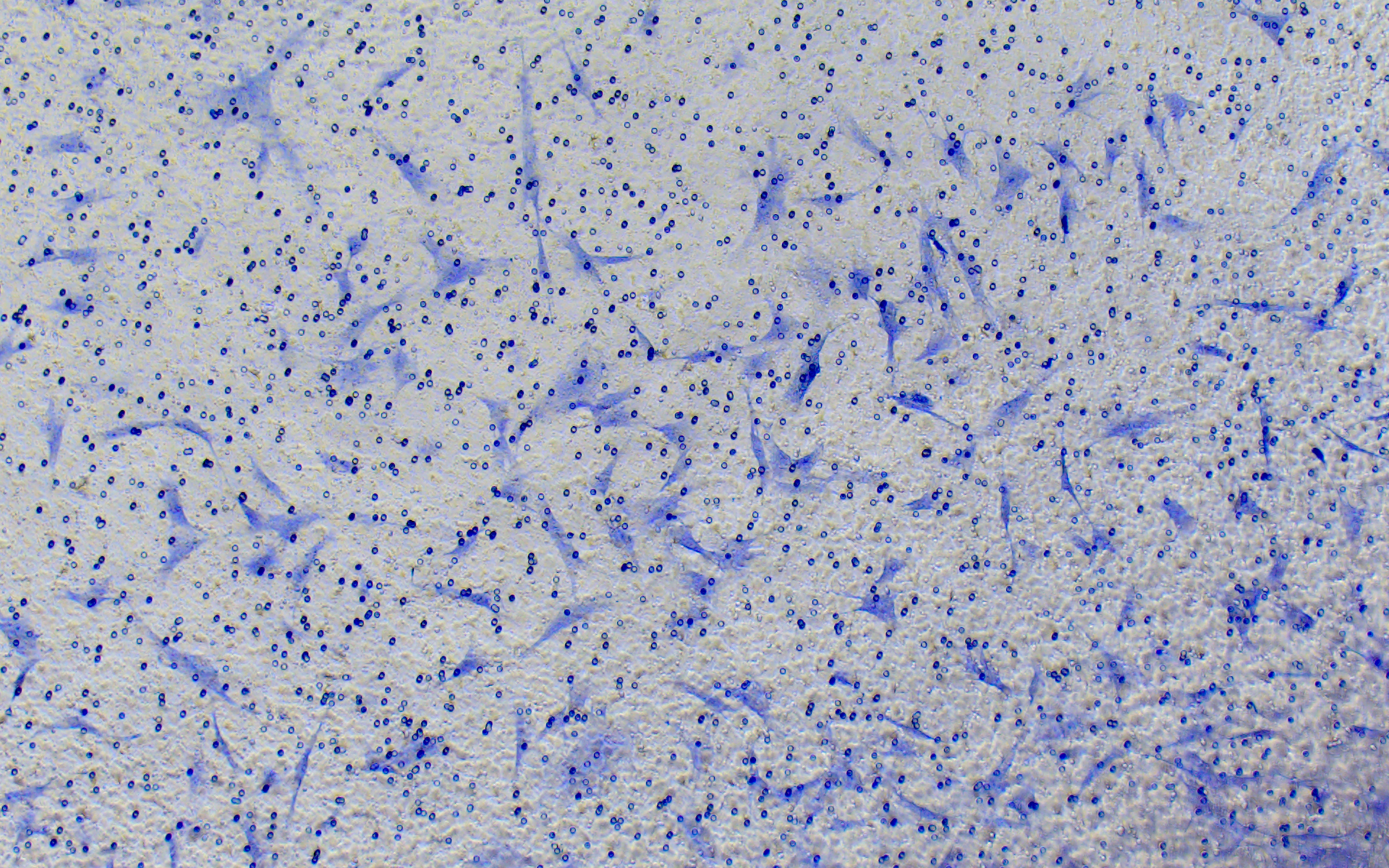

Supplement: S5 Dataset — (ZIP) [file pone.0261498.s005.zip › Fig 5/Fig 5 A3ú¿invasion-ASC supernatant).tif]

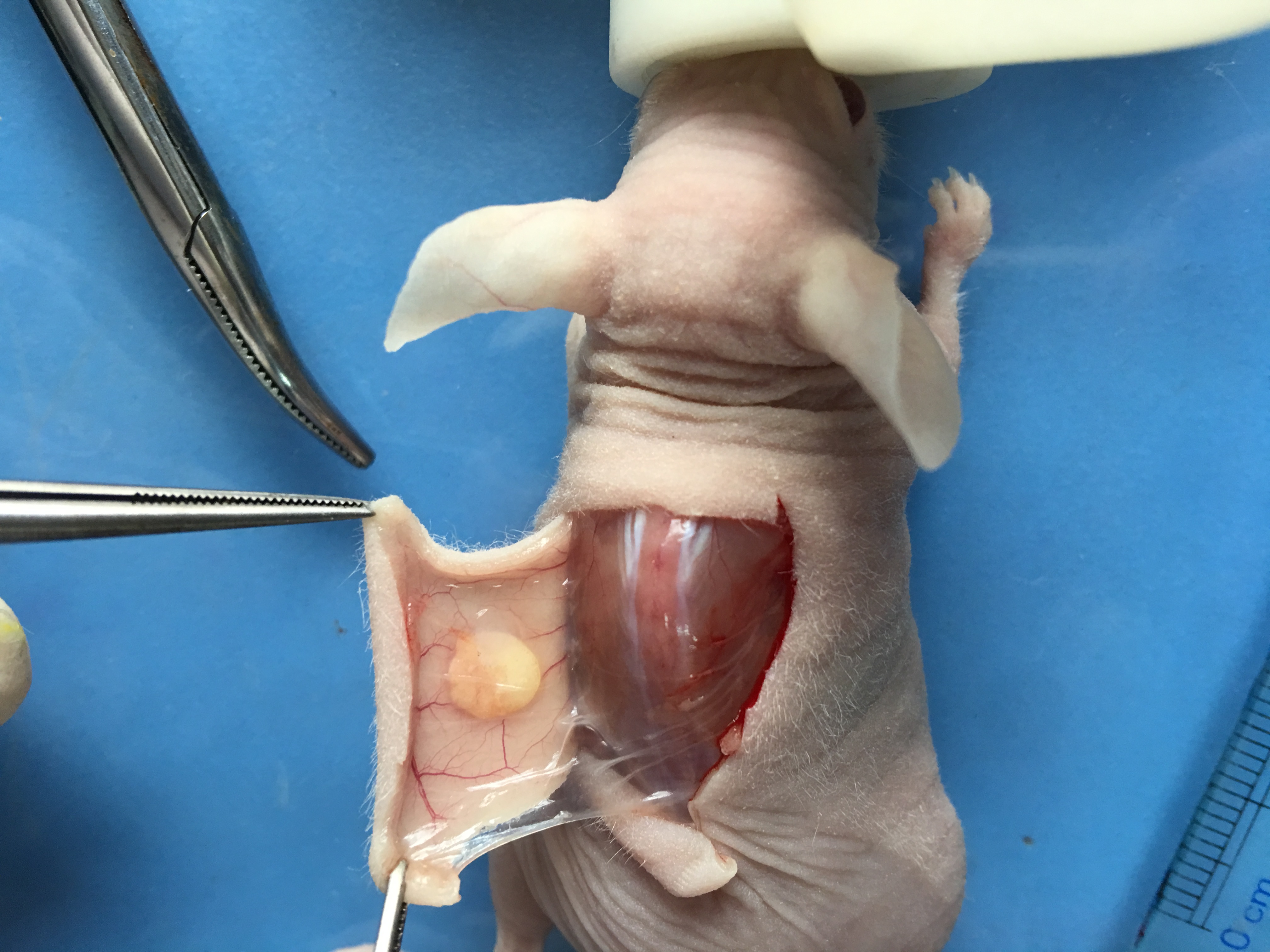

Supplement: S7 Dataset — (ZIP) [file pone.0261498.s007.zip › Fig 7/Fig 7 A.JPG]

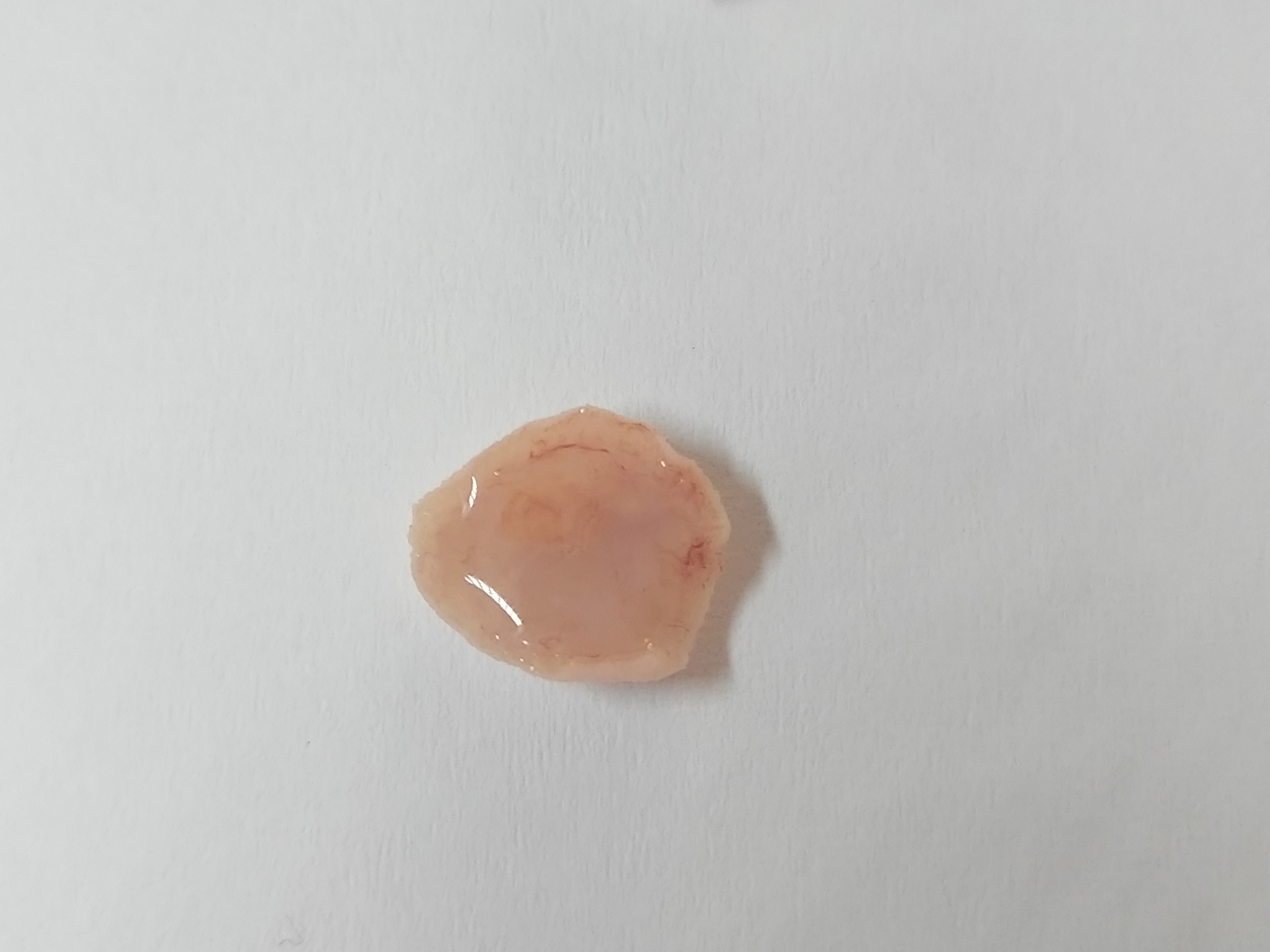

Supplement: S7 Dataset — (ZIP) [file pone.0261498.s007.zip › Fig 7/Fig 7 B1.jpg]

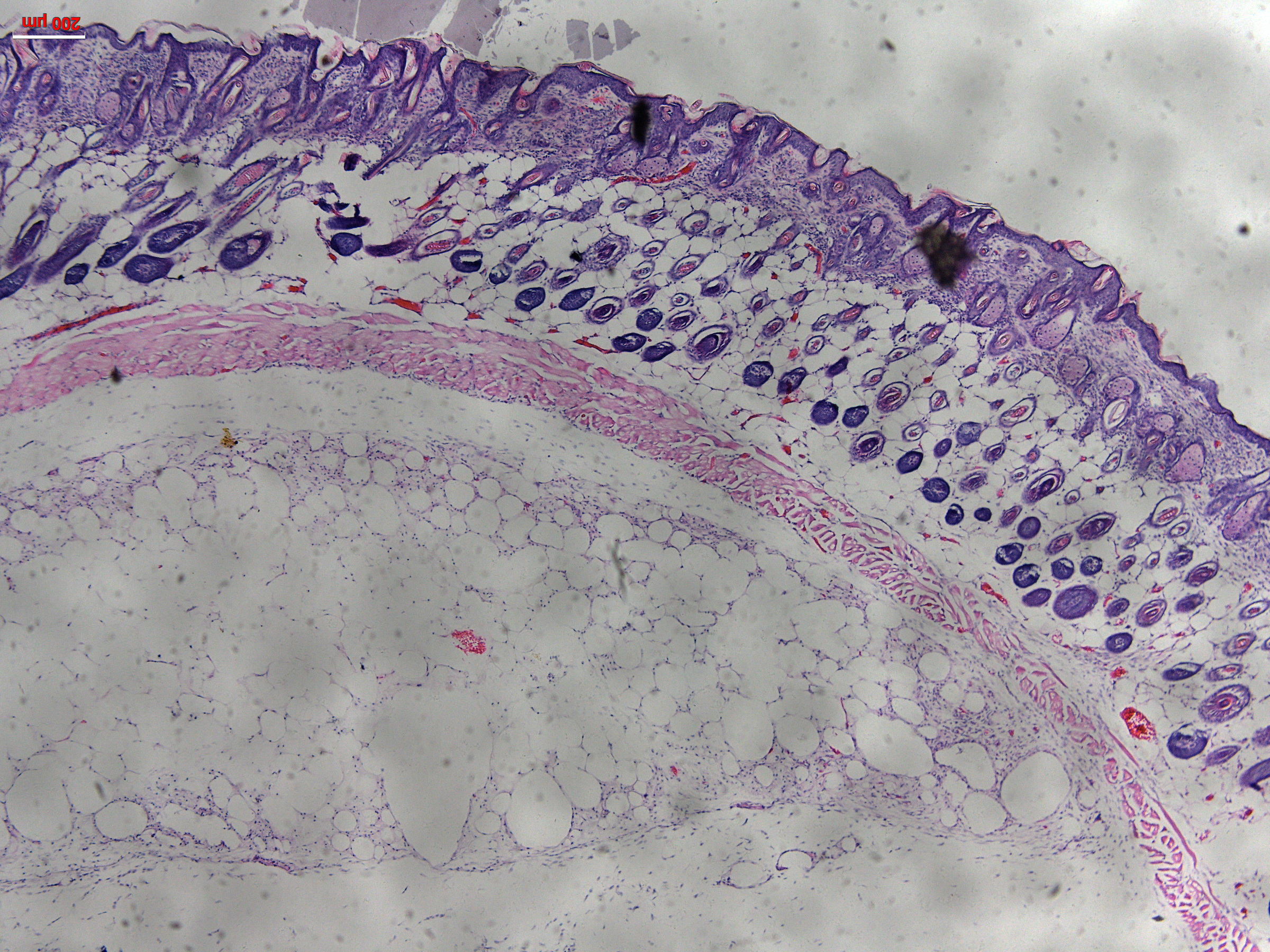

Supplement: S7 Dataset — (ZIP) [file pone.0261498.s007.zip › Fig 7/Fig 7 B2.tif]

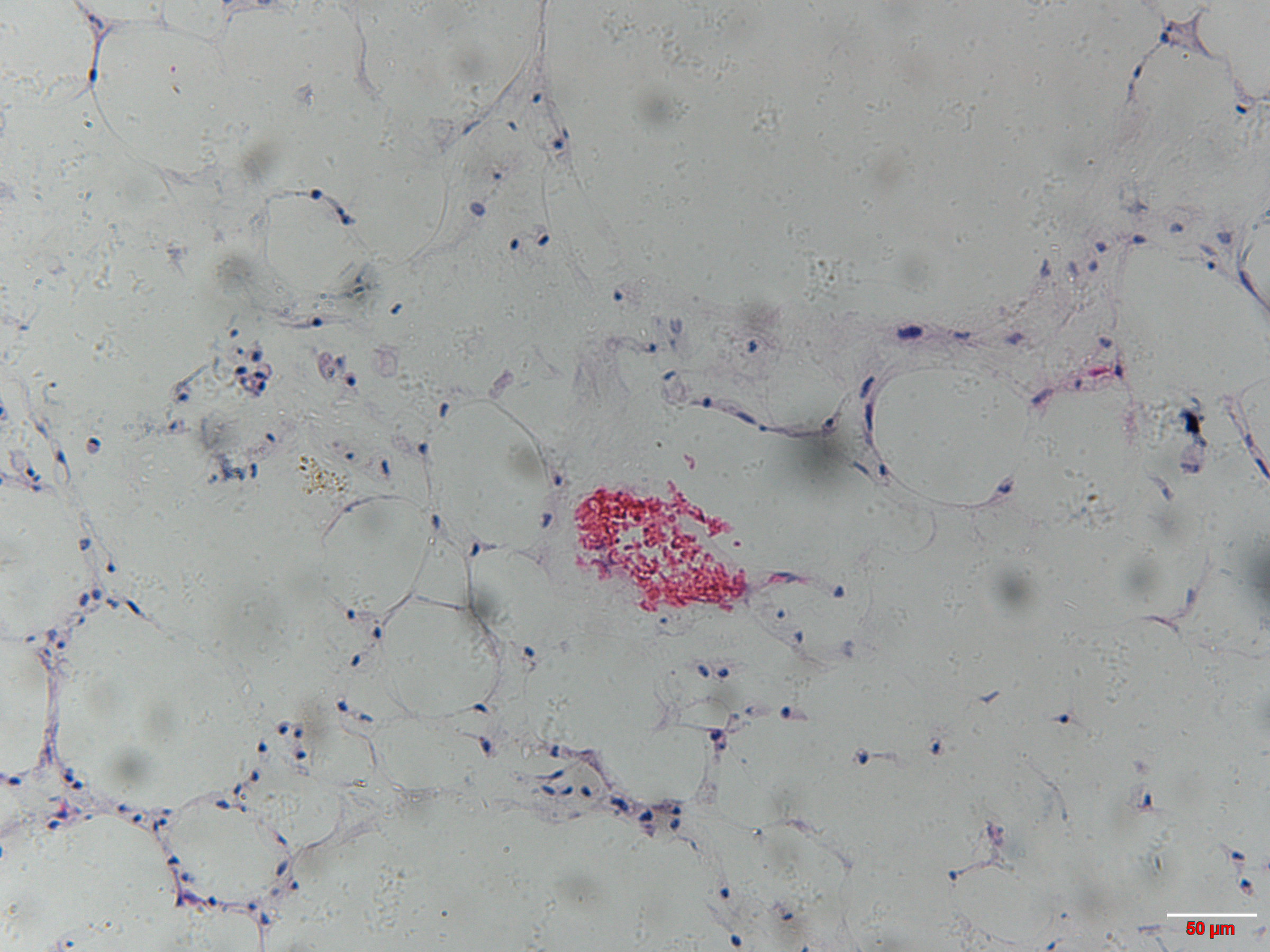

Supplement: S7 Dataset — (ZIP) [file pone.0261498.s007.zip › Fig 7/Fig 7 B3.tif]

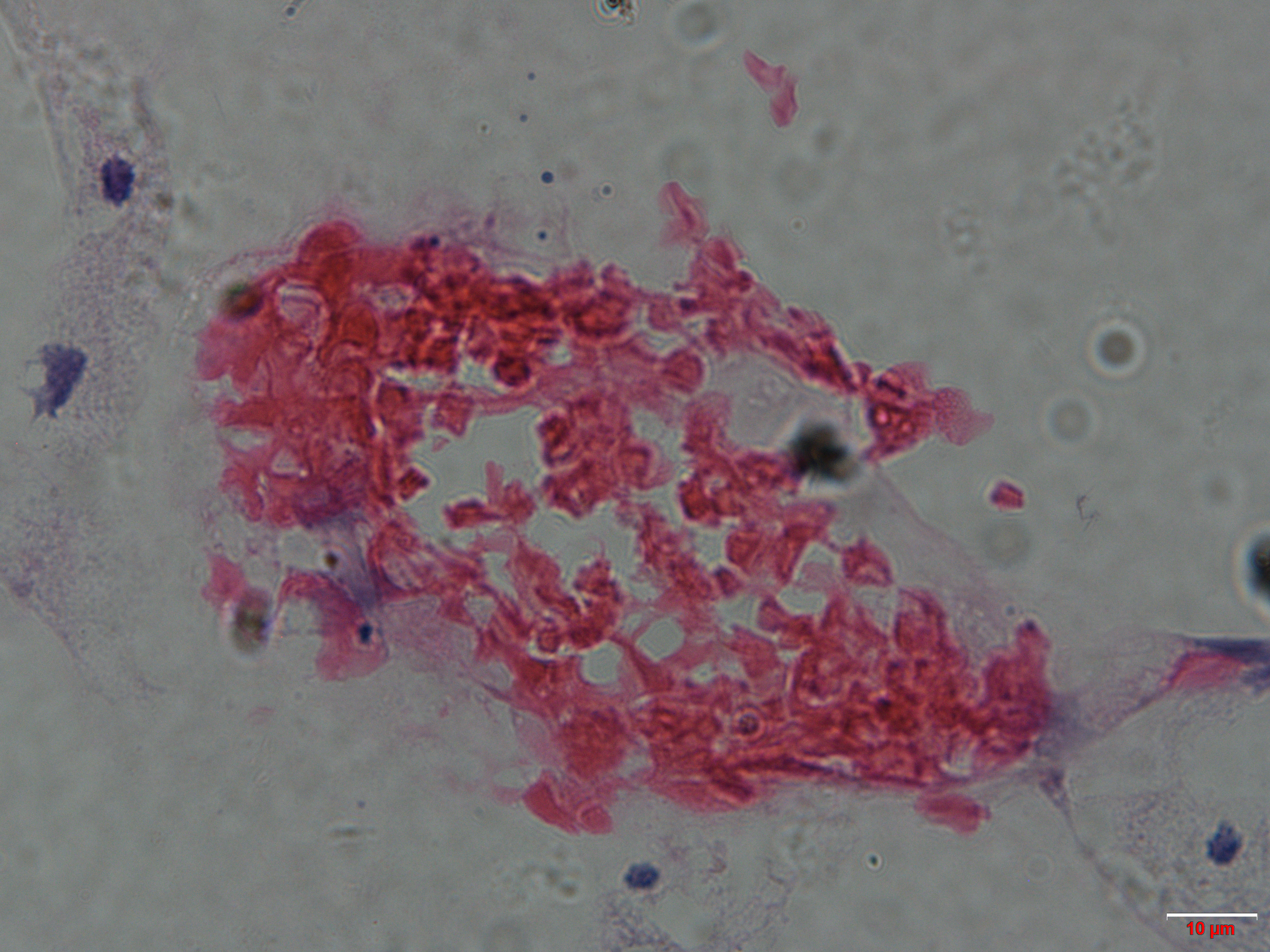

Supplement: S7 Dataset — (ZIP) [file pone.0261498.s007.zip › Fig 7/Fig 7 B4.tif]

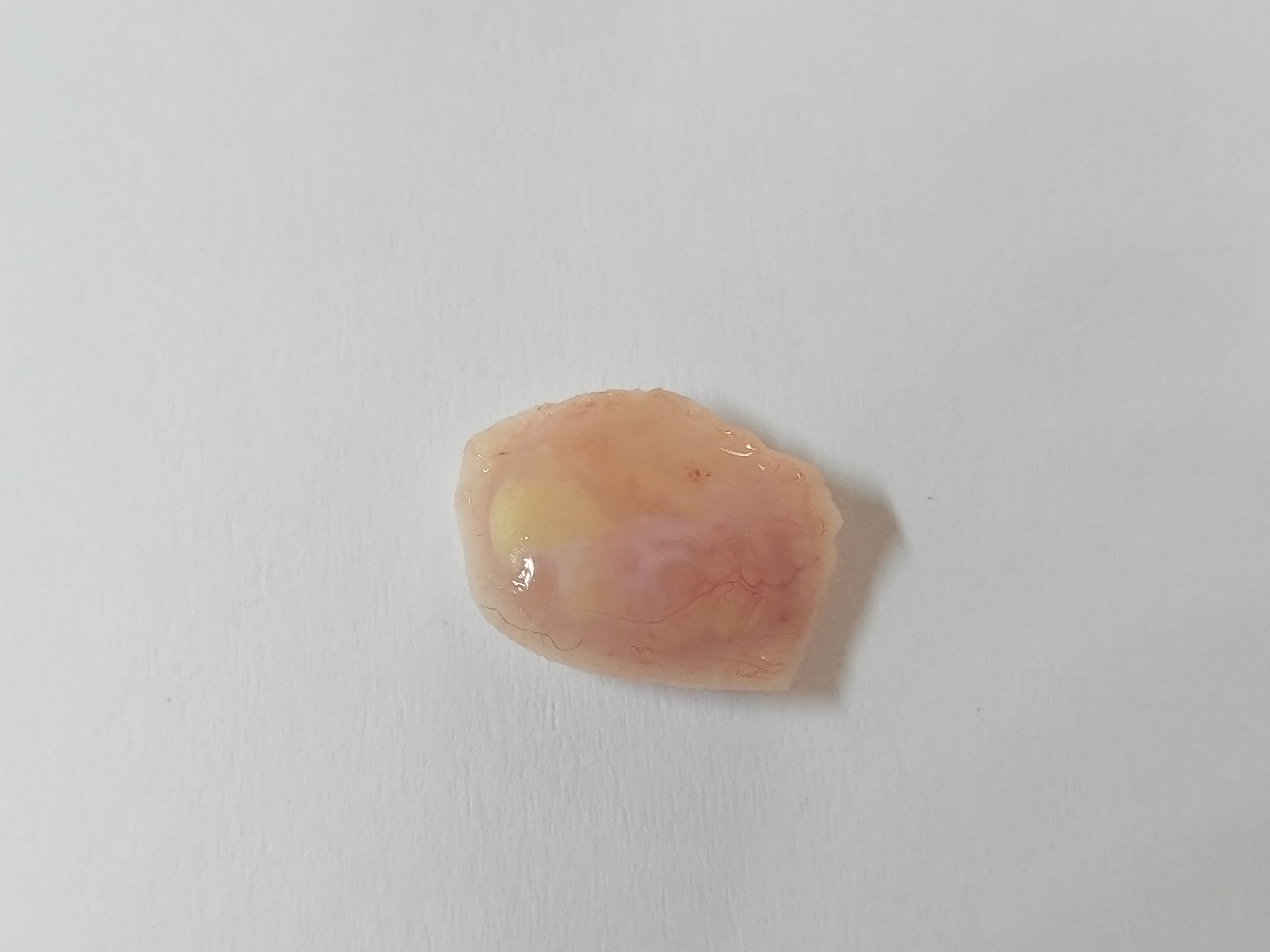

Supplement: S7 Dataset — (ZIP) [file pone.0261498.s007.zip › Fig 7/Fig 7 C1.jpg]

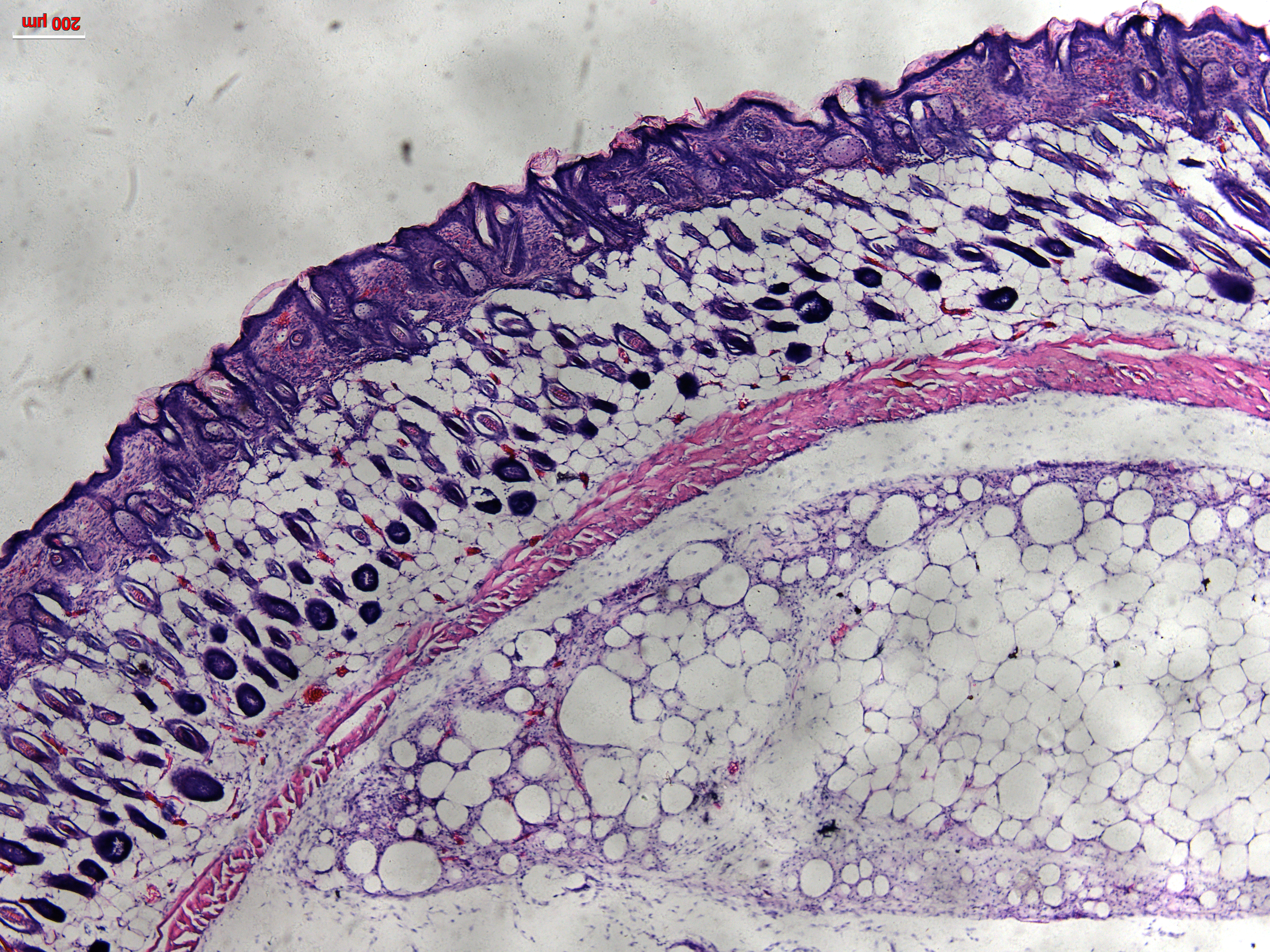

Supplement: S7 Dataset — (ZIP) [file pone.0261498.s007.zip › Fig 7/Fig 7 C2.tif]

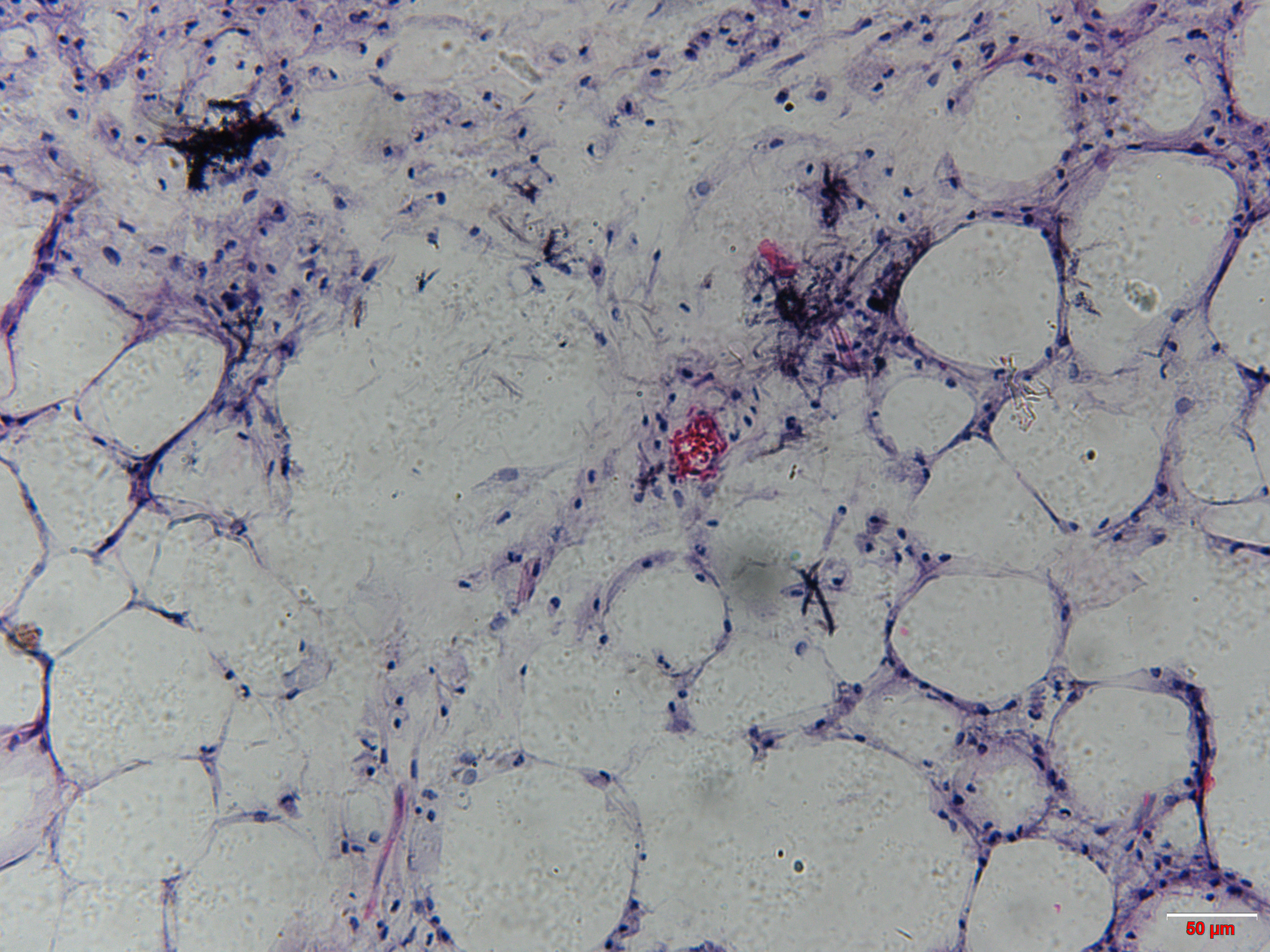

Supplement: S7 Dataset — (ZIP) [file pone.0261498.s007.zip › Fig 7/Fig 7 C3.tif]

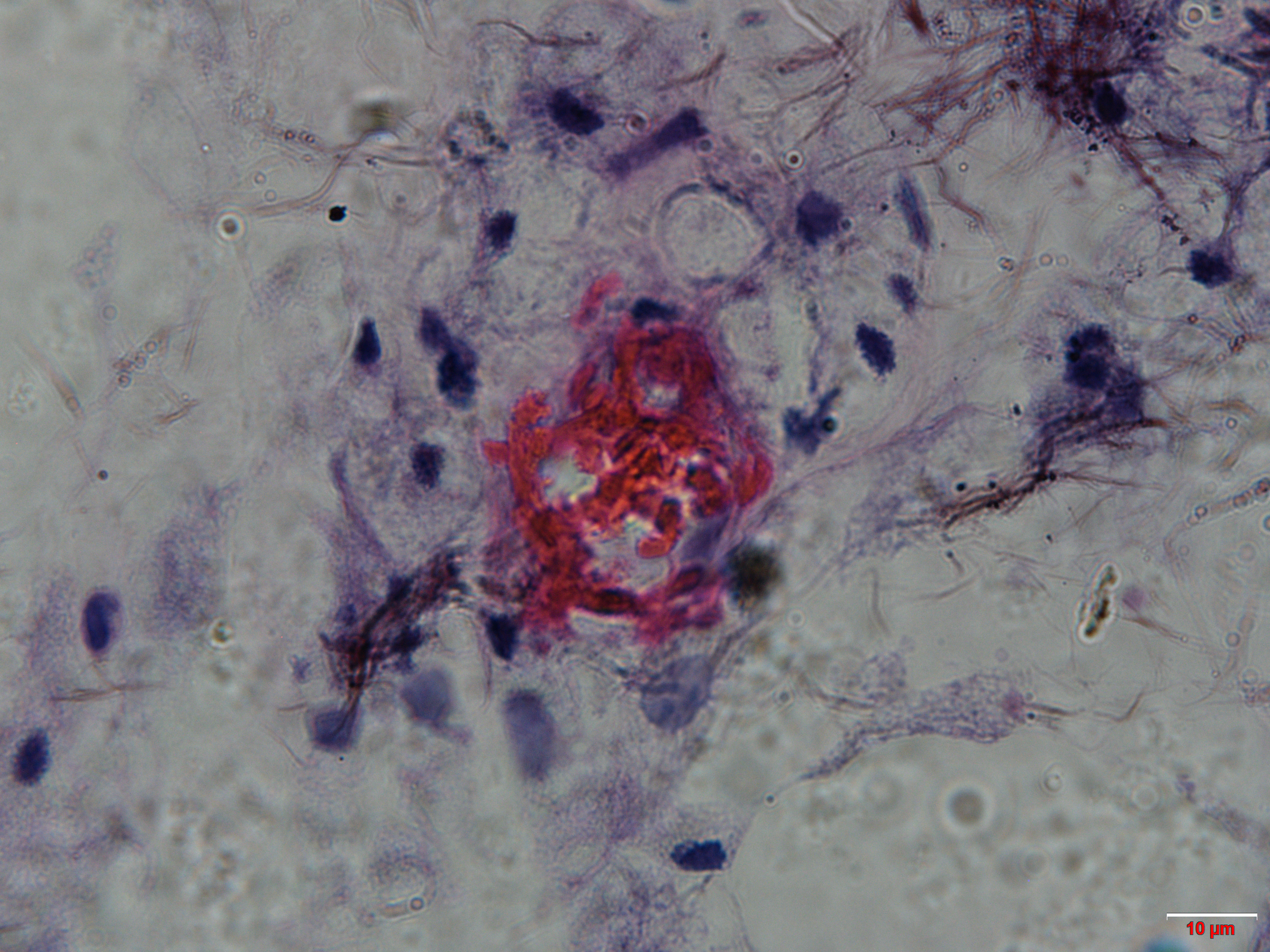

Supplement: S7 Dataset — (ZIP) [file pone.0261498.s007.zip › Fig 7/Fig 7 C4.tif]

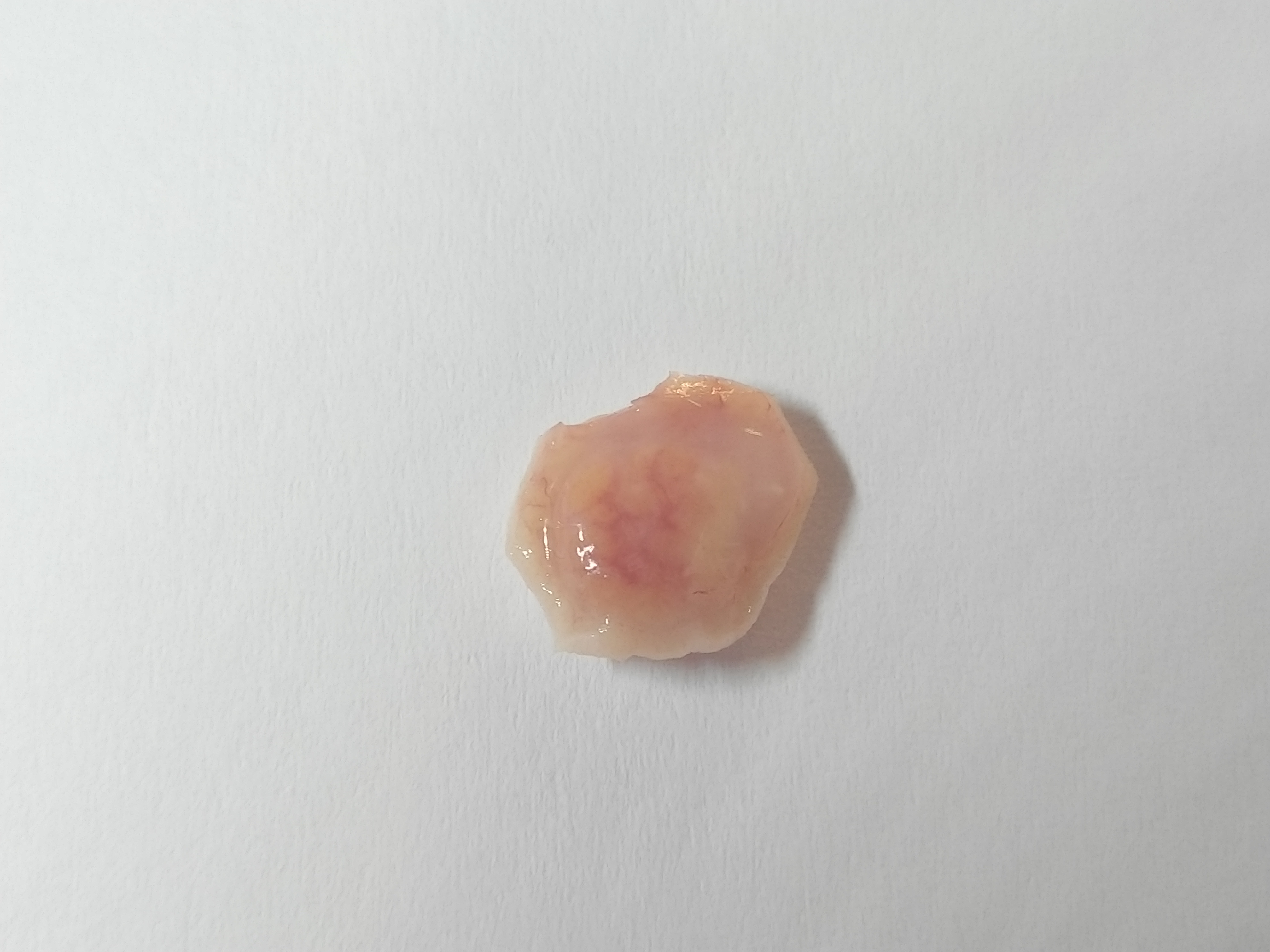

Supplement: S7 Dataset — (ZIP) [file pone.0261498.s007.zip › Fig 7/Fig 7 D1.jpg]

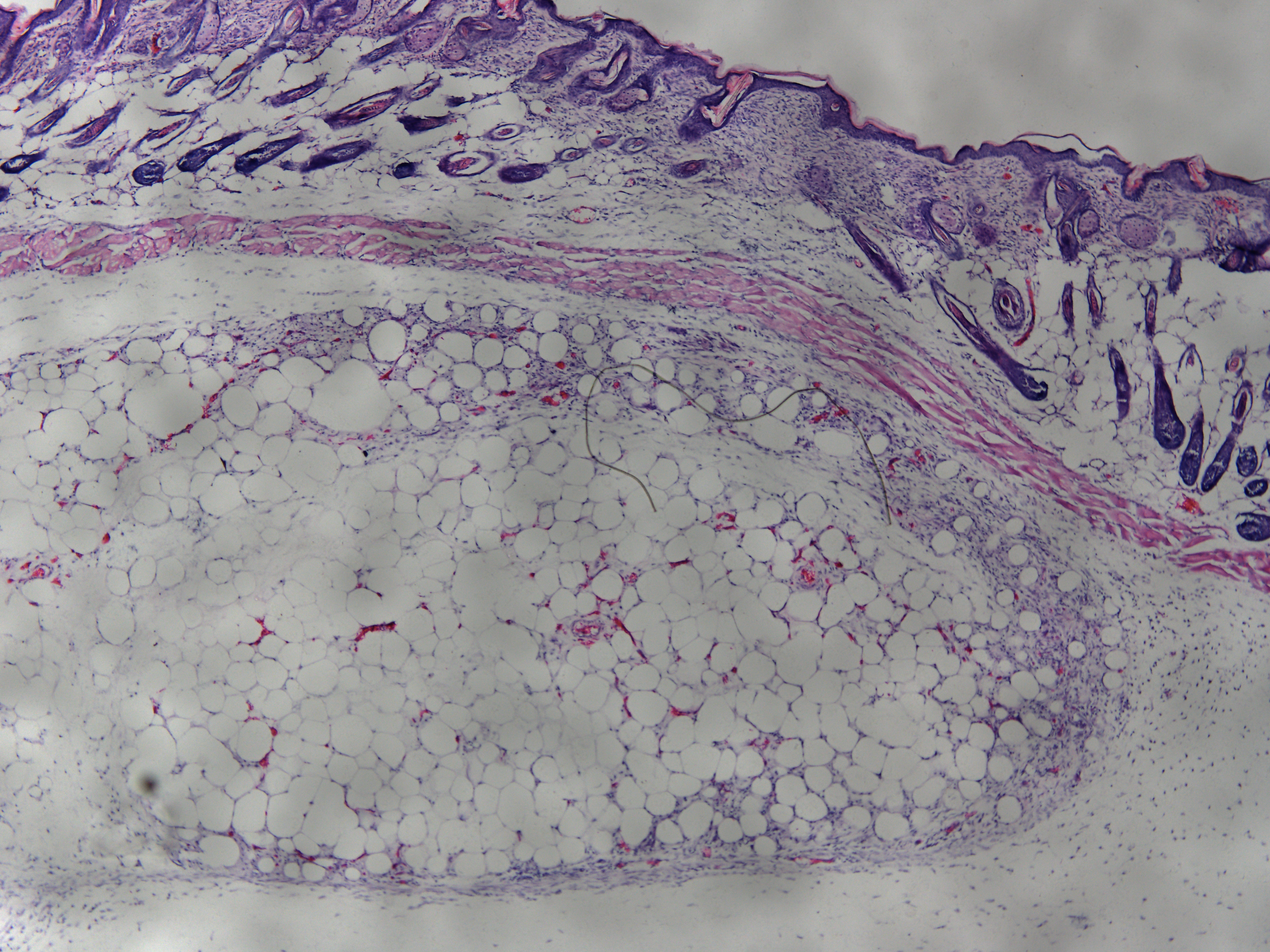

Supplement: S7 Dataset — (ZIP) [file pone.0261498.s007.zip › Fig 7/Fig 7 D2.tif]

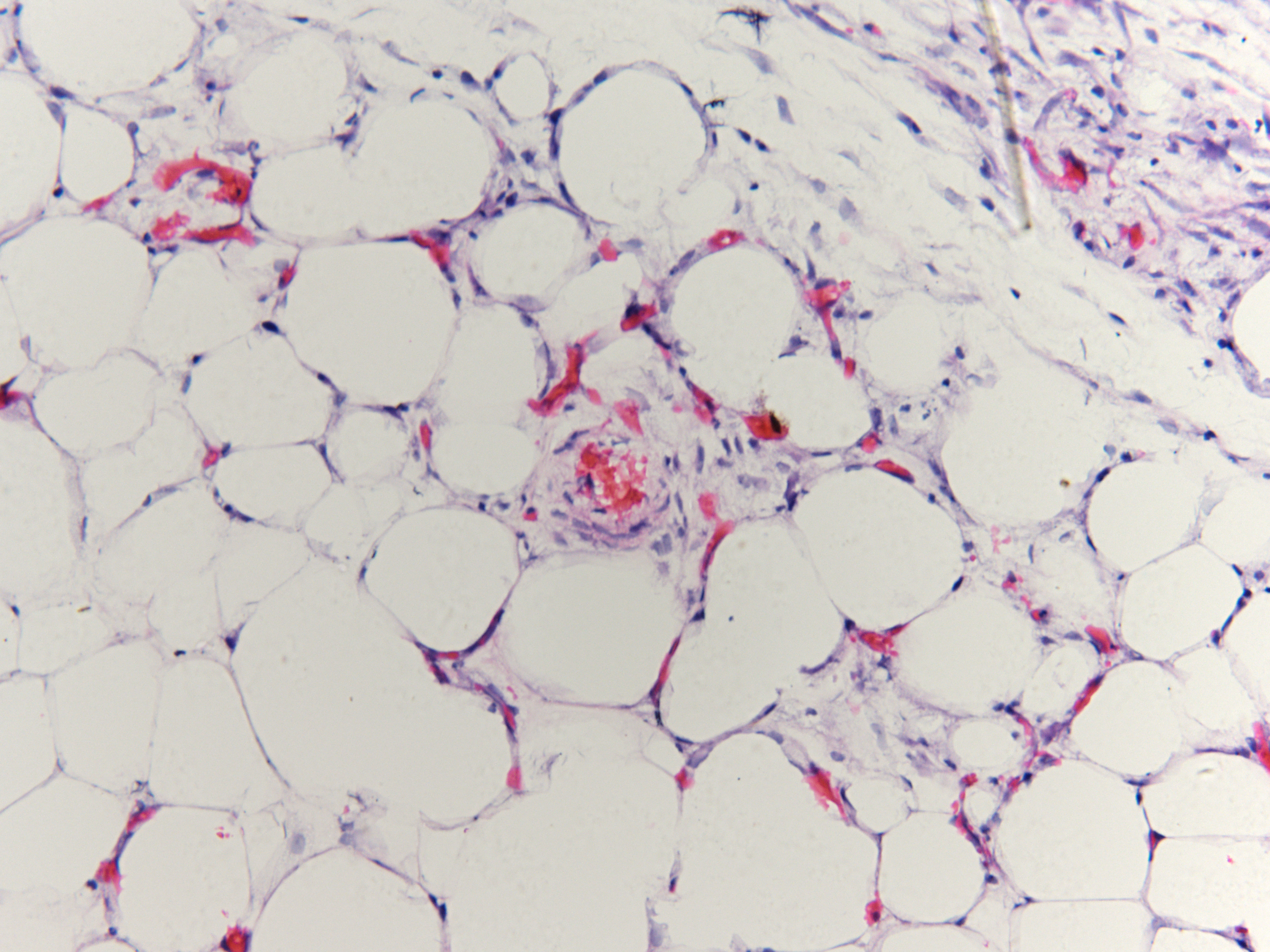

Supplement: S7 Dataset — (ZIP) [file pone.0261498.s007.zip › Fig 7/Fig 7 D3.tif]

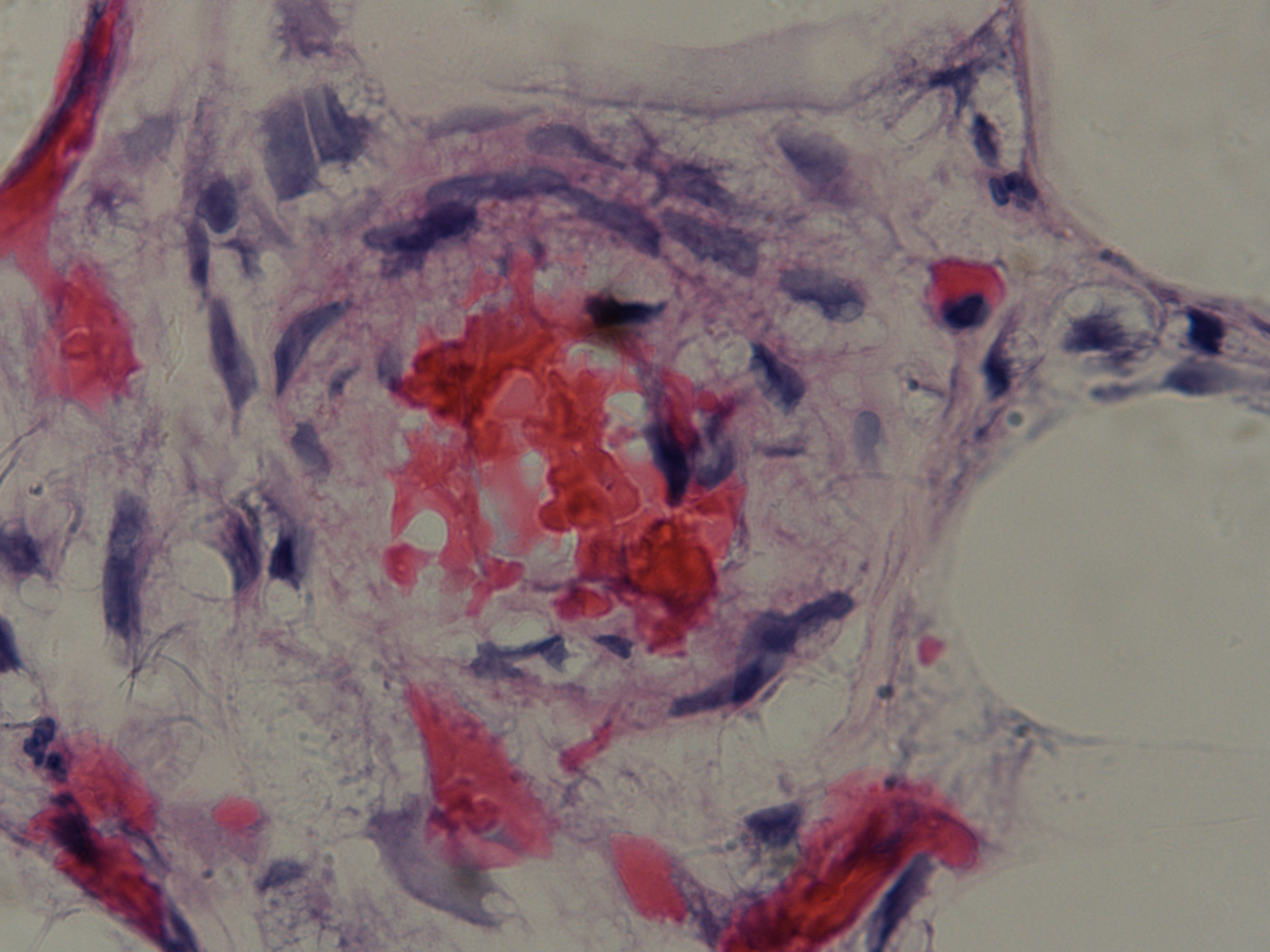

Supplement: S7 Dataset — (ZIP) [file pone.0261498.s007.zip › Fig 7/Fig 7 D4.tif]

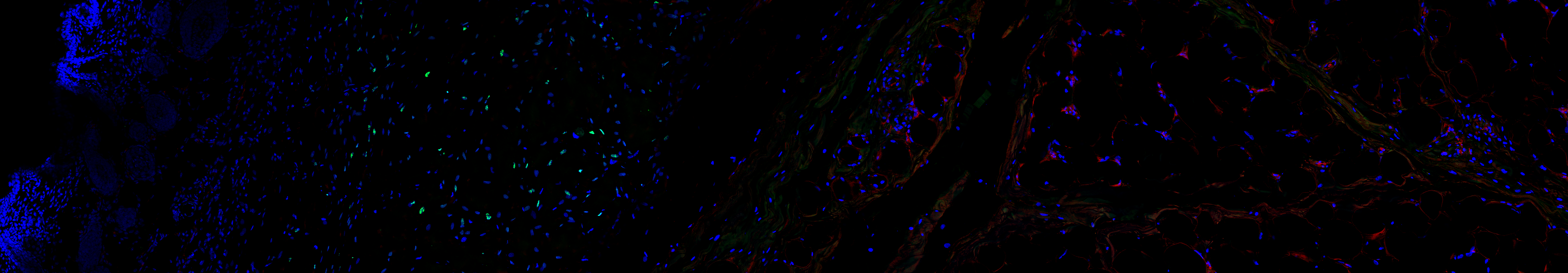

Supplement: S7 Dataset — (ZIP) [file pone.0261498.s007.zip › Fig 7/Fig 7 G.tif]

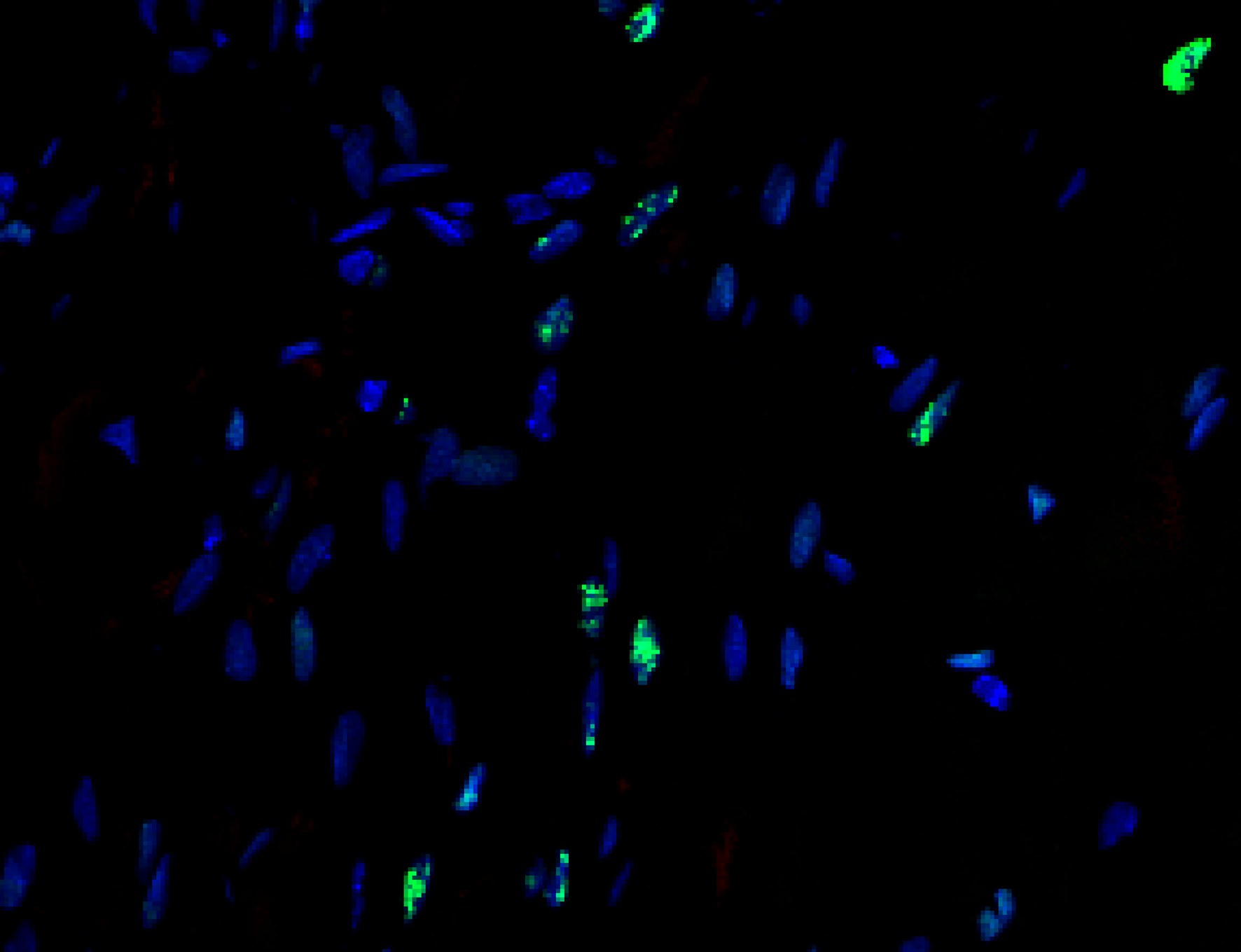

Supplement: S7 Dataset — (ZIP) [file pone.0261498.s007.zip › Fig 7/Fig 7 G1.tif]

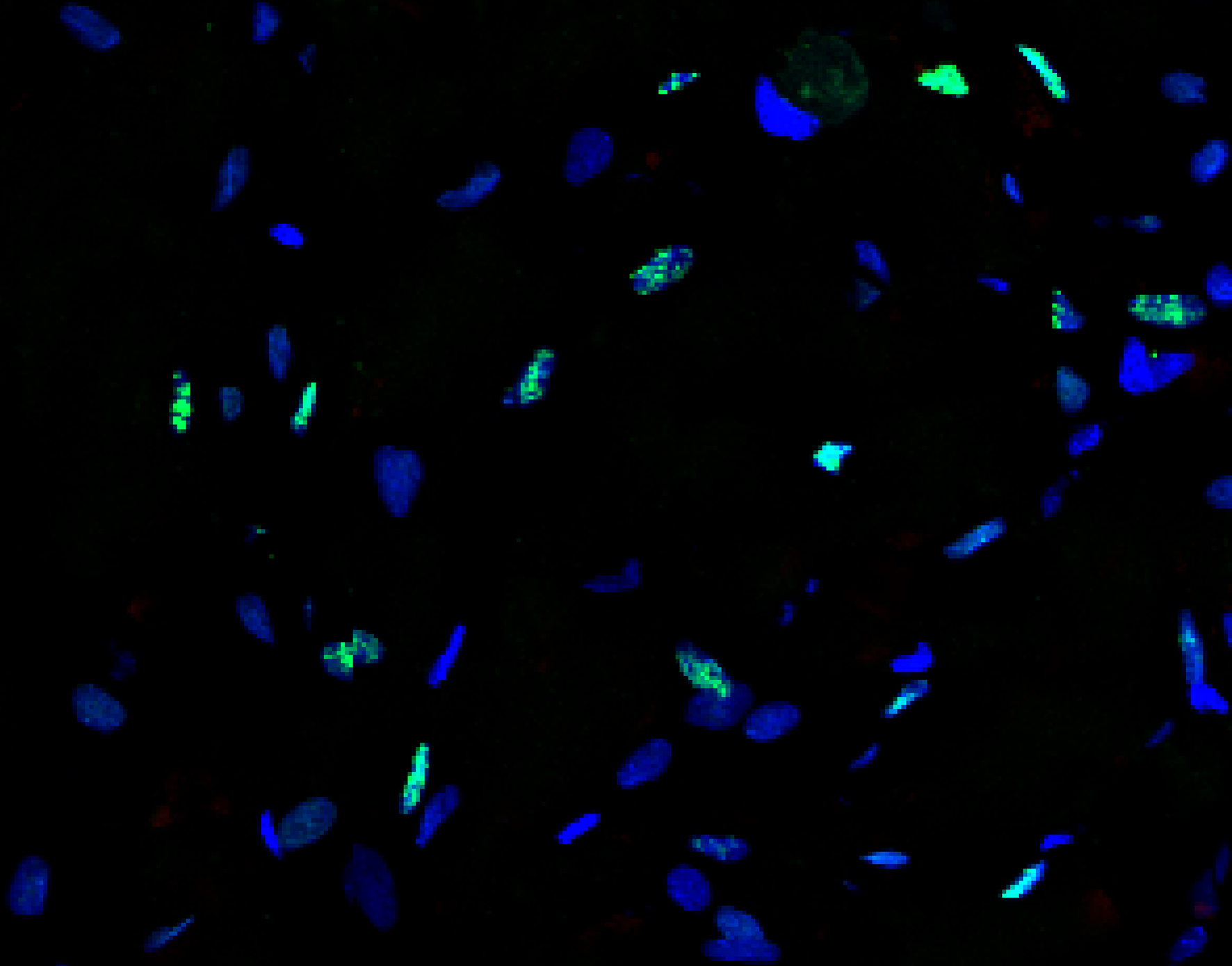

Supplement: S7 Dataset — (ZIP) [file pone.0261498.s007.zip › Fig 7/Fig 7 G2.tif]

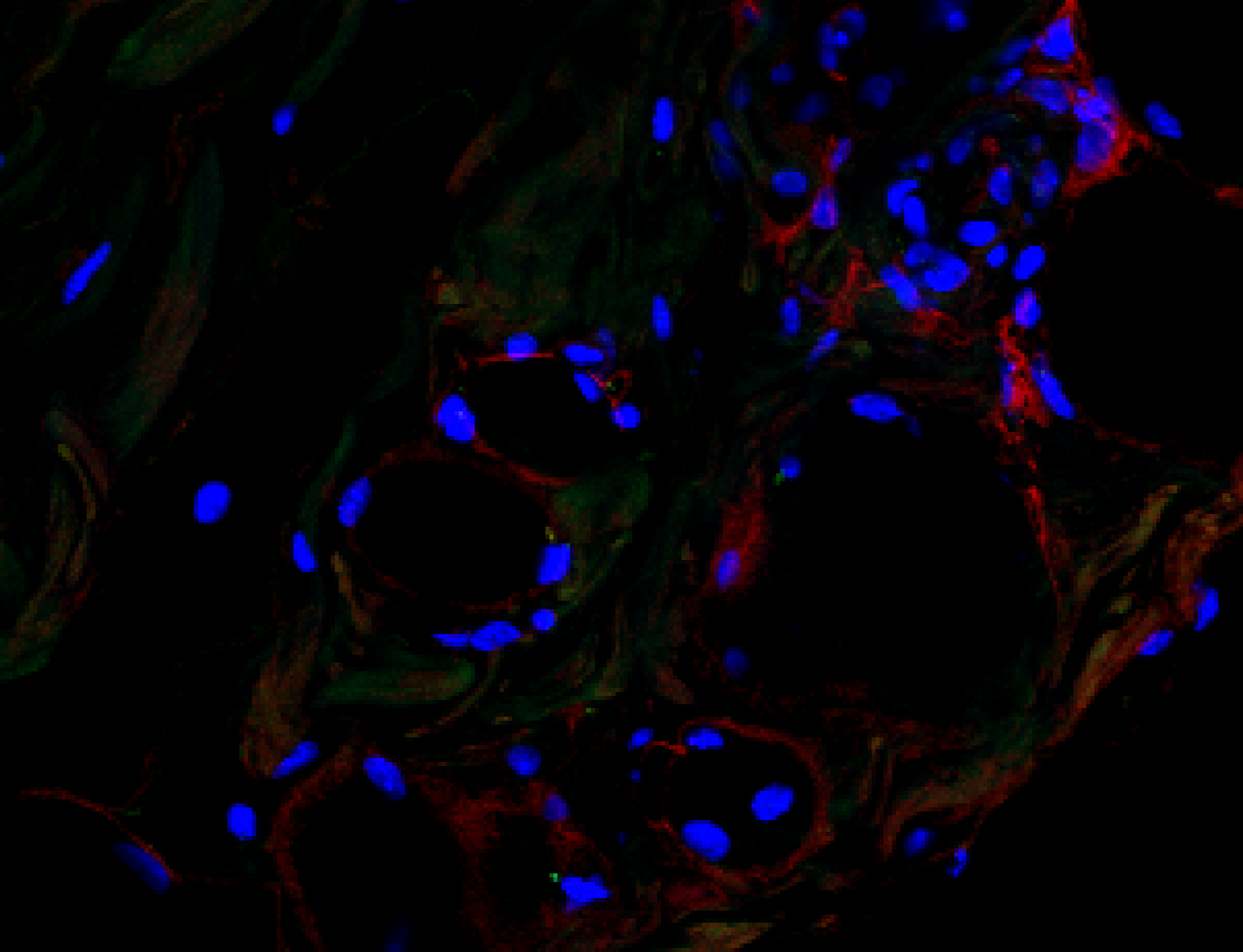

Supplement: S7 Dataset — (ZIP) [file pone.0261498.s007.zip › Fig 7/Fig 7 G3.tif]

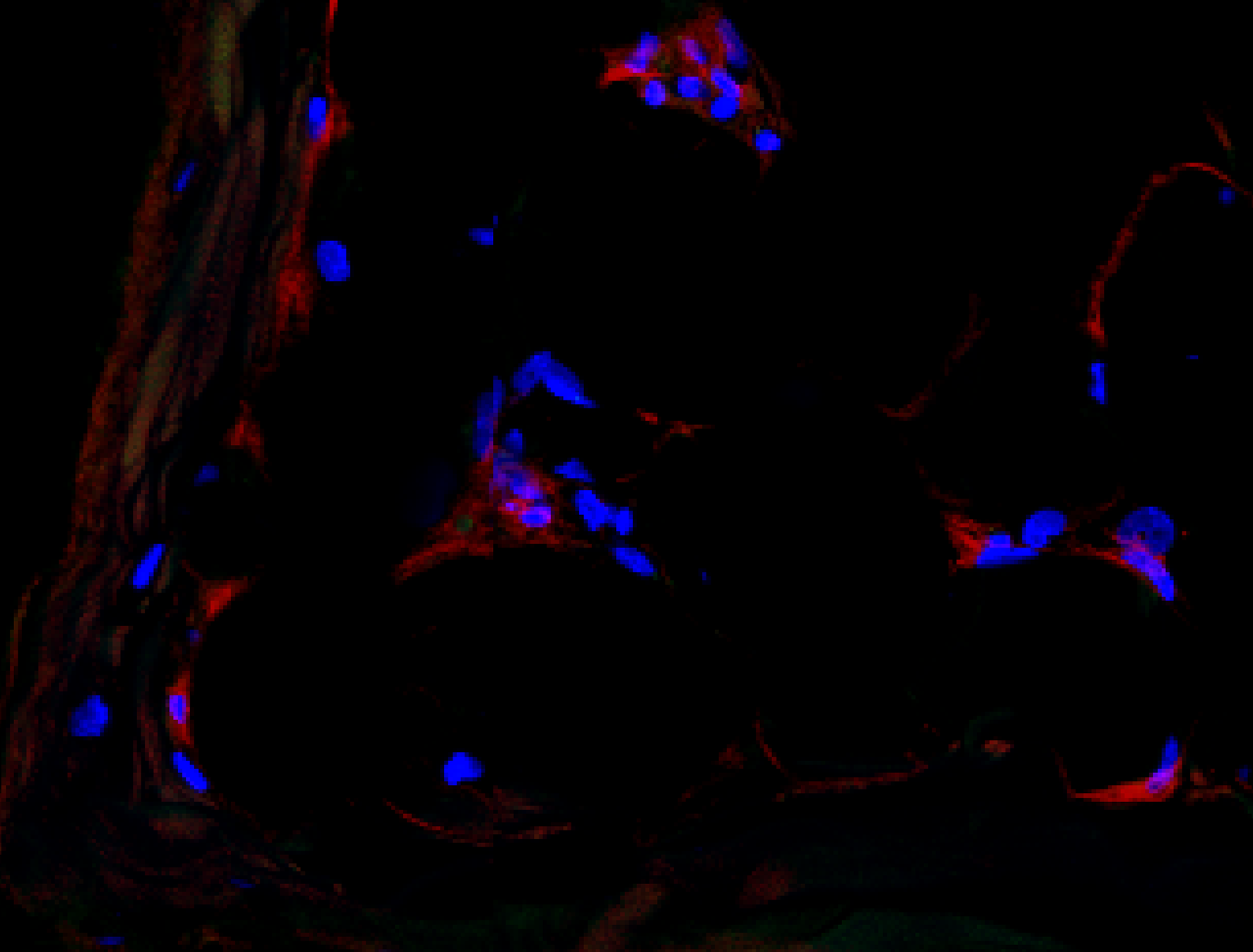

Supplement: S7 Dataset — (ZIP) [file pone.0261498.s007.zip › Fig 7/Fig 7 G4.tif]
